# The burden of mental disorders, substance use disorders and self-harm among young people in Europe, 1990–2019: Findings from the Global Burden of Disease Study 2019

**DOI:** 10.1016/j.lanepe.2022.100341

**Published:** 2022-04-01

**Authors:** Giulio Castelpietra, Ann Kristin Skrindo Knudsen, Emilie E. Agardh, Benedetta Armocida, Massimiliano Beghi, Kim Moesgaard Iburg, Giancarlo Logroscino, Rui Ma, Fabrizio Starace, Nicholas Steel, Giovanni Addolorato, Catalina Liliana Andrei, Tudorel Andrei, Jose L Ayuso-Mateos, Maciej Banach, Till Winfried Bärnighausen, Francesco Barone-Adesi, Akshaya Srikanth Bhagavathula, Felix Carvalho, Márcia Carvalho, Joht Singh Chandan, Vijay Kumar Chattu, Rosa A.S. Couto, Natália Cruz-Martins, Paul I. Dargan, Keshab Deuba, Diana Dias da Silva, Adeniyi Francis Fagbamigbe, Eduarda Fernandes, Pietro Ferrara, Florian Fischer, Peter Andras Gaal, Alessandro Gialluisi, Juanita A. Haagsma, Josep Maria Haro, M. Tasdik Hasan, Syed Shahzad Hasan, Sorin Hostiuc, Licia Iacoviello, Ivo Iavicoli, Elham Jamshidi, Jost B. Jonas, Tamas Joo, Jacek Jerzy Jozwiak, Srinivasa Vittal Katikireddi, Joonas H. Kauppila, Moien A.B. Khan, Adnan Kisa, Sezer Kisa, Mika Kivimäki, Kamrun Nahar Koly, Ai Koyanagi, Manasi Kumar, Tea Lallukka, Berthold Langguth, Caterina Ledda, Paul H. Lee, Ilaria Lega, Christine Linehan, Joana A. Loureiro, Áurea M Madureira-Carvalho, Jose Martinez-Raga, Manu Raj Mathur, John J. McGrath, Enkeleint A. Mechili, Alexios-Fotios A. Mentis, Tomislav Mestrovic, Bartosz Miazgowski, Andreea Mirica, Antonio Mirijello, Babak Moazen, Shafiu Mohammed, Francesk Mulita, Gabriele Nagel, Ionut Negoi, Ruxandra Irina Negoi, Vincent Ebuka Nwatah, Alicia Padron-Monedero, Songhomitra Panda-Jonas, Shahina Pardhan, Maja Pasovic, Jay Patel, Ionela-Roxana Petcu, Marina Pinheiro, Richard Charles G. Pollok, Maarten J. Postma, David Laith Rawaf, Salman Rawaf, Esperanza Romero-Rodríguez, Luca Ronfani, Dominic Sagoe, Francesco Sanmarchi, Michael P Schaub, Nigussie Tadesse Sharew, Rahman Shiri, Farhad Shokraneh, Inga Dora Sigfusdottir, João Pedro Silva, Renata Silva, Bogdan Socea, Miklós Szócska, Rafael Tabarés-Seisdedos, Marco Torrado, Marcos Roberto Tovani-Palone, Tommi Juhani Vasankari, Massimiliano Veroux, Russell M. Viner, Andrea Werdecker, Andrea Sylvia Winkler, Simon I. Hay, Alize J. Ferrari, Mohsen Naghavi, Peter Allebeck, Lorenzo Monasta

**Affiliations:** 1 Outpatient and Inpatient Care Service, Central Health Directorate, Region Friuli Venezia Giulia, Italy; 2 Centre for Disease Burden, Norwegian Institute of Public Health, Bergen, Norway; 3 Department of Global Public Health, Karolinska Institute, Stockholm, Sweden; 4 Department of Cardiovascular, Endocrine-metabolic Diseases and Aging, National Institute of Health, Rome, Italy; 5 Department of Mental Health, AUSL Romagna, Ravenna, Italy; 6 Department of Public Health, Aarhus University, Aarhus, Denmark; 7 Department of Basic Medical Sciences, Neuroscience and Sense Organs, University of Bari Aldo Moro, Bari, Italy; 8 Department of Clinical Research in Neurology, Fondazione Cardinale Giovanni Panico Hospital, Tricase, Italy; 9 Institute for Health Metrics and Evaluation, Department of Health Metrics Sciences, University of Washington, Seattle, WA, USA; 10 Department of Mental Health & Drug Abuse, AUSL Modena, Modena, Italy; 11 Department of Primary Care and Public Health, University of East Anglia, Norwich, UK; 12 Public Health England, London, UK; 13 Department of Internal Medicine, Catholic University of Rome, Rome, Italy; 14 Cardiology Department Carol Davila University of Medicine and Pharmacy, Bucharest, Romania; 15 Department of Statistics and Econometrics Bucharest Carol Davila University of Economic Studies, Bucharest, Romania; 16 CIBERSAM, Institute of Health Carlos III, Madrid, Spain; 17 Department of Hypertension, Medical University of Lodz, Lodz, Poland; 18 Heidelberg Institute of Global Health (HIGH), Heidelberg University, Heidelberg, Germany; 19 T.H. Chan School of Public Health, Harvard University, Boston, MA, USA; 20 Department of Translational Medicine, University of Eastern Piedmont, Novara, Italy; 21 Department of Social and Clinical Pharmacy, Charles University, Hradec Kralova, Czech Republic; 22 Institute of Public Health, United Arab Emirates University, Al Ain, United Arab Emirates; 23 Research Unit on Applied Molecular Biosciences (UCIBIO), University of Porto, Porto, Portugal; 24 Research Unit on Applied Molecular Biosciences (UCIBIO), University of Porto, Porto, Portugal; 25 Faculty of Health Sciences, University Fernando Pessoa, Porto, Portugal; 26 Institute of Applied Health Research, University of Birmingham, Birmingham, UK; 27 Faculty of Medical Sciences, University of the West Indies, St Augustine, Trinidad and Tobago; 28 Independent Consultant, Athens, Greece; 29 Department of Chemical Sciences, University of Porto, Porto, Portugal; 30 Department of Medicine (Prof N Cruz-Martins PhD), University of Porto, Porto, Portugal; 31 Department of Health Sciences Institute of Research and Advanced Training in Health Sciences and Technologies (CESPU), Famalicão, Portugal; 32 Faculty of Life Sciences and Medicine, King's College London, London, UK; 33 Department of Clinical Toxicology, Guy's and St. Thomas' NHS Foundation Trust, London, UK; 34 National Centre for AIDS and STD Control, Save the Children, Kathmandu, Nepal; 35 Department of Global Public Health, Karolinska Institute, Stockholm, Sweden; 36 Laboratory of Toxicology, University of Porto, Porto, Portugal; 37 Epidemiology and Medical Statistics, University of Ibadan, Ibadan, Nigeria; 38 Population and Behavioural Sciences, University of St Andrews, St Andrews, UK; 39 Associated Laboratory for Green Chemistry (LAQV), University of Porto, Porto, Portugal; 40 Research Center on Public Health, University of Milan Bicocca, Monza, Italy; 41 Institute of Public Health, Charité Universitätsmedizin Berlin (Charité Medical University Berlin), Berlin, Germany; 42 Health Services Management Training Centre, Semmelweis University, Budapest, Hungary; 43 Department of Applied Social Sciences, Sapientia Hungarian University of Transylvania, Târgu-Mureş, Romania; 44 Department of Epidemiology and Prevention, IRCCS Neuromed, Pozzilli, Italy; 45 Department of Public Health, Erasmus University Medical Center, Rotterdam, Netherlands; 46 Research Unit, University of Barcelona, Barcelona, Spain; 47 Biomedical Research Networking Center for Mental Health Network (CiberSAM), Barcelona, Spain; 48 Department of Pharmacy, University of Huddersfield, Huddersfield, UK; 49 School of Biomedical Sciences and Pharmacy, University of Newcastle, Newcastle, NSW, Australia; 50 Department of Primary Care and Mental Health, University of Liverpool, Liverpool, UK; 51 Department of Legal Medicine and Bioethics, Carol Davila University of Medicine and Pharmacy, Bucharest, Romania; 52 Clinical Legal Medicine Department, National Institute of Legal Medicine Mina Minovici, Bucharest, Romania; 53 Department of Epidemiology and Prevention, IRCCS Neuromed, Pozzilli, Italy; 54 Research Center in Epidemiology and Preventive Medicine (EPIMED), University of Insubria, Varese, Italy; 55 Department of Public Health, University of Naples Federico II, Naples, Italy; 56 Functional Neurosurgery Research Center, Shahid Beheshti University of Medical Sciences, Tehram, Iran; 57 Division of Pulmonary Medicine, University of Lausanne (UNIL), Lausanne, Switzerland; 58 Institute of Molecular and Clinical Ophthalmology Basel, Basel, Switzerland; 59 Department of Ophthalmology, Heidelberg University, Mannheim, Germany; 60 Health Services Management Training Centre, Semmelweis University, Budapest, Hungary; 61 Department of Family Medicine and Public Health, University of Opole, Opole, Poland; 62 MRC/CSO Social and Public Health Sciences Unit, University of Glasgow, Glasgow, UK; 63 Department of Molecular Medicine and Surgery, Karolinska Institute, Stockholm, Sweden; 64 Surgery Research Unit, University of Oulu, Oulu, Finland; 65 Family Medicine Department, United Arab Emirates University, Al Ain, United Arab Emirates; 66 Primary Care Department, NHS North West London, London, UK; 67 School of Health Sciences, Kristiania University College, Oslo, Norway;; 68 Department of Global Community Health and Behavioral Sciences, Tulane University, New Orleans, LA, USA; 69 Department of Nursing and Health Promotion, Oslo Metropolitan University, Oslo, Norway; 70 Department of Epidemiology and Public Health University College London, London, UK; 71 Department of Public Health, University of Helsinki, Helsinki, Finland; 72 Health System and Population Studies Divisions, International Centre for Diarrhoeal Disease Research, Dhaka, Bangladesh; 73 Center for Global Mental Health, London School of Hygiene & Tropical Medicine, London, UK; 74 Biomedical Research Networking Center for Mental Health Network (CIBERSAM), San Juan de Dios Sanitary Park, Sant Boi de Llobregat, Spain; 75 Catalan Institution for Research and Advanced Studies (ICREA), Barcelona, Spain; 76 Division of Psychology and Language Sciences, University College London, London, UK; 77 Department of Psychiatry, University of Nairobi, Nairobi, Kenya; 78 Department of Public Health, University of Helsinki, Helsinki, Finland; 79 Department of Psychiatry and Psychotherapy, University of Regensburg, Regensburg, Germany; 80 Clinical and Experimental Medicine, University of Catania, Catania, Italy; 81 Department of Health Sciences, University of Leicester, Leicester, UK; 82 National Center for Disease Prevention and Health Promotion, Istituto Superiore di Sanità - Italian National Insitute of Health, Rome, Italy; 83 UCD Centre for Disability Studies, University College Dublin, Dublin, Ireland; 84 Laboratory for Process Engineering, Environment, Biotechnology and Energy (LEPABE) University of Porto, Porto, Portugal; 85 School of Health, Polytechnic Institute of Porto, Portugal;; 86 Associated Laboratory for Green Chemistry (LAQV), University of Porto, Porto, Portugal; 87 Institute for Research and Advanced Training in Health Sciences and Technologies, Instituto Universitário de Ciências da Saúde (University Institute of Health Sciences), Gandra, Portugal; 88 Psychiatry Department, Hospital Universitario Doctor Peset, Valencia, Spain; 89 Department of Medicine, University of Valencia, Valencia, Spain; 90 Health Policy Research Public Health Foundation of India, Gurugram, India; 91 Institute of Population Health Sciences, University of Liverpool, Liverpool, UK; 92 Queensland Brain Institute, School of Public Health, The University of Queensland, Brisbane, QLD, Australia; 93 National Centre for Register-based Research, Aarhus University, Aarhus, Denmark; 94 Department of Healthcare, University of Vlora, Vlora city, Albania; 95 Clinic of Social and Family Medicine, University of Crete, Heraklion, Greece; 96 University Research Institute, National and Kapodistrian University of Athens, Athens, Greece; 97 Clinical Microbiology and Parasitology Unit, Dr. Zora Profozic Polyclinic, Zagreb, Croatia; 98 University Centre Varazdin, University North, Varazdin, Croatia; 99 Center for Innovation in Medical Education, Pomeranian Medical University, Szczecin, Poland; 100 Department of Statistics and Econometrics Bucharest University of Economic Studies, Bucharest, Romania; 101 Department of Medical Sciences IRCCS Casa Sollievo della Sofferenza General Hospital, San Giovanni Rotondo, Italy; 102 Heidelberg Institute of Global Health (HIGH), Heidelberg University, Heidelberg, Germany; 103 Institute of Addiction Research (ISFF), Frankfurt University of Applied Sciences, Frankfurt, Germany; 104 Health Systems and Policy Research Unit, Ahmadu Bello University, Zaria, Nigeria; 105 Department of Health Care Management, Technical University of Berlin, Berlin, Germany; 106 Department of Surgery, General University Hospital of Patras, Patras, Greece; 107 Medical School, University of Thessaly, Larissa, Greece; 108 Institute of Epidemiology and Medical Biometry Ulm University, Ulm, Germany; 109 Department of General Surgery Carol Davila University of Medicine and Pharmacy, Bucharest, Romania; 110 Department of General Surgery, Emergency Hospital of Bucharest, Bucharest, Romania; 111 Department of Anatomy and Embryology Romania, Bucharest, Romania; 112 Cardio-Aid, Bucharest, Romania; 113 Department of Pediatrics, National Hospital, Abuja, Nigeria; 114 Department of International Public Health, University of Liverpool, Liverpool, UK; 115 National School of Public Health Institute of Health Carlos III, Madrid, Spain; 116 Privatpraxis, Heidelberg, Germany; 117 Vision and Eye Research Institute, Anglia Ruskin University, Cambridge, UK; 118 Institute for Health Metrics and Evaluation, Department of Health Metrics Sciences, University of Washington, Seattle, WA, USA; 119 Global Health Governance Programme, University of Edinburgh, Edinburgh, UK; 120 School of Dentistry, University of Leeds, Leeds, UK; 121 Department of Statistics and Econometrics Bucharest University of Economic Studies, Bucharest, Romania; 122 Department of Chemistry, University of Porto, Porto, Portugal; 123 Institute of Infection and Immunity, St George's University of London, London, UK; 124 University Medical Center Groningen, School of Economics and Business University of Groningen, Groningen, Netherlands; 125 WHO Collaborating Centre for Public Health Education and Training Imperial College London, London, UK; 126 Department of Primary Care and Public Health, Imperial College London, London, UK; 127 University College London Hospitals, London, UK; 128 Academic Public Health England, London, UK; 129 Clinical and Epidemiological Research in Primary Care (GICEAP), Maimonides Biomedical Research Institute of Cordoba (IMIBIC), Cordoba, Spain; 130 Clinical Epidemiology and Public Health Research Unit, Burlo Garofolo Institute for Maternal and Child Health, Trieste, Italy; 131 Department of Psychosocial Science University of Bergen, Bergen, Norway; 132 Department of Biomedical and Neuromotor Sciences, University of Bologna, Bologna, Italy; 133 Swiss Research Institute for Public Health and Addiction University of Zürich, Zurich, Switzerland; 134 Interdisciplinary Centre Psychopathology and Emotion regulation (ICPE) University of Groningen, Groningen, Netherlands; 135 Department of Nursing, Debre Berhan University, Debre Berhan, Ethiopia; 136 Finnish Institute of Occupational Health, Helsinki, Finland; 137 London Institute for Healthcare Engineering, King's College London, London, UK; 138 Division of Psychiatry and Applied Psychology, University of Nottingham, Nottingham, UK; 139 Department of Psychology, Reykjavik University, Reykjavik, Iceland; 140 Department of Health and Behavior Studies, Columbia University, New York, NY, USA; 141 Research Unit on Applied Molecular Biosciences (UCIBIO), University of Porto, Porto, Portugal; 142 Department of Biological Sciences, University of Porto, Porto, Portugal; 143 Surgery, "Sf. Pantelimon" Emergency Clinical Hospital Bucharest, Bucharest, Romania; 144 Faculty of Health and Public Administration, Semmelweis University, Budapest, Hungary; 145 Department of Medicine, University of Valencia, Valencia, Spain; 146 Carlos III Health Institute, Biomedical Research Networking Center for Mental Health Network (CiberSAM), Madrid, Spain; 147 Psychiatry and Medical Psychology Department, University of Lisbon, Lisbon, Portugal; 148 Child and Adolescent Mental Health Services (CAMHS), Hospital Garcia de Orta, Almada, Portugal; 149 Department of Pathology and Legal Medicine, University of São Paulo, Ribeirão Preto, Brazil; 150 Modestum LTD, London, UK; 151 UKK Institute, Tampere, Finland; 152 Faculty of Medicine and Health Technology, Tampere University, Tampere, Finland; 153 Department of Medical and Surgical Sciences and Advanced Technologies, University of Catania, Catania, Italy; 154 UCL Great Ormond Street Institute of Child Health, University College London, London, UK; 155 Demographic Change and Aging Research Area, Federal Institute for Population Research, Wiesbaden, Germany; 156 Institute of Health and Society, University of Oslo, Oslo, Norway; 157 Department of Neurology, Technical University of Munich, Munich, Germany; 158 Institute for Health Metrics and Evaluation, Department of Health Metrics Sciences, School of Medicine, University of Washington, Seattle, WA, USA; 159 School of Public Health (A J Ferrari PhD), The University of Queensland, Brisbane, QLD, Australia; 160 Department of Global Public Health, Karolinska Institute, Stockholm, Sweden; 161 Department of Psychiatry, Universidad Autónoma de Madrid (Autonomous University of Madrid), Madrid, Spain; 162 Polish Mothers' Memorial Hospital Research Institute, Lodz, Poland

**Keywords:** Young people, Mental health, Mental disorders, Self-harm, Substance use, Europe

## Abstract

**Background:**

Mental health is a public health issue for European young people, with great heterogeneity in resource allocation. Representative population-based studies are needed. The Global Burden of Disease (GBD) Study 2019 provides internationally comparable information on trends in the health status of populations and changes in the leading causes of disease burden over time.

**Methods:**

Prevalence, incidence, Years Lived with Disability (YLDs) and Years of Life Lost (YLLs) from mental disorders (MDs), substance use disorders (SUDs) and self-harm were estimated for young people aged 10-24 years in 31 European countries. Rates per 100,000 population, percentage changes in 1990-2019, 95% Uncertainty Intervals (UIs), and correlations with Sociodemographic Index (SDI), were estimated.

**Findings:**

In 2019, rates per 100,000 population were 16,983 (95% UI 12,823 – 21,630) for MDs, 3,891 (3,020 - 4,905) for SUDs, and 89·1 (63·8 - 123·1) for self-harm. In terms of disability, anxiety contributed to 647·3 (432–912·3) YLDs, while in terms of premature death, self-harm contributed to 319·6 (248·9–412·8) YLLs, per 100,000 population. Over the 30 years studied, YLDs increased in eating disorders (14·9%;9·4-20·1) and drug use disorders (16·9%;8·9-26·3), and decreased in idiopathic developmental intellectual disability (–29·1%;23·8-38·5). YLLs decreased in self-harm (–27·9%;38·3-18·7). Variations were found by sex, age-group and country. The burden of SUDs and self-harm was higher in countries with lower SDI, MDs were associated with SUDs.

**Interpretation:**

Mental health conditions represent an important burden among young people living in Europe. National policies should strengthen mental health, with a specific focus on young people.

**Funding:**

The Bill and Melinda Gates Foundation

**Research in context**

**Evidence before this study**

The Global Burden of Disease Study (GBD) 2019 estimates on prevalence, incidence, mortality, Years Lived with Disability (YLDs), Years of Life Lost (YLLs) and disability-adjusted life-years (DALYs) due to 369 diseases and injuries for 204 countries and territories became available in 2020. To complement GBD data, PubMed and Web of Science were searched for published papers on mental disorders in Europe and Google for gray literature in the public domain, as well as references in these papers and reports by November 2021, using the search terms “anxiety”, “alcohol use”, “attention-deficit disorder with hyperactivity”, “autism spectrum disorders”, “bipolar”, “burden”, “conduct disorder”, “depressive”,”depression”, “drug use”, “eating disorders”, ‘’epidemiology’’, “Europe”, “intellectual disability”, “mental disorders”, “mental health”, “prevalence”, “schizophrenia”, “self-harm”, “substance use”, “suicide”, “suicide attempt” and “trends”, without language or publication date restrictions. This literature was used as source of information for the references quoted in the study. Moreover, GBD 2019 estimates for mental disorders (MDs), substance use disorders (SUDs) and self-harm in 31 European countries (the 28 European Union (EU) countries [United Kingdom was still part of EU], plus Iceland, Norway, and Switzerland), sorted by country, sex and age groups (10-14, 15-19 and 20-24) were derived from 528 references. Earlier GBD studies have described mental health at a global level, and several national studies have described mental health in European countries. It is well recognized that mental health problems have increased in younger age groups. There was no previous comprehensive account of mental disorders in Europe, showing the development over time in different age groups, and in different parts of Europe. Self-harm has previously been analysed as an ‘injury’, and is included here as a mental disorder.

**Added value of this study**

This report describes the burden of mental conditions (i.e. MDs, SUDs and self-harm) in young people living in Europe, covering a 30-year period during which Europe faced profound political, social and demographic changes.It provides an overview of data sources used for epidemiological analyses. It describes variations in the prevalence of MDs and SUDs between 31 European countries, showing a lower burden of MDs in central and eastern Europe. Overall, the greatest burden is due to anxiety and depression. The report also describes changes in burden since 1990, with an increased burden due to disability from eating disorders and drug use disorders, and a decrease of idiopathic developmental intellectual disability, and alcohol use disorders. The burden of self-harm also decreased. The burden of SUDs and self-harm is higher in countries with lower development status as measured by the sociodemographic index (SDI). MDs are positively associated with SUDs.

**Implications of all the available evidence**

Mental health conditions in Europe represented a major health burden for younger people in the period 1990 to 2019, in terms of both disability and premature deaths. Given that these conditions often predict same or worse conditions in adulthood, and given that the estimated direct and indirect costs of these disorders are higher than those of chronic somatic diseases, our findings emphasise the need for policies to strengthen mental health in future years, with a specific focus on young people. The reported estimates of the burden and changes over time may be used by stakeholders to inform health planning. They also serve as an important point of reference when the full public health impact of the COVID-19 pandemic is assessed, in particularly in terms of the burden the pandemic has had on the mental health of young people in Europe.

Alt-text: Unlabelled box

## Introduction

Mental health conditions (i.e. mental disorders (MDs), substance use disorders (SUDs) and self-harm behaviours) are important causes of disease burden among young people in high-income countries,^1^^,^^2^ with conduct disorder, depression and anxiety disorder ranking among the top ten causes of years lived with disability (YLDs).^1^^,^^3^ The importance of mental health as a public health issue among young people is not reflected in the allocated resources,^4^ and many mental health conditions remain undetected and unmanaged for a long time.^5^ European countries show a high heterogeneity in resource allocation for child and adolescent mental health services (CAMHS).^6^ Further, only 70% of European countries have an official national adolescent mental health policy.^6^

Mental health receives in general limited research investment and political support in comparison with other non-communicable diseases (NCDs),^7^ and the need for a greater investment in mental care and a more equal distribution of funding across European Union (EU) countries has been highlighted.^8^ Further, there is a need for representative population-based studies using defined diagnostic criteria and standardized methods to assess MDs among European young people. Such studies are crucial to determine the true magnitude of the problem as well as to identify risk and protective factors to inform early interventions aimed at preventing co-morbidity and consequent long-term disability.^4^^,^^6^^,^^9^ Finally, countries in EU and Schengen area differ in terms of socio-economic development. Studies have shown higher burden of mental health conditions in high income countries than in middle and low income countries.^10^ However, as all these countries in Europe are classified as high income countries, the sociodemographic index (SDI) may be a better measure used to compare levels of disease burden by socioeconomic development in this region.

The Global Burden of Disease (GBD) Study aims to provide information on global trends in the health status of populations and changes in the leading causes of disease burden over time by assessing prevalence, incidence, premature deaths and non-fatal health health loss or disability.^3^ GBD allows for a comparison of cause-specific disease burden over time and by country through the standardisation of data management and methods.

Since an increasing prevalence of mental conditions (i.e. MDs, alcohol and substance use disorders [SUDs] and self-harm) in young people is observed across several European countries,^11^ a deeper understanding of the disease burden associated with these disorders is necessary to inform future health planning for different countries’ health systems. Although the hypothesis of a rise of MDs related to the ongoing COVID-19 pandemic has not yet been sufficiently investigated, it has been claimed that an increase in mental distress may result in a rise of mental conditions over the next years.^12^ Although it is not clear to what extent data and methodology available in the GBD will be able to quantify the burden of mental conditions due to the COVID-19 outbreak,^13^ a pre-pandemic baseline on mental conditions among young people in Europe may be a useful point of reference when the full public health impact of the pandemic is assessed.

The aims of this study are: 1) to describe the prevalence, incidence, YLDs and YLLs of different MDs, SUDs and self-harm in males and females aged 10–14, 15-19 and 20-24 years, from 1990 to 2019 among 31 European countries; 2) to describe trends in the prevalence and incidence of these disorders across European countries over this 30-year period;and 3) to correlate the prevalence and incidence of these disorders with the SDI of each European country.

This manuscript was produced as part of the GBD Collaborator Network and in accordance with the GBD Protocol.

## Methods

The Global Burden of Disease Study produces annual estimates on prevalence, incidence and mortality for 369 diseases and injuries. Each update incorporates new data and methodological improvements to provide stakeholders with the most up-to-date information for resource allocation decisions and are compliant with the Guidelines for Accurate and Transparent Health Estimates Reporting.^14^

The present study employed estimates from GBD 2019, which are available on the Global Health Data Exchange (GHDx).^15^ These estimates supersede those from previous rounds of GBD, since the estimates for the whole time series are updated on the basis of addition of new data and change in methods, where appropriate, at each iteration of the GBD study.^3^ Methods for the generation of GBD 2019 estimates are described in detail elsewhere,^3^ while the methodology for estimating the burden due to mental health conditions is briefly summarised here.

We provided results from 1990 to 2019 for 31 European countries: the 28 EU countries (the UK was still part of EU), plus Iceland, Norway and Switzerland, as part of Schengen area. The catchment population comprised young people between 10 and 24 years old,^16^ with a total number of 85 million subjects in 2019.

### Data sources

The estimates were based on data on incidence and prevalence identified through systematic searches of published and unpublished documents, survey microdata, administrative records of health encounters, registries, and disease surveillance system that are catalogued in the Global Health Data Exchange website (http://ghdx.healthdata.org). We summarized these data sources for the 31 countries of interest, related to MDs, SUDs and self-harm in the 10-24 age range, in Appendix (Overview on data coverage).

### GBD measures

We included the following measures of disease burden: prevalence (MDs and SUDs), incidence (self-harm,) YLDs, and YLLs. YLDs are years lived with disability (in which the disability equates to a fraction of a year lived in full health) and are the product of the prevalence and the disability weight of that condition. YLLs are years of life lost due to premature death, calculated as the difference between the corresponding standard life expectancy for that person's age and sex, and the age of actual death. Disability-adjusted life years (DALYs) are the sum of YLDs and YLLs. DALYs were used only to provide the fraction of YLDs and YLLs for each disorder.

Prevalence was derived from estimates of point prevalence for all MDs and SUDs, with the exception of bipolar disorders, where one-year prevalence was applied.^17^ We used prevalence estimates for all conditions which usually last more than six months. This involved also SUDs, even if a small degree of them also contributed also to premature deaths. We used incidence estimates for self-harm, since the great majority of the burden due to self-harm was represented by YLLs due to fatal self-harm.^18^

### Estimation of prevalence and incidence

Prevalence and incidence were modelled using DisMod-MR 2.1, a Bayesian meta-regression tool. Epidemiological data from different sources were pooled by DisMod-MR 2.1 with the goal of producing internally consistent estimates of prevalence, incidence, remission, and excess mortality by age, sex, location, and year.

### Estimation of severity

Proportions of severity were calculated to reflect the different levels of disability, or sequelae, associated with a determinate disorder, eg, mild, moderate, and severe presentations. Severity proportions, as shown elsewhere,^10^ were applied to the total prevalent cases estimated by DisMod-MR 2.1 to obtain prevalence estimates for each level of severity.

### Disability-weights

As described in detail in other studies based on GBD 2019,^3^^,^^19^ disability weights by condition were applied to estimate YLDs. These have been calculated through a series of severity splits, which definie the sequelae of a health condition as asymptomatic, mild, moderate, and severe. Disability weights derived from different international surveys, where a scale ranging from perfect health (0) to death (1) was used, adding also population health equivalence questions that compared the lifesaving benefits and the prevention programmes for several health states. The analysis of the surveys served for the relative position of health states to each other, while the population health equivalence questions were used to assess those relative positions as values on a scale ranging from 0 to 1. More information on the sequela-specific health state descriptions and on the disability weights analysis are described elsewhere.^10^

### Adjustment for comorbidity

A simulation method based on simulated populations of individuals by location, age, sex, and year, was used to adjust for comorbidity, since the burden attributable to each cause in GBD was estimated separately. Individuals in each population were exposed to the independent probability of having a combination of different sequelae in GBD 2019. A comorbidity correction was then used to estimate the difference between the average disability weight of individuals experiencing one sequela and the multiplicatively combined disability weights of those experiencing more sequelae. Specific YLDs per location, age, sex, and year applied the average comorbidity correction calculated for each sequela.^10^

### Uncertainty intervals

Uncertainty intervals (UIs) were used to describe the point estimates of uncertainty from model specification, stochastic variation, and measurement bias. UIs are based on 1000 draws from the posterior distribution of estimates. The point estimate is defined by the mean of the draws, while the the 95% UIs is represented by the 2·5^th^ and 97·5^th^ percentiles ranked estimates from the drawns.

### GBD causes hierarchy

In GBD 2019, diseases and injuries and causes of death, were aggregated in three Level 1 causes (communicable, maternal, neonatal, and nutritional conditions; NCDs; and injuries), 22 Level 2 causes, 174 Level 3 causes, and 301 Level 4 causes.^3^

In this study, we included Level 2 (MDs and SUDs) and Level 3 causes, as follows:

• MDs: anxiety disorders, attention deficit/hyperactivity disorder (ADHD), autism spectrum disorder (ASD), bipolar disorder, conduct disorder, depressive disorders, eating disorders, idiopathic developmental intellectual disability (IDID), schizophrenia, other mental disorders;
• SUDs: alcohol use disorders, drug use disorders;
• Self-harm.

Only for MDs, we also aggregate disorders as follows, to describing prevalence rates among the 31 countries of interest:

a) Common MDs^20^: depressive and anxiety disorders;
b) Severe MDs^21^: schizophrenia and bipolar disorders;
c) “Other” MDs: Eating disorders, ASD, ADHD, Conduct disorders, IDID, Other mental disorders.

Diagnostic and Statistical Manual of Mental Disorders (DSM-IV-TR) or the International Classification of Diseases – Tenth revision (ICD-10) criteria were used for definition of cases,^19^ as they were used by the majority of mental health surveys included in the Appendix.

### Socio-demographic Index

the. SDI is a composite indicator of development status, built as the geometric mean of 0 to 1 indices of total fertility rate in women younger than 25 years, mean education for the population aged 15 years and older, and lag-distributed income per capita.^3^ We used the SDI for each of the 31 countries of this study.

### Data presentation

For each country, cause and year, we report count, age rates per 100,000 population for age subgroups, and percentage changes from 1990 to 2019 for estimates of prevalence, incidence, YLDs, and YLLs, with 95% UIs,^3^. The Institute for Health Metrics and Evaluation (IHME) provided aggregated estimates for all 31 countries combined, since the standard GBD aggregate estimates are for the EU, and exclude the other Schengen area countries (i.e. Iceland, Norway and Switzerland) .. Results are presented by sex and age subgroups (10-14; 15-19 and 20-24 years). YLLs were calculated only for self-harm, eating disorders, alcohol use disorders and substance use disorders since these are the only causes considered causes of death in the WHO/ICD-system (https://www.who.int/standards/classifications).

We also reported the percentages of YLDs and YLLs of MDs, SUDs and self-harm in the 10-24 age groups compared to the all-causes GBD in the 31 European countries.

In addition, we performed Spearman rank-correlations to study the relation between SDI and prevalence rates of MDs and SUDs, and incidence rates of self-harm. We set P-value <0·05 as the threshold of statistical significance. These analyses were conducted with Stata/BE 17.0 (StataCorp LLC, College Station, USA).

### Role of the funding source

The funder of the study had no role in study design, data collection, analysis and interpretation, or writing of the report. The corresponding author had full access to all the data in the study and had final responsibility to submit for publication.

## Results

### Prevalence of MDs, SUDs and incidence of self-harm

In 2019, there were 13·6 million (95% UI 15·5–11·9) young people in the 31 European countries with MDs, 3·2 (2·6–4·0) million with SUDs, and 75,770 (59,091–95,206) self-harmed. In terms of rates per 100,000 population, they were 16,983 (12,823 – 21,630), 3,891 (3,020 - 4,905), and 89·1 (63·8 - 123·1), respectively.

As shown in Table 1, the most prevalent condition in 2019 was anxiety disorders, in terms of both count (5·6 million;3·8-7·7 million cases) and rates (6·6; 4·9-8·4 cases per 100,000 population), with an increase of 4·7% (1·1–8·6) from 1990. A decrease of 7·5% (2·9–13·6) was found for alcohol use disorders from 1990. Self-harm decreased by almost 40% (36·3–42·6).

**Table 1:** Prevalence, Incidence, Years Lived with Disability (YLDs) and Years’ Life Lost (YLLs) for mental disorders, substance abuse and self-harm in European Union, Iceland, Norway and Switzerland, years 1990-2019, both sexes, age 10-24, counts, rates and percentage change over time.

	Counts (95% Uncertainty Intervals)			Rates per 100,000 population (95% Uncertainty Intervals)		
		1990	2019	1990	2019	Change %
**Mental disorders**						
Anxiety disorders	Prevalence	6,659,301 (4,624,216; 9,783,510)	5,582,658 (3,844,784; 7,748,341)	6,155 (4,537; 8,109)	6,567 (4,973; 8,360)	4·7 (1·1; 8·6)
	YLDs	655,912 (399,848; 1,007,683)	550,220 (333,975; 845,550)	606·2 (392·3; 880·7)	647·3 (432; 912·3)	4·8 (0·9; 9)
ADHD	Prevalence	2,660,206 (1,820,396; 3,761,625)	2,256,716 (1,526,534; 3,243,148)	2,459 (1,786; 3,288)	2,655 (1,975; 3,499)	5·8 (1·3; 10·8)
	YLDs	32,448 (17364; 57,009)	27,541 (14,629; 48,869)	30 (17·0; 49·8)	32·4 (18·9; 52·7)	5·9 (0·4; 11·7)
ASD	Prevalence	612,121 (508,524; 731,129)	506,530 (421,652; 602,444)	566 (499; 639)	596 (545; 650)	3·4 (2·2; 4·6)
	YLDs	94,732 (61,013; 138,483)	78,390 (50,592; 114,642)	87·6 (59·9; 121)	92·2 (65·4;123·7)	3·4 (0·6; 6·1)
Bipolar disorder	Prevalence	883,032 (613,263; 1,212,260)	718,481 (500,256; 831,600)	816 (602; 1,060)	845 (647; 1,061)	1·6 (0·8; 4·5)
	YLDs	196,567 (103,423; 323,729)	159,975 (84,636; 264,969)	181·7 (101·5; 282·9)	188·2 (109·5; 285·9)	1·7 (-1·9; 5·4)
Conduct disorders	Prevalence	1,989,600 (1,381,925; 2,709,809)	1,641,386 (1,140,564; 2,236,415)	1,839 (1,356; 2,368)	1,931 (1,475; 2,413)	3·0 (2·0; 4·1)
	YLDs	241,387 (130,636; 393,196)	199,195 (107,979; 324,790)	223·1 (128·2; 343·7)	234·3 (139·7; 350·4)	3·1 (0·9; 5·3)
Depressive disorders	Prevalence	3,382,030 (2,530,390; 4,388,585)	2,619,276 (1,883,469; 3,512,140)	3,126 (2,482; 3,836)	3,081 (2,436; 3,789)	-3·4 (-2·4; 8·8)
	YLDs	626,008 (386,014; 953,873)	484,289 (289,832; 756,723)	578·6 (378·7; 833·7)	569·7 (374·9; 816·4)	-3·6 (-9·7; 3)
Eating disorders	Prevalence	646,946 (418,130; 961,896)	595,413 (380,504; 894,360)	598 (410; 841)	700 (492; 965)	14·9 (9·4; 20)
	YLDs	138,467 (75,902; 229,997)	127,494 (69,672; 213,850)	128·0 (74·5; 201)	150·0 (90·1; 230·7)	14·9 (9·4; 20·1)
	YLLs	1031 (670; 1617)	1075 (616; 1848)	1·0 (0·6; 1·6)	1·3 (0·7; 2·4)	31·2 (2·0; 63·3)
IDID	Prevalence	789,007 (365,767; 1,212,592)	438,934 (165,902; 713,992)	729 (359; 1,060)	516 (215; 770)	-31·5 (-43·3; -25·6)
	YLDs	35,359 (4,814; 61,565)	20,263 (7,379; 36,522)	32·7 (14·5; 53·8)	23·8 (9·5; 39·4)	-29·1 (–38·5; 23·8)
Schizophrenia	Prevalence	100,919 (65,486; 148,235)	76,806 (49,490; 113,744)	93 (64; 130)	90 (64; 123)	-5 (-7·47; -2·66)
	YLDs	67,121 (37,773;107,565)	40,977 (21,084; 67,728)	62 (37·1; 94)	60·3 (37·3; 89·3)	-4·7 (-11·6; 1·5)
Other mental disorders	Prevalence	674,330 (424,980; 958,463)	531,531 (335,250; 754,731)	623 (417; 838)	625 (434; 814)	-1·5 (-1·9; -1·1)
	YLDs	51,989 (26,609; 85,710)	51,223 (28,859; 82,807)	48·1 (26·1; 74·9)	48·2 (27·3; 73·1)	-1·5 (-6·7; 3·7)
**Substance use disorders**						
Alcohol use disorders	Prevalence	1,993,616 (1,261,281; 2,907,7949	1,480,042 (912,167; 2,196,529)	1,843 (1,237; 2,541)	1,741 (1,180; 2,370)	-7·5 (-13·6; -2·9)
	YLDs	204,105 (113,367; 335,338)	151,473 (82,495; 254,273)	188·6 (111·2; 293·1)	178·2 (106·7; 274·3)	-7·6 (-13·8; -2·4)
	YLLs	13,700 (11,175; 16676)	7891 (5929; 10,313)	12·7 (9·8; 16·4)	9·3 (6·4; 13·3)	-37·5 (-41·3; -32·9)
Drug use disorders	Prevalence	2,309,892 (1,729,613; 3,077,292)	1,827,533 (1,422,606; 2,350,350)	2,135 2,690; 1,697	2,150 2,536; 1,840	-0·9 (-7·5; 5·6)
	YLDs	229,235 (148,067; 329,472)	214,408 (138,831; 307,728)	211·9 (145·3; 288)	252·2 (179·6; 332)	16·9 (8·9; 26·3)
	YLLs	83,639 (68028; 102,329)	56,961 (42,081; 76,209)	77·3 (59·5; 100·4)	67·0 (45·4; 98·6)	-14·8 (-24·2; -2·8)
**Self-harm**						
	Incidence	124,715 (100,589; 152,872)	75,770 (59,091; 95,206)	115·3 (87·9; 150·0)	89·1 (63·8; 123·1)	-39·3 (-42·6; -36·3)
	YLDs	4,617 (3,017; 6,591)	2,621 (1,696; 3,756)	4·3 (3·0; 5·8)	3·1 (2·2; 4·1)	-29·1 (-32·0; -26·0)
	YLLs	543,528 (491816; 598905)	271,675 (230,719; 319,118)	502·4 (429·9; 587·6)	319·6 (248·9; 412·8)	-27·9 (-38·3; -18·7)

YLDs, years lived with a disability; ADHD, Attention deficit/hyperactivity disorder; ASD Autism spectrum disorders; IDID Idiopathic developmental intellectual disability.

Substantial differences in prevalence rates were observed in relation to sex. Anxiety, depressive and eating disorders were more prevalent among females than males, while the opposite was observed for ADHD, ASD, conduct disorders and“other MDs”, as well as alcohol and drug use disorders (Supplement Tables 1). In terms of age-groups, anxiety disorders, ADHD and conduct disorderswere the most prevalent disorders among males aged 10 to 14 years, while anxiety disorders had the highest prevalence among females in this age group. Anxiety disorders remained the most prevalent disorder both among aged 15 to 19 and 20 to 24 years and among males aged 15 to 19 . The most prevalent disorders among males aged 20 to 24 years were anxiety, alcohol and drug use (Supplement tables 2, 3 and 4).

The largest changes in prevalence over time from 1990 were observed for eating disorders (+14·9%; 9·4–20), and IDID (–31·5%; 25·6–43·3) in both sexes combined (Table 1). Similar tendency in change of prevalence was observed when stratified by sex and age-groups (Figure 3, Supplement tables 2, 3 and 4).

### Prevalence of MDs, SUDs and incidence of self-harm by country

**Figure 1:**
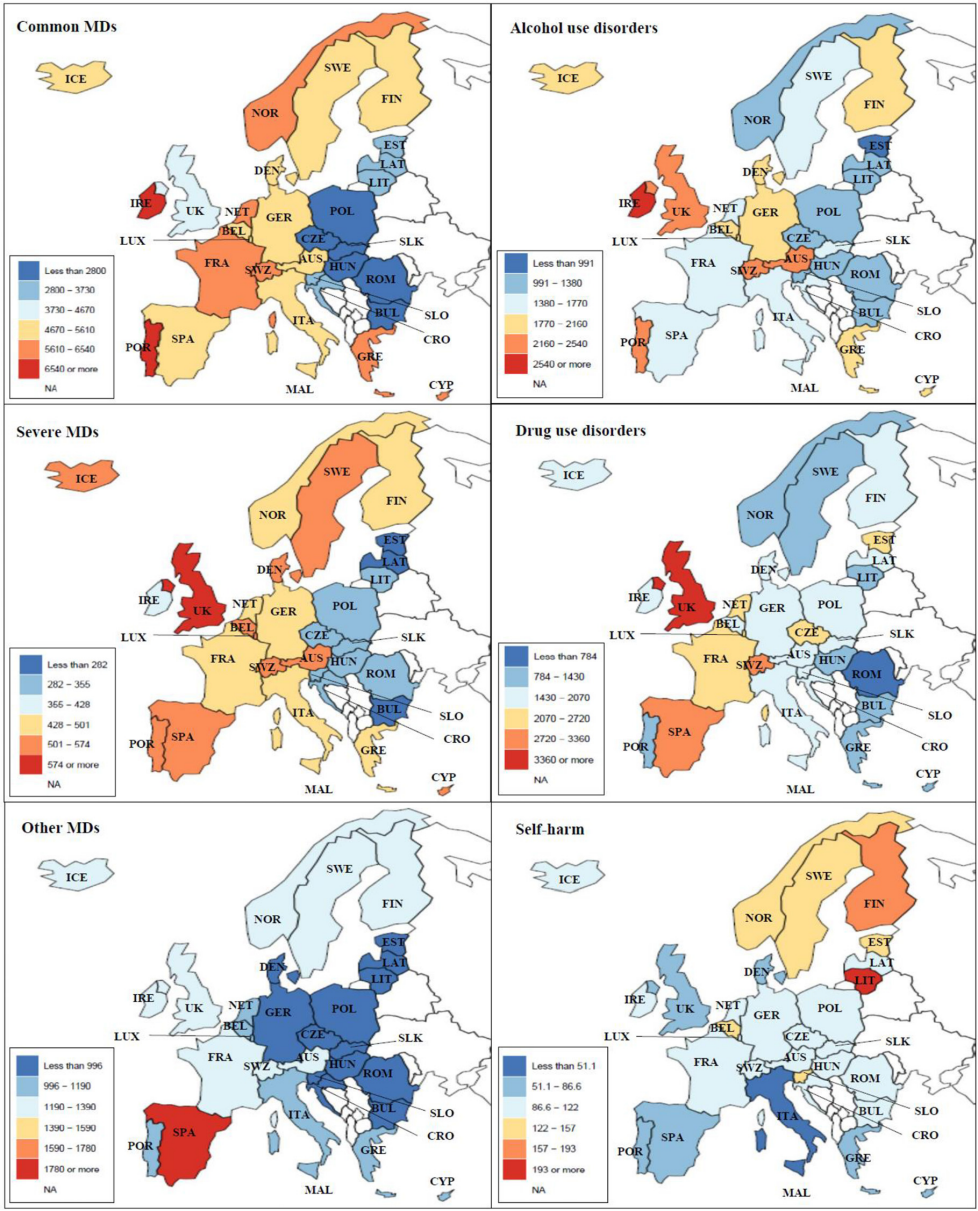
Prevalence per 100,000 population aged 10-24 years of common, severe and other mental disorders (MDs), alcohol and drug use disorders, and incidence rate of self-harm in 31 European countries, both sexes, age 10-24, year 2019. Common MDs: anxiety and depressive disorders; Severe MDs: schizophrenia and bipolar disorder; Other MDs: eating disorders, attention deficit/hyperactivity disorder, autism spectrum disorder, conduct disorders, idiopathic developmental intellectual disability, other mental disorders.

Central and eastern Europe (i.e. Bulgaria, Croatia, Czech, Estonia, Hungary, Latvia, Lithuania, Poland, Romania. Slovakia and Slovenia) were generally characterized by lower prevalence rates of common and severe MDs and alcohol use disorders compared to the rest of Europe, while rates were more heterogeneous for the other conditions (Figure 1). During the 30-year period, higher MD rates were observed in Portugal and in Spain (around 20,000 per 100,000), while higher SUD rates were observed in Switzerland and UK (5500 or more per 100,000) (Supplement table 5 and 6). As summarized in Supplement table 7, the highest incidence rates of self-harm were in Lithuania (from 227·8 per 100,000 in 1990 to 207·1 in 2019) and in Finland (from 266·2 per 100,000 in 1990 to 185·8 in 2019).

### YLDs of MDs, SUDs and self-harm

MDs were the leading cause of YLDs in all the 31 countries in 2019 (Supplement table 8), with anxiety and depressive disorders ranking between the first and the fourth position in each country (Supplement table 9).

In 2019, anxiety and depressive disorders were the leading causes of YLDs among mental health conditions, contributing to a total count of 550,220 (333,975 - 845,550) and 484,289 (289,832-756,723) YLDs, respectively. The greatest increases from 1990 were observed for eating disorders (+15%; 9·4–20·1), and for drug use (+17%; 8·9–26·3), while IDID (–29%; 23·8–38·5) and self-harm (-29·1; 32·0 - 26·0) greatly decreased (Table 1).

**Figure 2:**
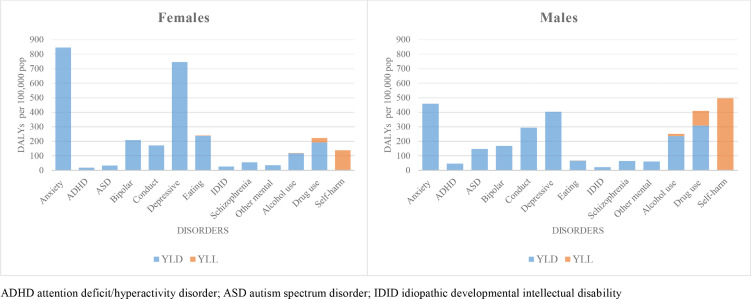
Disability Adjusted Life Years (DALYs) divided in Years Lived with Disability (YLDs) and Years of Life Lost (YLLs) for mental disorders, substance use disorders and self-harm in 31 European countries, females and males, age 10-24, year 2019.

When comparing sexes, YLD rates in males were 4·5 times higher yhan in females for ASD and more than twice as high for ADHD while YLD rates in females were 3·5 times higher than in males for eating disorders and nearly twice as high for anxiety and depressive disorders. YLD rates for alcohol and drug use disorders were more than 1·5 times higher in males compared to females (Figure 2, Supplement table 1).

With regard to age groups, the highest YLD rates in 2019 in females aged 10 to 14 years were for anxiety disorders, and in males were for conduct and anxiety disorders. In the older age-groups, anxiety and depressive disorders had the highest level of YLD rates in both sexes, although they were higher among females. YLDs for alcohol and drug use disorders were quite low until 14 years old, and due to the age of onset used to model prevalence, they increased substantially from age 15, and had equivalently high levels of YLD rates of anxiety and depressive disorders in males aged 20 to 24 years (Supplement Tables 2, 3 and 4).

**Figure 3:**
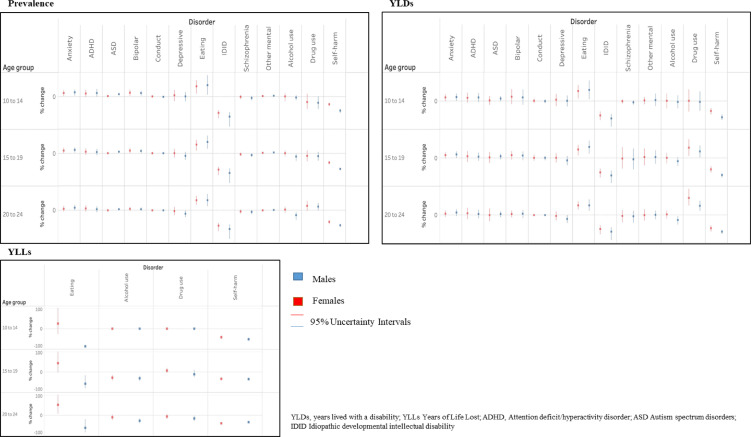
Prevalence for mental disorders, substance use disorders, and incidence for self-harm in European Union, Iceland, Norway and Switzerland, years 1990-2019, males and females, age 10-14, 15-19, 20-24 percentage change over time.

The largest increase in YLD rates from 1990 were found for eating disorders in both sexes and in all age groups, and for drug disorders from 15 years old. YLD rates due to IDID and self-harm decreased significantly in both sexes and in all age groups (Figure 3).

### YLLs of eating disorders, SUDs and self-harm

Self-harm ranked as the first to the third cause of YLLs in all 31 countries in 2019 (Supplement table 10).

Among the mental health conditions, self-harm was the main contributor to YLL rates per 100,000 population (319·6 (248·9–412·8) in 2019) (Table 1), with rates almost four-times higher in males compared to females in 2019 (Figure 2; Supplement table 1). Drug use disorders were the second highest contributor of YLLs, especially in males (Table 1; Supplement table 1).

With regard to age groups, the highest YLL rates per 100,000 population in 2019 were found in males aged 20-24 years for self-harm (937·9; 863·5 - 1,019·3), and for drug use disorders (228·2; 184·3- 281·2)(Supplement Table 4).

YLL rates decreased from 1990 in all conditions considered (Table 1), except eating disorders in females, which increased by 42·6% (9·7-80·2) (Supplement table 1).

### Correlation between SDI and prevalence rates of MDs, SUDs and self-harm, and between prevalence rates of MDs and SUDs

All 31 countries were included in high or high-middle SDI global quintiles. SDI varied from 0·59 (Portugal) to 0·82 (Norway) in 1990, and from 0·72 (Portugal) to 0·93 (Switzerland) in 2019. As shown in Table 2, SUDs and self-harm showed a significant correlation with growing SDI in 1990 which was still significant in 2019. Prevalence rates of MDs were significantly associated with those of SUDs in both years.

**Table 2:** Spearman rank-correlation (coefficients and relative P-values) among prevalence of mental disorders, substance use disorders, incidence of self-harm and Social Demographic Index in the European Union, Iceland, Norway and Switzerland, years 1990-2019, both sexes, age 10-24. Significant results are highlighted in bold.

	1990			2019		
	Spearman rank-correlation coefficient (P-value)			Spearman rank-correlation coefficient (P-value)		
	MDs	SUDs	Self-harm	MDs	SUDs	Self-harm
MDs	1·0			1·0		
SUDs	**0**·**54 (0·001)**	1·0		**0·47 (0·008)**	1·0	
Self-harm	- 0·04 (0·80)	- 0·03 (0·86)	1·0	- 0·30 (0·10)	- 0·13 (0·47)	1·0
SDI	0·16 (0·38)	**0·41 (0·02)**	**0·37 (0·04)**	0·25 (0·17)	**0·35 (0·05)**	**0·39 (0·03)**

MDs Mental disorders; SUDs Substance use disorders; SDI Socio Demographic Index.

Among countries with higher SDI, UK showed the highest level of SUDs, while Lithuania showed the greatest incidence of self-harm (Supplement figure 1 and 2). Regarding the correlation between SUDs and MDs, Central and Eastern Europe showed lower levels of MDs and somewhat of SUDs, while Spain was characterized by higher levels of both (Supplement Figure 3).

## Discussion

Almost 17 million young people (19·8%) in these 31 European countries had a mental or substance use disorder in 2019. In total, mental conditions contributed to more than 1 million YLDs in 2019, and were the leading cause of disability among young people in most European countries. Prevalence of MDs increased from 1990 to 2019, while incidence of self-harm decreased by more than 20% in both sexes. Among MDs, the greatest increase was observed for eating disorders (15%), ADHD (6%) and anxiety (5%).

In the period 1990 to 2019, YLD rates due to eating disorders and drug use disorders increased in both sexes from age 10 and 15, respectively, while YLDs rates due to IDID decreased in both sexes and all age-groups. There were considerable sex differences: male/female ratio for ASD was 4·5:1, and almost 2:1 for ADHD and SUDs; conversely it was 1:3·5 for eating disorders and almost 1:2 for anxiety and depressive disorders.

Self-harm has been a leading cause of YLLs among yound people in most European countries since 1990, although a decrease of almost 30% was observed in the 30-year period.

### Mental disorders

Previous findings at the global level indicated a heavy burden of MDs and subsequent disability in the young population.^1^^,^^2^^,^^5^^,^^22^ Given the high prevalence of common MDs and their burden in terms of disability in adulthood,^9^ these disorders are a key target for health policy and programme planning in the European agenda.^11^ There was a particularly strong increase in the occurrence of eating disorders, both in males and females across all age groups. Although it is uncertain to what extent these increasing rates were due a true rise in prevalence rather than changes in diagnostic practices or improved detection, it has been suggested that many individuals suffering from eating disorders still remain untreated in Europe.^23^ ADHD showed a 6% increase in prevalence from 1990. Although this may also be indication of an improvement in the detection of ADHD cases, care for this disorder should be enhanced, given that more than 60% of children diagnosed with ADHD have symptoms as adults.^24^ We found a 30% decrease of IDID, in terms of both prevalence and disability, which reflects the decrease in occurrence of intellectual disability observed in children under 5 years from 1990 to 2016 in Europe, probably related to an improvement in early detection and interventions among prematurely-born children.^25^

Emerging evidence has shown that socio-economic deprivation is an important determinant of many mental disorders such as schizophrenia, anxiety and depression, substance use disorders, and self-harm.^11^^,^^26^ In our study Portugal, having the lowest SDI during the 30-year period, also had the highest burden of MDs, perhaps implying some influence of lower socioeconomic conditions on MDs. In contrast, Central and Eastern European countries, where socioeconomic conditions improved from 1990,^27^ were characterized by lower MDs rates during this period. However, we found no correlation between MDs and SDI at country level in the present study. European countries, however, are all characterised by high SDI, which may have hindered the identification of significant differences between countries. Strong correlations were found in studies based on specific geographical areas with important differences in SDI, with high-income countries generally characterised by a higher burden due to MDs, in particular depression and anxiety.^10^^,^^19^^,^^28^ Other explanations for the heterogeneity across countries may be linked to the high degree of variations in epidemiological data, as acknowledged in our overview on data coverage. Estimates for Central and Eastern Europe were based on a mean of only 2·5 publications per country, which may have led to an underestimation of MDs in these countries.

### Substance use disorders

Alcohol and drug use disorders represent a major burden on young people living in Europe, among the highest in the world.^2^ YLDs due to drug use disorders increased in both sexes from 15 years old. Cannabis use is widespread among European young people, along with the use of cocaine and ecstasy, leading to detrimental effects on their mental and physical health.^11^ Europe has high death rates caused by opioid dependence,^29^ which often starts during adolescence. Drug use was also responsible for almost 60,000 YLLs in 2019. Moreover, the burden attributable to SUDs represent an additional risk for other health problems later in life, such as injuries, self-harm, and somatic diseases. Although Central and Eastern Europe had the highest alcohol and drug attributable burdens^30^ a decreasing trend of alcohol use disorders in these countries was observed during the study period. This decline is in line with previous observations from European countries till 2014.^31^ It might be related to the implementation of evidence based public health policies, such as limiting the access to alcohol by raising the minimum legal age for drinking, increasing prices, or regulating advertisement in the media.^11^^,^^31^ However, there is still a high heterogeneity in policy measures,^32^ and countries with higher SDI have a greater burden.^30^

### Self-harm

Self-harm was the third leading cause of DALYs among people aged 10-24 years worldwide in 2019 ^3^ and it is among the five leading causes of young people's mortality in Europe.^2^^,^^33^ Many young people with suicidal behaviours are still not detected by health services,^34^ and only half of EU countries have agreements between school and health services to facilitate referrals to CAMHS and official guidelines for referring patients from primary care.^6^ However, we observed a drop in incidence and YLLs, in line with the one-third decrease in suicide rates between 2000 and 2017, previously described.^11^ This may reflect a positive effect of suicide prevention strategies or an improvement of health of the population ^33^, as well as an enhancement in help-seeking and service availability for adolescents. ^6^ ^34^ High differences among countries suggest that self-harm is due to a complex interplay of specific factors related to each country, including social, economic, and cultural factors, and a different distribution of MDs and SUDs.^33^

### Correlation between MDs and SUDs

It was somewhat expected to observe a significant correlation between MDs and SUDs in all 31 European countries. As previously highlighted,^5^^,^^11^^,^^18^^,^^35^^,^^36^ there is a strong correlation between MDs and SUDs in young people. A person-centred care approach to young people's needs might better manage the complex interactions of clinical and social factors underlying mental conditions, including substance use and self harm. Services for mental care for young people in Europe often do not match the epidemiological burden of these conditions,^6^ lack in specific tailored care pathways based on specific psychosocial needs, and lack in the connection with adult mental health services^37^ and social services.^32^

### Strengths and limitations

The main strength of this study is giving an extensive account of mental health conditions in young people living in Europe, covering 30 years during which Europe faced profound political, social and demographic changes. This is also the first study analysing the extent of the burden of these conditions in young people in Europe. Previous studies at global level did not analyse specifically these disorders in young people, or were limited to MDs,^3^ not including SUDs and self-harm.^10^ Additionally, it provides an overview on data sources used for epidemiological analyses. A limitation is the lack of relevant data in some countries, particularly in Central and Eastern Europe, even though all these 31 countries are among those with the largest amount of data compared to other areas of the world.^9^^,^^38^

Other limitations should also be acknowledged. First, the study suffers from the general limitations of GBD studies, such as uncertainties in the determination and classification of non-fatal disorders, deriving from different data sources, and the uncertainty of some estimates, reflected by the width of the 95% UIs for certain disorders. Second, an underestimation of the true effect of mental health conditions may be due to the fact that self-harm is coded in GBD under injuries, while it is largely linked to MDs and SUDs. We included self-harm in our analyses, although it was not possible to calculate the attributable burden of suicide, which could have increased the overall burden of MDs and SUDs.^18^^,^^39^ This calculation is underway by IHME, using GBD 2020 estimates.^10^ However, we provided the correlation of self-harm with MDs and SUDs in our analyses, without finding significant correlations. This lack of association is consistent with recent findings that the majority of young people who self-harm do not have a mental disorder.^40^ Third, estimates on personality disorders are included in the broad group of other MDs, due to limited informing prevalence estimates.^3^ However, it would be suitable to include them in future GBD estimates, given a lifetime prevalence of personality disorder in the EU of more than four million people.^9^ Finally, SDI was chosen as a summary measure of a geography's socio-demographic development, while more precise measures could be used, such as the Index of Multiple Deprivation (IMD), which includes several domains.^41^ However, IMD was not available for most of the countries of interest, and SDI is commonly used in GBD studies on large areas.^3^

## Conclusions

The burden of mental and drug use disorders slightly increased from 1990, while alcohol use disorders and especially self-harm decreased among young people in Europe. It is concerning that all these conditions still represent a major health burden, especially in terms of disability, but also in terms of premature deaths.^36^ Given that young people's mental disorders often predict the same or worse conditions in adulthood,^2^^,^^5^ and a shift towards health loss due to NCDs has been observed,^1^ our findings support the assertion that mental conditions should be considered a core health challenge of the 21^st^ century.^9^ Further, the estimated direct and indirect costs of these disorders are higher than those of chronic somatic diseases.^7^ There is a need to capture data more comprehensively to truly understand the impact of the COVID-19 pandemic on mental health in Europe,^11^^,^^12^ since this study demonstrated a possible underestimation of the actual burden of mental conditions, owing to the paucity of data in Central and Eastern European countries, and the GBD classification of self-harm. Given all these factors, national policies should provide evidence-based preventive initiatives and accessible treatment for mental health disorders in young people.^10^^,^^30^^,^^33^^,^^42^

## Declaration of interests

T W Bärnighausen reports Research grants from the European Union (Horizon 2020 and EIT Health), German Research Foundation (DFG), US National Institutes of Health, German Ministry of Education and Research, Alexander von Humboldt Foundation, Else-Kröner-Fresenius-Foundation, Wellcome Trust, Bill & Melinda Gates Foundation, KfW, UNAIDS, and WHO; consulting fees from KfW on the OSCAR initiative in Vietnam; participation on a Data Safety Monitoring Board or Advisory Board with NIH-funded study “Healthy Options” as Chair, Data Safety and Monitoring Board (DSMB) for the German National Committee on the “Future of Public Health Research and Education”, Chair of the scientific advisory board to the EDCTP Evaluation, Member of the UNAIDS Evaluation Expert Advisory Committee, National Institutes of Health Study Section Member on Population and Public Health Approaches to HIV/AIDS (PPAH), US National Academies of Sciences, Engineering, and Medicine's Committee for the “Evaluation of Human Resources for Health in the Republic of Rwanda under the President's Emergency Plan for AIDS Relief (PEPFAR)”, University of Pennsylvania Population Aging Research Center (PARC) External Advisory Board Member; leadership or fiduciary role in other board, society, committee or advocacy group, paid or unpaid as Co-chair of the Global Health Hub Germany, initiated by the German Ministry of Health); all outside the submitted work. J S Chandan reports grants or contracts from the National Institute of Health Research and has been awarded funds from the NIHR and the Youth Endowment Fund, outside the submitted work. J J Jozwiak reports payment or honoraria for lectures, presentations, speaker's bureaus, manuscript writing or educational events from Teva, Amgen, Synexus, Boehringer Ingelheim, ALAB Laboratories, and Zentiva, all as personal fees and outside the submitted work. S V Katikireddi reports support for the present manuscript form Medical Research Council and the Scottish Government Chief Scientist Office as funding to their institution. J H Kauppila reports grants or contracts from The Finnish Cancer Foundation, and Sigrid Juselius Foundation as payments made to their institution, outside the submitted work. M Kivimäki reports grants or contracts form Wellcome Trust, UK (221854/Z/20/Z), and the Medical Research Council, UK (MR/R024227/1, MR/S011676/1) as the PI of research funding for their university, outside the submitted work. G Logroscino reports honoraria for lectures from Amplifon, outside the submitted work. J A Louriero reports support for the present manuscript from Fundação para a Ciência e Técnologia (FCT) under the Scientific Employment Stimulus [CEECINST/00049/2018]. A-F A Mentis reports grants or contracts from ‘MilkSafe: A novel pipeline to enrich formula milk using omics technologies’, a research co-financed by the European Regional Development Fund of the European Union and Greek national funds through the Operational Program Competitiveness, Entrepreneurship and Innovation, under the call RESEARCH - CREATE - INNOVATE (project code: T2EDK-02222), as well as from ELIDEK (Hellenic Foundation for Research and Innovation, MIMS-860); stock or stock options in a family winery; all outside the submitted work. M J Postma reports stock or stock options from Health-Ecore and PAG, outside the submitted work. N Steel reports grants from Public Health England to their institution, outside the submitted work. R M Viner reports payment or honoraria for lectures, presentations, speakers bureaus, manuscript writing or educational events from Canadian Academy of Child & Adolescent Psychiatry for lecture on mental health aspects of COVID-19 pandemic; leadership or fiduciary role in other board, society, committee or advocacy group, paid or unpaid, as the President of Royal College of Paediatrics & Child Health, 2018-2021; all outside the submitted work.

## Appendix B Supplementary materials

Image, application 1

Image, application 2

Image, application 3
