# Supplementary material for: The burden of mental disorders, substance use disorders and self-harm among young people in Europe, 1990–2019: Findings from the Global Burden of Disease Study 2019
Source: Lancet Reg Health Eur. 2022 Apr 1;16:100341. doi: 10.1016/j.lanepe.2022.100341 (PMC8980870; doi:10.1016/j.lanepe.2022.100341)

**Caption for supplementary material:**

**Tables:**

- Supplement Table 1. Prevalence, Incidence, Years Lived with Disability (YLDs) and Years of Life Lost (YLLs), where available, for mental disorders, substance abuse and self-harm in European Union, Iceland, Norway and Switzerland, years 1990-2019, males and females, age 10-24, rates and percentage change over time
- Supplement Table 2: Prevalence, Incidence, Years Lived with Disability (YLDs) and Years of Life Lost (YLLs), where available, for mental disorders, substance abuse and self-harm in European Union, Iceland, Norway and Switzerland, years 1990-2019, males and females, age 10-14, rates and percentage change over time
- Supplement Table 3∙ Prevalence, Incidence, Years Lived with Disability (YLDs) and Years of Life Lost (YLLs), where available, for mental disorders, substance abuse and self-harm in European Union, Iceland, Norway and Switzerland, years 1990-2019, males and females, age 15-19, rates and percentage change over time
- Supplement Table 4: Prevalence, Incidence, Years Lived with Disability (YLDs) and Years of Life Lost (YLLs), where available, for mental disorders, substance abuse and self-harm in European Union, Iceland, Norway and Switzerland, years 1990-2019, males and females, age 20-24, rates and percentage change over time
- Supplement Table 5: Prevalence per 100,000 population aged 10-24 years of mental disorders in 31 European countries from 1990 to 2019
- Supplement Table 6: Prevalence per 100,000 population aged 10-24 years of substance use disorders in 31 European countries from 1990 to 2019
- Supplement Table 7: Incidence per 100,000 population aged 10-24 years of self-harm in 31 European countries from 1990 to 2019
- Supplement Table 8: Ranking of Years Lived with Disability (YLDs) for the first 22 all-causes of diseases at level 2 in European Union, Iceland, Norway and Switzerland, year 2019, both sexes, age 10-24
- Supplement Table 9: Ranking of Years Lived with Disability (YLDs) for the first 25 all-causes of diseases at level 3 in European Union, Iceland, Norway and Switzerland, year 2019, both sexes, age 10-24
- Supplement Table 10: Ranking of Years of Life Lost (YLLs) for the first 25 all-causes of diseases at level 3 in European Union, Iceland, Norway and Switzerland, year 2019, both sexes, age 10-24

**Figures:**

- Supplement Figure 1: Correlation between prevalence of substance use disorders (SUDs) and Socio Demographic Index (SDI), in 31 European countries, year 2019, both sexes, age 10-24


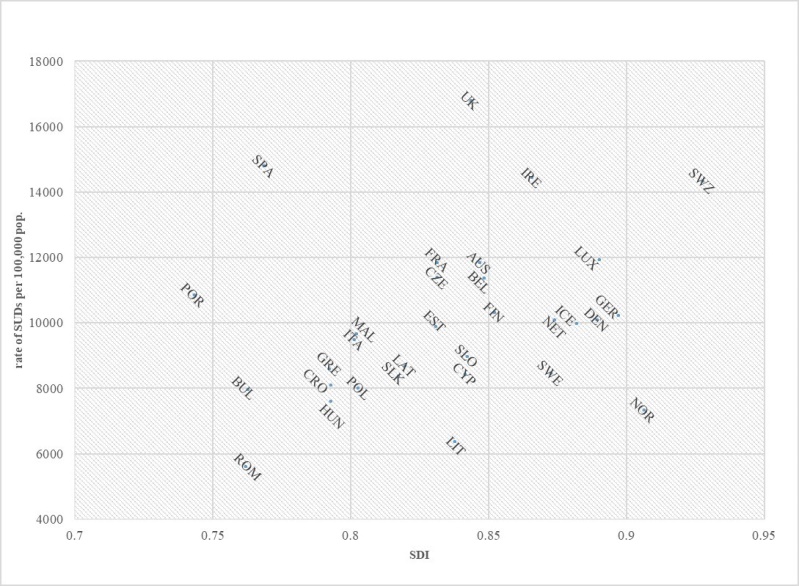


- Supplement Figure 2: Correlation between incidence rates of self-harm and Socio Demographic Index (SDI), in 31 European countries, year 2019, both sexes, age 10-24


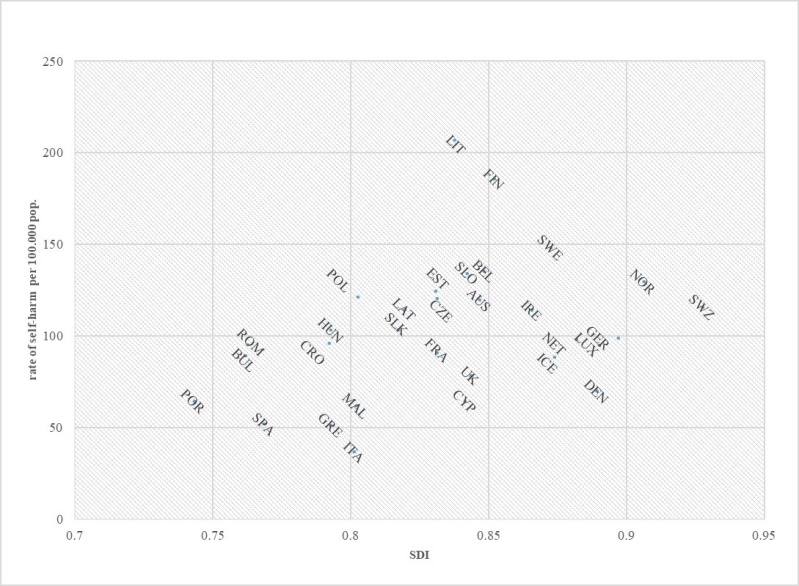


- Supplement Figure 3: Correlation between prevalence rates of substance use disorders (SUDs) and mental disorders (MDs), in 31 European countries, year 2019, both sexes, age 10-24


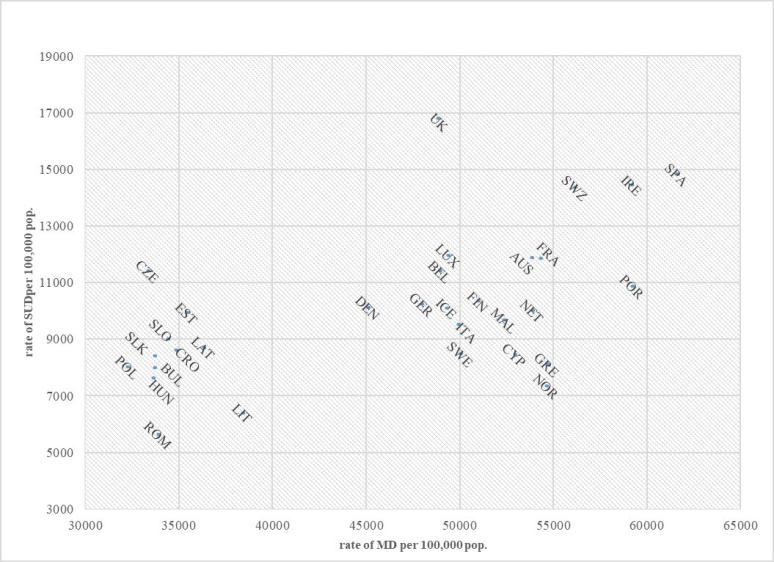

Supplement: Supplementary file 1 [file mmc1.docx]
