# Supplementary material for: The burden of mental disorders, substance use disorders and self-harm among young people in Europe, 1990–2019: Findings from the Global Burden of Disease Study 2019
Source: Lancet Reg Health Eur. 2022 Apr 1;16:100341. doi: 10.1016/j.lanepe.2022.100341 (PMC8980870; doi:10.1016/j.lanepe.2022.100341)
Supplement: Supplementary file 2 [file mmc2.docx]

**Supplement Table 1. Prevalence, Incidence, Years Lived with Disability (YLDs) and Years of Life Lost (YLLs), where available, for mental disorders, substance abuse and self-harm in European Union, Iceland, Norway and Switzerland, years 1990-2019, males and females, age 10-24, rates and percentage change over time**

|  |  | **Rates per 100,000 population**  **(95% Uncertainty Intervals)** | | |  | **Rates per 100,000 population**  **(95% Uncertainty Intervals)** | | | |
| --- | --- | --- | --- | --- | --- | --- | --- | --- | --- |
|  |  | **Males** | | |  | **Females** | | | |
|  |  | **1990** | **2019** | **Change %** |  | **1990** | **2019** | **Change %** | |
| **Mental disorders** | | | | | | | | |  |
| Anxiety disorders | Prevalence | 4,287∙0  (3,116∙0 ; 5,723∙9) | 4,625∙2  (3,466 ; 5,965∙2) | 5∙9  (1∙9 ; 10∙3) |  | 8,101∙2  (5,951ADHD ∙6 ;10,694∙6) | 8,627∙4  (6,500∙7 ; 11,030∙6) | 4∙5  (-0∙1 ; 9∙3) | |
|  | YLDs | 425∙5  (268∙7 ; 629∙3) | 459∙4  (300 ; 658) | 6  (1∙3 ; 11∙1) |  | 794∙5  (510∙6 ; 1,155∙6) | 846∙5  (562∙6 ; 1,195∙9) | 4∙5  (-0∙4 ; 9∙5) | |
| ADHD | Prevalence | 3,502∙9  (2,514 ; 4,723∙3) | 3,761∙8  (2,769∙7 ; 5,006∙1) | 5∙3  (0∙0 ; 11∙5) |  | 1,370∙6  (993∙3 ; 1,841∙5) | 1,480∙7  (1,089∙9 ; 1,952∙9) | 5∙9  (0∙5 - 11∙8) | |
|  | YLDs | 42∙8  (24 ; 71∙4) | 46∙0  (26∙4 ; 75∙4) | 5∙4  (-0∙8 ; 12∙2) |  | 16∙7  (9∙1 ; 28∙1) | 18∙0  29∙5 ; 10∙1 | 5∙9  (-1∙4 ; 12∙8) | |
| ASD | Prevalence | 909∙8  (801∙3 ; 1,025∙8) | 957∙0  (874∙2 ; 1,043∙3) | 3∙2  (1∙9 ; 4∙6) |  | 207∙2  (177∙6 ; 239∙8) | 212∙9  (190 ; 237∙3) | 0∙9  (-0∙8 ; 2∙6) | |
|  | YLDs | 141∙0  (96 ; 195∙5) | 148∙3  (104∙8 ; 199∙3) | 3∙2  (0∙3 ; 6∙3) |  | 31∙9  (20∙1 ; 46∙7) | 32∙8  (21∙5 ; 46∙4) | 0∙9  (-5 ; 6∙8) | |
| Bipolar disorder | Prevalence | 722∙0  (528∙4 ; 944∙6) | 749∙7  (570∙2 ; 948∙6) | 1∙9  (-1∙2 ; 5) |  | 914∙2  (669∙4 ; 1,193∙1) | 946∙5  (719 ; 1,192∙2) | 1∙6  (-2∙1 ; 5∙4) | |
|  | YLDs | 162∙2  (88 ; 256∙8) | 168∙6  (95∙6 ; 258∙3) | 2  (-3∙3 ; 7∙5) |  | 202  (111∙3 ; 317∙4) | 209∙0  (120 ; 320∙3) | 1∙6  (-3∙3 ; 7∙1) | |
| Conduct disorders | Prevalence | 2,312∙4  (1,750∙3 ; 2,937∙3) | 2,410∙1  (1,889∙8 ; 2,962∙8) | 2∙3  (1∙1 - 3∙7) |  | 1,345∙5  (919∙8 ; 1,861∙2) | 1,422∙8  (1,005∙2 ; 1,906∙2) | 3∙8  (2∙1 ; 5∙1) | |
|  | YLDs | 281∙4  (164∙4 ; 432∙8) | 293∙3  (177∙6 ; 436∙9) | 2∙3  (-0∙3 ; 5) |  | 162∙3  (87∙2 ; 262∙7) | 171∙8  (95∙1 ; 269∙2) | 3∙8  (0∙6 ; 7∙5) | |
| Depressive disorders | Prevalence | 2,309∙5  (1,805∙8 ; 2,866∙3) | 2,220∙1  (1,746∙8 ; 2,755∙6) | -5∙8  (-11 ; 0∙1) |  | 3,976∙5  (3,136∙7 ; 4,899∙7) | 3,994∙8  (3,108∙4 ; 4,943∙9) | -1∙6  (-7∙7 ; 5∙1) | |
|  | YLDs | 422∙3  (272∙2 ; 614) | 403∙8  (261∙9 ; 584∙2) | -6∙4  (-12∙3 ; 0∙8) |  | 741∙5  (482 ; 1,072∙3) | 745∙7  (484∙1 ; 1,077∙5) | -1∙5  (-8∙4 ; 5∙5) | |
| Eating disorders | Prevalence | 259  390∙8 ; 155∙4 | 310∙7  (194∙7 ; 452∙7) | 17∙8  (10∙7 ; 25∙8) |  | 951∙1  (648∙8 ; 1,340∙2) | 1,113∙8  (782∙2 ; 1,540∙9) | 14∙8  (8∙1 ; 21) | |
|  | YLDs | 55∙9  (29∙6 ; 93) | 67∙1  (36∙8 ; 109∙1) | 17∙9  (10∙7 ; 25∙9) |  | 203∙1  (118 ; 317∙7) | 237∙9  (142∙7 ; 364∙6) | 14∙9  (8∙2 ; 20∙8) | |
|  | YLLs | 0∙2  (0∙1 ; 0∙3) | 0∙1  (0∙0 ; 0∙2) | -66  (-81∙7 ; -36∙5) |  | 1∙8  (1∙2 ; 2∙7) | 2∙5  (1∙6 ; 4) | 42∙6  (9∙7 ; 80∙2) | |
| IDID | Prevalence | 699∙6  (288∙3 ; 1,070∙7) | 477∙9  (146∙1 ; 767∙7) | -34∙6  (-52∙2 ; -26∙5) |  | 760∙2  (427∙3 ; 1,057∙8) | 557∙2  (283∙6 ; 779∙6) | -28∙7  (-37∙4 ; -23∙6) | |
|  | YLDs | 31∙1  (11∙6 ; 53∙8) | 21∙9  (6∙6 ; 38∙7) | -32∙1  (-45∙4 ; -24∙5) |  | 34∙3  (16∙8 ; 55∙3) | 25∙8  (12∙1 ; 41∙4) | -26∙4  (-33∙3 ; -21∙2) | |
| Schizophrenia | Prevalence | 100  (69∙3 ; 138∙1) | 96∙8  (69 ; 131∙4) | -5∙1  (-8∙4 ; -1∙7) |  | 86∙3  (58∙6 ; 121∙6) | 83∙5  (58∙3 ; 115∙3) | -5∙1  (-8 ; -2) | |
|  | YLDs | 67  (38∙2 ; 103∙4) | 65∙1  (38∙2 ; 99∙3) | -4∙7  (-14∙2 ; 4∙8) |  | 56∙8  (31∙1 ; 89∙8) | 55∙1  (31∙2 ; 84∙7) | -4∙8  (-14∙3 ; 4∙2) | |
| Other mental disorders | Prevalence | 774∙6  (521∙4 ; 1,041) | 779∙1  (543∙2 ; 1,014∙1) | -1∙3  (-1∙8 ; -0∙8) |  | 465∙5  (295∙1 ; 648∙5) | 462∙2  (303∙9 ; 623∙9) | -2∙6  (-3∙1 ; -2) | |
|  | YLDs | 60  (31∙8 ; 95) | 60∙4  (33∙3 ; 92∙9) | -1∙2  (-7∙5 ; 5∙5) |  | 35∙6  (18 ; 57∙7) | 35∙3  (18∙5 ; 55∙6) | -2∙6  (-10∙2 ; 5∙7) | |
| **Substance use disorders** | | | | | | | | |  |
| Alcohol use disorders | Prevalence | 2,502∙5  (1,686∙7 ; 3,446∙3) | 2,295∙2  (1,561∙2 ; 3,122∙8) | -10∙2  (-17∙6 ; -4∙7) |  | 1,154∙9  (743∙5 ; 1,642∙4) | 1,153∙5  (757 ; 1,603∙3) | -2∙1  (-6∙4 ; 2∙7) | |
|  | YLDs | 257∙3  (151∙2 ; 401∙3) | 236∙1  (141∙0 ; 364∙6) | -10∙9  (-17∙7 ; -3∙9) |  | 117∙1  (66 ; 186∙9) | 116∙8  (66∙6 ; 183∙2) | -2∙3  (-8∙6 ; 3∙5) | |
|  | YLLs | 21∙1  (17∙7 ; 24∙9) | 15∙0  (11∙8 ; 18∙7) | -29∙8  (-40∙7 ; -20∙1) |  | 3∙9  (3∙1 ; 4∙8) | 3∙2  (2∙5 ; 3∙9) | -19∙7  (-8∙3 ; -30∙8) | |
| Drug use disorders | Prevalence | 2,686∙8  (2,110 ; 3,411∙6) | 2,700∙2  (2,279∙4 ; 3,226∙3) | -1∙1  (-7∙7 - 5∙2) |  | 1,559∙8  (1,211∙1 ; 2,013∙1) | 1,566∙3  (1,312∙8 ; 1,873∙3) | -1∙1  (-9∙3 ; 7∙3) | |
|  | YLDs | 268∙4  (182∙4 ; 367∙1) | 308∙6  (218∙2 ; 408∙8) | 12∙9  (5∙4 ; 21∙6) |  | 153∙0  (100∙1 ; 215∙5) | 192∙5  (131∙7 ; 261∙4) | 23∙6  (11∙8 ; 37∙2) | |
|  | YLLs | 120∙8  (102∙5 ; 141∙6) | 101∙2  (80∙9 ; 126∙8) | -17∙6  (-27∙7 ; -3∙3) |  | 32∙0  (27∙4 ; 37∙5) | 30∙7  (25∙6 ; 36∙9) | -5∙7  (-14∙7 ; 3∙9) | |
| **Self-harm** | | | | | | | | |  |
|  | Prevalence | 59∙8  (45∙9 ; 74∙1) | 43∙6  (34∙4 ; 52∙7) | -28∙6  (-30∙5 ; -26∙8) |  | 60∙9  (46∙3 ; 76∙7) | 48∙8  (38∙2 ; 59∙8) | -21∙4  (-24∙4 ; -18∙4) | |
|  | YLDs | 4∙1  (2∙8 ; 5∙5) | 2∙8  (2 ; 3∙7) | -32∙7  (-35∙7 ; -29∙8) |  | 4∙5  (3 ; 6∙2) | 3∙4  (2∙4 ; 4∙6) | -25∙6  (-29∙8 ; -21∙3) | |
|  | YLLs | 772∙9  (728∙7 ; 819∙2) | 494∙0  (447∙9 ; 545∙1) | -37∙3  (-41∙5 ; -31∙9) |  | 220∙4  (203∙0 ; 239∙5) | 134∙7  (120∙4 ; 150∙9) | -40  (-44∙0 ; -36∙0) | |
| YLDs, years lived with a disability; YLLs Years of Life Lost; ADHD, Attention deficit/hyperactivity disorder; ASD Autism spectrum disorders; IDID Idiopathic developmental intellectual disability | | | | | | | | |  |

**Supplement Table 2: Prevalence, Incidence, Years Lived with Disability (YLDs) and Years of Life Lost (YLLs), where available, for mental disorders, substance abuse and self-harm in European Union, Iceland, Norway and Switzerland, years 1990-2019, males and females, age 10-14, rates and percentage change over time**

|  |  | **Rates per 100,000 population**  **(95% Uncertainty Intervals)** | | |  | **Rates per 100,000 population**  **(95% Uncertainty Intervals)** | | | |
| --- | --- | --- | --- | --- | --- | --- | --- | --- | --- |
|  |  | **Males** | | |  | **Females** | | | |
|  |  | **1990** | **2019** | **Change %** |  | **1990** | **2019** | **Change %** |  |
| **Mental disorders** | | | | | | | | |  |
| Anxiety disorders | Prevalence | 3,793∙5  (2,592∙8 ; 5,187∙9) | 4,159∙5  (2,944∙8 ; 5,454∙7) | 7∙7  (2∙8 ; 12∙8) |  | 7,110∙1  (4,929; 9,638∙9) | 7,728∙3  (5,523∙6 ; 10,049∙1) | 6∙8  (1∙5 ; 12) |  |
|  | YLDs | 380∙0  (230∙6 ; 576∙3) | 416∙4  (262∙2 ; 610∙4) | 7∙7  (1 ; 14∙1) |  | 708∙0  (436∙4 ; 1,050∙7) | 770∙1  (490∙3 ; 1,108∙4) | 6∙8  (0∙3 ; 13) |  |
| ADHD | Prevalence | 4,948∙4  (3,503∙9 ; 6,631∙5) | 5,378∙0  (3,897∙2 ; 7,130∙3) | 6∙6  (0∙5 ; 14) |  | 1,851∙5  (1,328∙9 ; 2,480∙2) | 2,005∙7  (1,455∙9 ; 2,636∙8) | 6∙2  (0∙5 ; 12∙6) |  |
|  | YLDs | 60∙6  (34∙4 ; 100∙6) | 65∙9  (38 ; 108∙5) | 6∙7  (-1∙2 ; 15) |  | 22∙6  (12∙5 ; 37∙9) | 24∙5  (13∙8 ; 40∙1) | 6∙3  (-2∙4 ; 15∙6) |  |
| ASD | Prevalence | 920∙9  (812∙6 ; 1,036∙5) | 980∙8  (896∙8 ; 1,068∙6) | 4∙6  (3∙2 ; 6) |  | 211∙6  (181∙2 ; 244∙3) | 218∙3  (194∙6 ; 243) | 1∙3  (-0∙4 ; 3∙1) |  |
|  | YLDs | 143∙7  (98∙4 ; 199∙2) | 153∙1  (107∙7 ; 205∙7) | 4∙6  (0∙1 ; 9∙2) |  | 32∙9  (20∙7 ; 48∙1) | 34∙0  (22∙3 ; 48) | 1∙6  (-6∙3 ; 9∙3) |  |
| Bipolar disorder | Prevalence | 234∙3  (159∙9 ; 323∙7) | 254∙4  (179∙9 ; 338∙1) | 6∙7  (2∙7 ; 10∙9) |  | 276∙9  (186∙8 ; 383∙3) | 302∙5  (211∙9 ; 403∙6) | 7∙4  (2∙4 ; 12∙4) |  |
|  | YLDs | 53∙2  (25∙4 ; 90) | 57∙9  (29 ; 94∙9) | 7∙1  (-6∙2 ; 21∙9) |  | 62∙5  (29∙5 ; 104) | 68∙4  (34∙3 ; 110∙5) | 7∙9  (-5 ; 22∙5) |  |
| Conduct disorders | Prevalence | 4,246∙5  (3,244∙4 ; 5,335∙9) | 4,329∙2  (3,418 ; 5,273∙2) | 0∙1  (-1 ; 1∙3) |  | 2,632∙1  (1,816∙9 ; 3,548∙6) | 2,692∙3  (1,916∙7 ; 3,525∙9) | 0∙4  (-0∙9 ; 1∙7) |  |
|  | YLDs | 519∙3  (306∙9 ; 798∙1) | 529∙6  (322∙1 789∙1) | 0∙2  (-2∙9 ; 3∙4) |  | 319∙7  (174∙7 ; 519∙1) | 327∙2  (183∙9 ; 514∙1) | 0∙5  (-3∙3 ; 4∙9) |  |
| Depressive disorders | Prevalence | 1,014∙7  (714∙9 ; 1,366∙5) | 1,042∙6  (730∙8 ; 1,398∙5) | 0∙6  (-7∙7 ; 8∙6) |  | 1,854∙4  (1,328 ; 2,440∙1) | 1,942∙3  (1,356∙4 ; 2,598∙9) | 2∙4  (-7∙2 ; 12∙1) |  |
|  | YLDs | 193∙7  (113∙7 ; 305∙6) | 199∙5  (118∙1 ; 311∙2) | 0∙8  (-9∙4 ; 10∙6) |  | 360∙4  (216∙6 ; 560) | 378∙3  (223∙6 ; 589∙4) | 2∙5  (-8∙7 ; 13∙5) |  |
| Eating disorders | Prevalence | 105∙7  (56∙6 ; 168∙6) | 129∙6  (75∙2 ; 198∙1) | 20∙7  (4∙3 ; 39) |  | 331∙1  (202∙3 ; 503) | 401∙1  (251∙9 ; 600∙1) | 18∙7  (6∙1 ; 29∙9) |  |
|  | YLDs | 23∙0  (11 ; 39∙5) | 28∙2  (14∙1 ; 46∙9) | 20∙7  (4∙2 ; 38∙8) |  | 71∙7  (38∙2 ; 116∙4) | 86∙9  (47∙4 ; 139∙1) | 18∙7  (5∙9 ; 29∙9) |  |
|  | YLLs | - | - | - |  | 0∙2  (0∙1 ; 0∙4) | 0∙3  (0∙1 ; 0∙6) | 25∙3  (-26∙0 ; 102∙7) |  |
| IDID | Prevalence | 738∙1  (309∙4 ; 1,121∙9) | 495∙4  (152∙9 ; 794∙5) | -35∙7  (-53∙3 ; -27∙6) |  | 800∙2  (459∙2 ; 1,105∙1) | 580∙9  (300∙8 ; 808∙5) | -29∙3  (-38∙1 ; -24∙3) |  |
|  | YLDs | 32∙5  (12∙2 ; 55∙9) | 22∙7  (6∙8 ; 40∙2) | -32∙7  (-47∙3 ; -24) |  | 36∙2  (18 ; 58∙3) | 27∙2  (13 ; 43∙4) | -26∙7  (-35∙1 ; -19∙3) |  |
| Schizophrenia | Prevalence | 6∙8  (3∙3 ; 11∙7) | 6∙7  (3∙4 ; 11∙2) | -2∙3  (-6 ; 1∙6) |  | 6∙1  (3 ; 10∙6) | 6∙2  (3∙2 ; 10∙4) | -0∙3  (-3∙9 ; 3∙2) |  |
|  | YLDs | 4∙8  (2∙3 ; 8∙4) | 4∙8  (2∙3 ; 8∙1) | -2∙3  (-6 ; 1∙6) |  | 4∙3  (2∙1 ; 7∙6) | 4∙4  (2∙1 ; 7∙5) | -0∙3  (-3∙9 ; 3∙2) |  |
| Other mental disorders | Prevalence | 90∙4  (59∙5 ; 123) | 93∙8  (63∙9 ; 123∙6) | 1∙9  (1 ; 2∙8) |  | 53∙8  (33∙1 ; 75∙9) | 55∙5  (35∙4 ; 75∙8) | 1∙2  (0∙0 ; 2∙4) |  |
|  | YLDs | 7∙2  (3∙7 ; 1∙6) | 7∙5  (3∙9 ; 11∙8) | 2∙2  (-8∙5 ; 14∙2) |  | 4∙3  (2∙2 ; 7) | 4∙4  (2∙4 ; 7) | 1∙3  (-5∙2 ; 7∙7) |  |
| **Substance use disorders** | | | | | | | | |  |
| Alcohol use disorders | Prevalence | 115∙8  (67∙2 ; 183∙3) | 116∙1  (69∙6 ; 180) | -1∙6  (-5∙7 ; 2∙8) |  | 98∙9  (57∙4 ; 155∙5) | 101∙4  (60∙7 ; 155∙8) | 0∙5  (-7∙4 ; 6∙5) |  |
|  | YLDs | 11∙9  (5∙5 ; 21∙4) | 11∙9  (5∙7 ; 21∙2) | -1∙6  (-13 ; 10∙7) |  | 10∙2  (4∙6 ; 18∙4) | 10∙4  (4∙8 ; 18∙2) | 0∙5  (-13∙0 ; 14∙5) |  |
| Drug use disorders | Prevalence | 300∙5  (180∙5 ; 455∙6) | 270∙8  (174∙9 ; 390∙9) | -11∙0  (-21∙2 ; 0∙8) |  | 177∙9  (105 ; 270) | 162∙9  (104∙3 ; 231∙7) | -9∙4  (-21∙3 ; 5∙5) |  |
|  | YLDs | 10∙9  (5∙6 ; 18∙6) | 10∙9  (6 ; 17∙1) | -1∙3  (-17∙4 ; 18∙2) |  | 6∙5  (3∙2 ; 11∙1) | 6∙6  (3∙6 ; 10∙3) | 0∙4  (-19∙1 ; 22) |  |
| **Self-harm** | | | | | | | | |  |
|  | Incidence | 27∙3  (14∙6 ; 47∙1) | 20∙8  (10∙2 ; 38∙9) | -37∙2  (-44∙2 ; -30∙0) |  | 42∙7  (21∙0 ; 78∙3) | 39∙1  (17∙9 ; 75∙8) | -24∙2  (-31∙0 ; -18∙7) |  |
|  | YLDs | 0∙6  (0∙4 ; 0∙8) | 0∙4  (0∙3 ; 0∙6) | -30∙0  (-34∙6 ; -24∙9) |  | 0∙9  (0∙6 ; 1∙2) | 0∙7  (0∙5 ; 1) | -18∙6  (-24∙1 ; -12∙9) |  |
|  | YLLs | 111∙2  (93∙9 ; 130∙4) | 52∙6  (40∙5 ; 66∙9) | -53∙5  (-59∙8 ; -45∙5) |  | 38∙0  (31∙2 ; 46∙2) | 22∙1  (17∙5 ; 27∙4) | -42∙8  (-50∙9 ; -33∙9) |  |
| YLDs, years lived with a disability; YLLs Years of Life Lost; ADHD, Attention deficit/hyperactivity disorder; ASD Autism spectrum disorders; IDID Idiopathic developmental intellectual disability | | | | | | | | |  |

**Supplement Table 3∙ Prevalence, Incidence, Years Lived with Disability (YLDs) and Years of Life Lost (YLLs), where available, for mental disorders, substance abuse and self-harm in European Union, Iceland, Norway and Switzerland, years 1990-2019, males and females, age 15-19, rates and percentage change over time**

|  |  | **Rates per 100,000 population**  **(95% Uncertainty Intervals)** | | |  | **Rates per 100,000 population**  **(95% Uncertainty Intervals)** | | | |
| --- | --- | --- | --- | --- | --- | --- | --- | --- | --- |
|  |  | **Males** | | |  | **Females** | | | |
|  |  | **1990** | **2019** | **Change %** |  | **1990** | **2019** | **Change %** |  |
| **Mental disorders** | | | | | | | | |  |
| Anxiety disorders | Prevalence | 4,617∙5  (3,535∙2 ; 5,995∙5) | 5,009∙4  (3,928∙9 ; 6,269∙9) | 6∙5  (2∙4 ; 10∙9) |  | 8,688∙2  (6,649∙3 ; 11,105∙8) | 9,336∙3  (7,326∙6 ; 11,585∙9) | 5∙5  (0∙8 ; 10∙4) |  |
|  | YLDs | 457∙7  (300∙6 ; 658∙8) | 497∙0  (335∙3 ; 691∙7) | 6∙6  (0∙6 ; 12∙6) |  | 851∙4  (566∙1 ; 1,208∙8) | 914∙5  (630 ; 1,256∙4) | 5∙4  (-0∙2 ; 10∙9) |  |
| ADHD | Prevalence | 3,515∙1  (2,552∙8 ; 4,716∙1) | 3,677∙4  (2,740∙5 ; 4,862∙2) | 2∙6  (-2∙4 ; 8∙4) |  | 1,390∙2  (1,014∙7 ; 1,846∙5) | 1,467∙3  (1,088∙1 ; 1,926∙4) | 3∙5  (-2∙0 ; 9) |  |
|  | YLDs | 42∙9  (23∙9 ; 71∙8) | 44∙9  (25∙8 ; 73∙5) | 2∙6  (-4∙3 ; 10∙3) |  | 16∙9  (9∙2 ; 28∙5) | 17∙8  (10 ; 29∙1) | 3∙4  (-6∙6 ; 13) |  |
| ASD | Prevalence | 909∙9  (803∙1 ; 1,024∙5) | 956∙6  (874∙6 ; 1,042) | 3∙2  (1∙8 ; 4∙6) |  | 207∙1  (177∙7 ; 239∙3) | 212∙7  (189∙9 ; 236∙9) | 0∙8  (-0∙9 ; 2∙6) |  |
|  | YLDs | 140∙8  (95∙6 ; 194∙7) | 148∙0  (105∙1 ; 198∙6) | 3∙2  (-1∙8 ; 8∙1) |  | 31∙8  (20∙1 ; 46∙5) | 32∙7  (21∙5 ; 46) | 0∙9  (-8∙6 ; 11∙2) |  |
| Bipolar disorder | Prevalence | 864∙1  (617∙8 ; 1,157∙2) | 919∙3  (682∙5 ; 1,188∙6) | 4∙5  (0∙9 ; 8∙1) |  | 1,051∙6  (750∙8 ; 1,409∙2) | 1,126∙6  (831∙8 ; 1,452∙7) | 5∙2  (0∙9 ; 9∙6) |  |
|  | YLDs | 194∙4  (103∙3 ; 308∙2) | 206∙8  (115∙8 ; 318∙1) | 4∙6  (-3∙2 ; 12∙5) |  | 233∙2  (126∙9 ; 369∙9) | 249∙7  (141∙3 ; 387∙3) | 5∙2  (-2∙4 ; 13∙2) |  |
| Conduct disorders | Prevalence | 2,931∙8  (2,197∙6 ; 3,769∙1) | 3,003∙8  (2,332∙5 ; 3,741∙3) | 0∙6  (-1 ; 2∙1) |  | 1,581∙5  (1,068∙2 ; 2,268∙6) | 1,622∙2  (1,131∙4 ; 2,256∙0) | 0∙6  (-1∙2 ; 2∙6) |  |
|  | YLDs | 354∙5  (204∙3 ; 544) | 362∙9  (218∙4 ; 540) | 0∙5  (-3∙4 ; 4∙2) |  | 188∙9  (99∙1 ; 303) | 193∙5  (104∙5 ; 302∙1) | 0∙6  (-4∙4 ; 6∙3) |  |
| Depressive disorders | Prevalence | 2,418∙5  (1,913∙8 ; 2,966∙1) | 2,368∙5  2,920∙6 1,873∙4 | -4∙1  (-10∙8 ; 2∙9) |  | 4,292∙6  (3,430∙1 ; 5,233∙1) | 4,413∙8  (3,498∙6 ; 5,414) | 0∙7  (-6∙7 ; 8∙4) |  |
|  | YLDs | 447∙9  (290∙7 ; 654) | 436∙7  (284∙5 ; 635∙9) | -4∙6  (-12∙8 ; 3∙6) |  | 809∙2  (528∙3 ; 1,169∙7) | 832∙8  (548∙4 ; 1,199∙2) | 0∙7  (-7∙6 ; 9∙1) |  |
| Eating disorders | Prevalence | 290∙1  (182∙7 ; 440∙9) | 356∙3  (233∙6 ; 522∙3) | 20∙6  (9 ; 32∙3) |  | 1,070∙7  1,574∙8 741∙2 | 1,269∙2  (897∙6 ; 1,851∙5) | 16∙1  (5∙7 , 24∙6) |  |
|  | YLDs | 62∙7  (33∙9 ; 104) | 77∙0  (42∙7 ; 126∙4) | 20∙6  (9∙1 ; 32∙5) |  | 229∙3  (131∙3 ; 370∙9) | 271∙8  (159∙7 , 431∙9) | 16∙1  (5∙7 ; 24∙8) |  |
|  | YLLs | - | - | - |  | 2∙0  (1∙3 ; 3∙1) | 2∙8  (1∙6 ; 4∙9) | 26∙8  (35∙8 ; 19∙5) |  |
| IDID | Prevalence | 703∙7  (289∙7 ; 1,076∙7) | 479∙2  (145∙6 ; 770∙7) | -34∙8  (-52∙5 ; -26∙8) |  | 766∙7  (431∙7 ; 1,066) | 558∙3  (283∙3 ; 781∙5) | -29∙2  (-38∙1 ; -24) |  |
|  | YLDs | 31∙1  (11∙5 ; 54) | 21∙9  (6∙5 ; 38∙6) | -32∙2  (-46∙0 ; -23∙3) |  | 34∙5  (16∙9 ; 55∙5) | 25∙8  (12 ; 41∙3) | - 43∙6  (-1∙8 ; - 10∙2) |  |
| Schizophrenia | Prevalence | 59∙2  (39∙8 ; 85∙9) | 58∙6  (40∙4 ; 82∙9) | -2∙9  (-5∙7 ; 0∙4) |  | 52  (34∙4 ; 76∙2) | 52∙3  (35∙4 ; 74∙5) | -1∙2  (-4∙2 ; 1∙9) |  |
|  | YLDs | 39∙8  (18∙6 ; 67∙5) | 39∙6  (18∙7 ; 65∙6) | -2∙0  (-20∙7 ; 18) |  | 34∙6  (15∙2 ;59∙3) | 34∙8  (16 ; 58∙3) | -0∙8  (-19 ; 20∙4) |  |
| Other mental disorders | Prevalence | 618∙0  (407∙4 ; 839∙8) | 641∙2  (438∙0 ; 843∙2) | 1∙9  (1 ; 2∙8) |  | 367∙5  (225∙6 ; 517∙9) | 379∙1  (241∙7 ; 517∙4) | 1∙3  (0∙3 ; 2∙3) |  |
|  | YLDs | 48∙0  (24∙1 ; 77∙5) | 49∙7  (25∙6 ; 78∙2) | 1∙9  (-9∙8 ; 14∙7) |  | 28∙1  (13 ; 47∙9) | 29∙1  (13∙8 ; 47∙7) | 1∙8  (-13∙1 ; 18) |  |
| **Substance use disorders** | | | | | | | | |  |
| Alcohol use disorders | Prevalence | 1,776∙2  (1,192∙5 ; 2,441∙4) | 1,710∙1  (1,156∙5 ; 2,313∙9) | -5∙8  (-12∙7 ; -0∙2) |  | 945∙8  (635∙9 ; 1,290∙8) | 970∙6  (663∙1 ; 1,297∙9) | 0∙6  (-4∙1 ; 5∙4) |  |
|  | YLDs | 182∙9  (104∙4 ; 283∙9) | 176∙2  (101∙6 ; 270∙2) | -5∙7  (-14∙3 ; 2∙1) |  | 96∙4  (54∙5 ; 149∙7) | 98∙7  (56∙2 ; 150∙3) | 0∙4  (-9∙2 ; 9∙8) |  |
|  | YLLs | 16∙2  (13 ; 19∙8) | 11∙3  (8∙6 ; 14∙4) | -31∙2  (-44∙7 ; -19∙3) |  | 4∙2  (3∙4 ; 5∙3) | 3∙1  (2∙5 ; 3∙8) | -28∙5  (-41∙4 ; -15∙2) |  |
| Drug use disorders | Prevalence | 3,589∙0  (2,716 ; 4,729∙2) | 3,472∙4  (2,881∙9 ; 4,271∙5) | -4∙6  (-12∙3 ; 3∙1) |  | 2,125∙6  (1,597∙1 ; 2,864∙3) | 2,063∙7  (1,704∙1 ; 2,532) | -4∙1  (-13∙1 ; 5∙5) |  |
|  | YLDs | 243∙2  (161∙8 ; 342∙5) | 278∙7  (194∙3 ; 379∙6) | 12∙6  (1∙8 ; 23∙9) |  | 140∙0  (89∙9 ; 202∙5) | 169∙7  (114∙1 ; 234∙5) | 19∙3  (4∙9 ; 35∙4) |  |
|  | YLLs | 77∙8  (64∙5 ; 93∙4) | 70∙0  (53∙8 ; 92∙6) | -11∙3  (-25 ; 10∙1) |  | 29∙0  (24∙5 ; 34∙5) | 31∙5  (26∙2 ; 38∙1) | 6∙9  (-4∙6 ; 19∙6) |  |
| **Self-harm** | | | | | | | | |  |
|  | Incidence | 115∙8  (82∙0 ; 158∙4) | 85∙7  (56∙8 ; 124∙9) | -42∙9  (-47∙3 ; -38∙6) |  | 131∙3  (87∙1 ; 186∙9) | 111∙2  (67∙6 ; 168∙7) | -35∙8  (-42∙0 ; -30∙4) |  |
|  | YLDs | 3∙1  (2∙2 ; 4∙2) | 2∙2  (1∙5 ; 2∙9) | -32∙0  (-35∙6 ; -28∙3) |  | 3∙8  (2∙6 ; 5∙3 ) | 3∙0  (2∙1 ; 4∙1) | -21∙2  (-26∙2 ; -15∙6) |  |
|  | YLLs | 728  (679∙5 ; 778∙3) | 473∙8  (422∙9 ; 530∙6) | -36∙1  (-42∙1 ; -29∙4) |  | 242∙9  (221∙3 ; 266∙3) | 162∙3  (143∙3 ; 183∙6) | -34∙4  (-40∙5 ; -27∙9) |  |
| YLDs, years lived with a disability; YLLs Years of Life Lost; ADHD, Attention deficit/hyperactivity disorder; ASD Autism spectrum disorders; IDID Idiopathic developmental intellectual disability | | | | | | | | |  |

**Supplement Table 4∙ Prevalence, Incidence, Years Lived with Disability (YLDs) and Years of Life Lost (YLLs), where available, for mental disorders, substance abuse and self-harm in European Union, Iceland, Norway and Switzerland, years 1990-2019, males and females, age 20-24, rates and percentage change over time**

|  |  | **Rates per 100,000 population**  **(95% Uncertainty Intervals)** | | |  | **Rates per 100,000 population**  **(95% Uncertainty Intervals)** | | | |
| --- | --- | --- | --- | --- | --- | --- | --- | --- | --- |
|  |  | **Males** | | |  | **Females** | | | |
|  |  | **1990** | **2019** | **Change %** |  | **1990** | **2019** | **Change %** |  |
| **Mental disorders** | | | | | | | | |  |
| Anxiety disorders | Prevalence | 4,414∙1  (3,185 ; 5,945∙6) | 4,709∙2  (3,528∙7 ; 6,167∙7) | 4∙6  (0∙3 ; 9∙5) |  | 8,419∙4  (6,193∙0 ; 11,235∙3) | 8,828∙3  (6,666∙5 ; 11,457∙5) | 2∙9  (-1∙8 ; 7∙7) |  |
|  | YLDs | 435∙7  (272∙5 ; 648∙6) | 465∙2  (302∙9 ; 671∙8) | 4∙7  (-1∙4 ; 11) |  | 816∙9  (523∙7 ; 1,197∙8) | 856∙3  (568∙9 ; 1,223∙6) | 2∙8  (-2∙7 ; 8∙8) |  |
| ADHD | Prevalence | 2,201∙9  (1,597∙6 ; 3,022∙4) | 2,286∙4  (1,712∙6 ; 3,097∙4) | 1∙7  (-2∙9 ; 7∙1) |  | 931∙7  (680∙9 ; 1,276∙7) | 983∙4  (736∙2 ; 1,313∙3) | 3∙5  (-1∙8 ; 9) |  |
|  | YLDs | 26∙8  (14∙8 ; 44∙9) | 27∙8  (16 ; 45∙3) | 1∙9  (-5∙4 ; 9∙9) |  | 11∙3  (6 ; 19∙1) | 11∙9  (6∙5 ; 19∙5) | 3∙4  (-6∙9 ; 13∙6) |  |
| ASD | Prevalence | 899∙8  (789∙5 ; 1,017∙3) | 934∙5  (852∙1 ; 1,020∙1) | 1∙9  (0∙7 ; 3∙1) |  | 203∙5  (174∙3 ; 236∙2) | 207∙8  (185∙6 , 231∙9) | 0∙2  (-1∙4 ; 1∙8) |  |
|  | YLDs | 138∙7  (94∙1 ; 192∙9) | 144  (101∙7 ; 193∙9) | 1∙8  (-3∙2 ; 7) |  | 31∙1  (19∙6 ; 45∙6) | 31∙6  (20∙8 ; 45∙1) | 0∙1  (-10∙7 ; 12∙4) |  |
| Bipolar disorder | Prevalence | 1,022∙4  (771∙6 ; 1,298∙2) | 1,065∙7  (839∙2 ; 1,308∙7) | 2∙2  (-0∙5 ; 5∙3) |  | 1,343∙1  (1,014∙1 ; 1,700∙8) | 1,401∙4  (1,104∙5 ; 1,711∙5) | 2∙4  (-0∙9 ; 5∙6) |  |
|  | YLDs | 228∙9  (129∙1 ; 357∙1) | 238∙9  (140∙5 ; 359) | 2∙4  (-5 ; 9∙7) |  | 294∙7  (167∙9 ; 455∙4) | 307∙0  (183∙1 ; 460∙8) | 2∙2  (-3∙7 ; 8∙7) |  |
| Conduct disorders | Prevalence | - | - |  |  | - | - |  |  |
|  | YLDs | - | - |  |  | - | - |  |  |
| Depressive disorders | Prevalence | 3,361∙1  (2,673∙1 ; 4,113∙6) | 3,212∙7  (2,604∙1 ; 3,905∙8) | -6∙4  (-11∙9 ; -0∙2) |  | 5,536∙7  (4,439∙6 ; 6,744∙3) | 5,591∙6  (4,440∙1 ; 6,777) | -1∙2  (-8∙2 ; 6∙1) |  |
|  | YLDs | 601∙9  (395∙6 ; 852) | 569∙2  (378∙7 ; 798) | -7∙5  (-14∙1 ; 0∙1) |  | 1,011∙3  (670∙3 ; 1,430∙3) | 1,020∙1  (676∙2 ; 1,436∙4) | -1∙4  (-9∙7 ; 6∙8) |  |
| Eating disorders | Prevalence | 366∙2  (217∙5 ; 542∙2) | 441∙6  (272∙7 ; 631∙8) | 18∙3  (8∙2 ; 29∙3) |  | 1,381∙6  (951∙7 ; 1,854∙7) | 1,659  (1,187∙9 ; 2,160∙4) | 17∙8  (10∙6 ; 25∙9) |  |
|  | YLDs | 78∙8  (42 ; 130∙4) | 95∙2  (53∙1 ; 152∙6) | 18∙4  (8∙4 ; 29∙6) |  | 293∙5  (175∙1 ; 444∙4) | 352∙4  (219∙1 ; 520) | 17∙8  (10∙8 ; 25∙8) |  |
|  | YLLs | 0∙3  (0∙2 ; 0∙6) | 0∙1  (0∙0 ; 0∙3) | -63∙8  (-84∙4 ; -19∙7) |  | 2∙9  (2 ; 4∙3) | 4∙5  (3 ; 6∙6) | 52  (8∙5 ; 103) |  |
| IDID | Prevalence | 661∙3  (268∙2 ; 1,019∙3) | 459∙7  (139∙9 ; 738∙9) | -33∙4  (-50∙8 ; -25∙4) |  | 719∙0  (395∙3 ; 1,008∙5) | 533∙1  (267∙2 ; 749∙8) | -27∙9  (-37 ; -22∙8) |  |
|  | YLDs | 29∙9  (11∙1 ; 51∙7) | 21∙3  (6∙4 ; 37∙5) | -31∙4  (-46∙2 ;-23) |  | 32∙4  (15∙6 ; 52∙5) | 24∙6  (11∙2 ; 39∙4) | -26∙1  (-35∙4 ; -19) |  |
| Schizophrenia | Prevalence | 221∙8  (155∙7 ; 301) | 219∙8  (159 ; 293∙2) | -3∙0  (-6∙8 ; 0∙7) |  | 188∙4  (129∙6 ; 261∙5) | 188∙1  (133∙5 ; 255∙8) | -2∙1  (-5∙3 ; 1∙4) |  |
|  | YLDs | 148∙3  (88∙8 ; 222∙6) | 147∙4  (91∙1 ; 219∙1) | -2∙6  (-13∙8 ; 8∙3) |  | 123∙5  (71∙3 ; 190∙4) | 123∙6  (73∙8 ; 184∙8) | -1∙7  (-13 ; 9∙5) |  |
| Other mental disorders | Prevalence | 1,533∙3  (1,039∙5 ; 2,053∙9) | 1,569∙3  (1,103∙9 ; 2,033∙6) | 0∙4  (0∙0 ; 0∙7) |  | 917∙1  (588∙2 ; 1,272∙9) | 936∙3  (623∙7 ; 1,257∙8) | 0∙1  (-0∙3 ; 0∙6) |  |
|  | YLDs | 118∙6  (64 ; 186∙4) | 121∙4  (69 ; 184∙8) | 0∙5  (-7∙2 ; 8∙7) |  | 69∙8  (36∙2 ; 111∙4) | 71∙1  (38∙7 ; 110∙4) | 0∙0  (-9∙7 ; 10∙9) |  |
| **Substance use disorders** | | | | | | | | |  |
| Alcohol use disorders | Prevalence | 5,319∙8  (3,592∙7 ; 7,321∙9) | 4,947∙3  (3,379∙7 ; 6,723∙3) | -9∙1  (-16∙9 ; -2∙9) |  | 2,273∙7  (1,441∙5 ; 3,275∙6) | 2,349∙4  (1,522∙4 ; 3,300∙1) | 1∙2  (-3∙7 ; 6∙6) |  |
|  | YLDs | 546∙6  (324∙9 ; 853) | 508∙7  (308∙5 ; 784∙7) | -9∙0  (-17∙1 ; -1∙8) |  | 230∙0  (30 ; 369∙5) | 237∙2  (136∙4 ; 375) | 0∙9  (-6∙2 ; 8∙3) |  |
|  | YLLs | 44∙5  (37∙8 ; 52) | 33∙1  (26∙3 ; 40∙8) | -27∙0  (-38 ; -16∙1) |  | 7∙0  (5∙5 ; 8∙7) | 6∙3  (5∙1 ; 7∙9) | -10∙3  (-23∙5 ; 3∙9) |  |
| Drug use disorders | Prevalence | 3,960∙4  (3,252∙3 ; 4,804∙8) | 4,306∙3  (3,732∙9 ; 4,965) | 6∙8  (0∙2 ; 13∙2) |  | 2,239∙5  2,744∙6 1,815∙4 | 2,458∙1  2,843∙5 2,115∙5 | 7∙9  (-0∙4 ; 16∙9) |  |
|  | YLDs | 521∙9  (359∙2 ; 702∙4) | 623∙4  (445∙1 ; 813∙6) | 17∙2  (8∙7 ; 26∙8) |  | 293∙2  (194∙1 ; 406∙9) | 394∙8  (272∙7 ; 531) | 32∙2  (18∙5 ; 49∙1) |  |
|  | YLLs | 269∙2  (229∙4 ; 314∙3) | 228∙2  (184∙3 ; 281∙2 | -16∙7  (-28∙2 ; -2∙3) |  | 62∙8  (54 ; 73∙3) | 59∙8  (49∙8 ; 71∙7) | -6∙5  (-17∙6 ; 5∙7) |  |
| **Self-harm** | | | | | | | | |  |
|  | Incidence | 183∙0  (138∙4 ; 245∙4) | 140∙2  (100∙0 ; 199∙1) | -40∙8  (-44∙4 ; -36∙9) |  | 174∙2  (125∙2 ; 241∙6) | 136∙1  (89∙8 ; 204∙8) | -41∙7  (-46∙6 ; -37∙2) |  |
|  | YLDs | 8∙1  (5∙6 ; 10∙8) | 5∙7  (4 ; 7∙4) | -31∙1  (-34∙7 ; -27∙8) |  | 8∙3  (5∙7 ; 11∙3) | 6∙3  (4∙5 ; 8∙4) | -25∙0  (-30∙3 ; -19∙5) |  |
|  | YLLs | 1,405∙8  (1,339∙4 ; 1,474∙4) | 937∙9  (863∙5 ; 1,019∙3) | -34∙6  (-39∙5 ; -28∙7) |  | 358∙8  (335∙8 ; 383∙9) | 217∙8  (198∙6 ; 239∙8) | -40∙4  (-45∙2 ; -35∙7) |  |
| YLDs, years lived with a disability; YLLs Years of Life Lost; ADHD, Attention deficit/hyperactivity disorder; ASD Autism spectrum disorders; IDID Idiopathic developmental intellectual disability | | | | | | | | |  |

**Supplement Table 5: Prevalence per 100,000 population aged 10-24 years of mental disorders in 31 European countries from 1990 to 2019**

|  | **1990** | **1991** | **1992** | **1993** | **1994** | **1995** | **1996** | **1997** | **1998** | **1999** | **2000** | **2001** | **2002** | **2003** | **2004** | **2005** | **2006** | **2007** | **2008** | **2009** | **2010** | **2011** | **2012** | **2013** | **2014** | **2015** | **2016** | **2017** | **2018** | **2019** |
| --- | --- | --- | --- | --- | --- | --- | --- | --- | --- | --- | --- | --- | --- | --- | --- | --- | --- | --- | --- | --- | --- | --- | --- | --- | --- | --- | --- | --- | --- | --- |
| **Austria** | 17,813 | 17,821 | 17,827 | 17,822 | 17,810 | 17,798 | 17,789 | 17,772 | 17,735 | 17,695 | 17,667 | 17,654 | 17,650 | 17,648 | 17,657 | 17,665 | 17,683 | 17,728 | 17,776 | 17,824 | 17,846 | 17,862 | 17,869 | 17,884 | 17,885 | 17,892 | 17,905 | 17,923 | 17,926 | 17,948 |
| **Belgium** | 16,149 | 16,136 | 16,122 | 16,114 | 16,112 | 16,122 | 16,142 | 16,175 | 16,206 | 16,243 | 16,264 | 16,279 | 16,309 | 16,334 | 16,371 | 16,404 | 16,423 | 16,426 | 16,420 | 16,410 | 16,408 | 16,405 | 16,403 | 16,411 | 16,416 | 16,412 | 16,390 | 16,365 | 16,328 | 16,295 |
| **Bulgaria** | 11,719 | 11,737 | 11,747 | 11,756 | 11,762 | 11,761 | 11,750 | 11,722 | 11,682 | 11,646 | 11,623 | 11,605 | 11,585 | 11,549 | 11,518 | 11,493 | 11,468 | 11,471 | 11,460 | 11,456 | 11,430 | 11,387 | 11,349 | 11,316 | 11,288 | 11,271 | 11,254 | 11,245 | 11,233 | 11,222 |
| **Croatia** | 11,963 | 11,967 | 11,971 | 11,970 | 11,970 | 11,962 | 11,963 | 11,950 | 11,945 | 11,932 | 11,918 | 11,889 | 11,854 | 11,803 | 11,760 | 11,723 | 11,693 | 11,658 | 11,621 | 11,596 | 11,588 | 11,595 | 11,609 | 11,624 | 11,639 | 11,635 | 11,636 | 11,629 | 11,627 | 11,624 |
| **Cyprus** | 17,546 | 17,514 | 17,480 | 17,470 | 17,451 | 17,451 | 17,461 | 17,477 | 17,497 | 17,507 | 17,511 | 17,512 | 17,518 | 17,520 | 17,526 | 17,536 | 17,531 | 17,516 | 17,506 | 17,496 | 17,485 | 17,493 | 17,514 | 17,543 | 17,567 | 17,581 | 17,559 | 17,538 | 17,555 | 17,607 |
| **Czech Rep** | 11,440 | 11,482 | 11,523 | 11,543 | 11,570 | 11,572 | 11,566 | 11,540 | 11,507 | 11,477 | 11,451 | 11,425 | 11,401 | 11,384 | 11,363 | 11,362 | 11,357 | 11,350 | 11,333 | 11,315 | 11,295 | 11,268 | 11,227 | 11,192 | 11,155 | 11,130 | 11,119 | 11,095 | 11,076 | 11,065 |
| **Denmark** | 15,553 | 15,543 | 15,517 | 15,493 | 15,469 | 15,452 | 15,410 | 15,355 | 15,293 | 15,215 | 15,160 | 15,112 | 15,084 | 15,057 | 15,040 | 15,022 | 15,005 | 14,978 | 14,957 | 14,948 | 14,947 | 14,957 | 14,965 | 14,972 | 14,983 | 14,983 | 14,983 | 14,970 | 14,989 | 15,041 |
| **Estonia** | 12,680 | 12,686 | 12,691 | 12,705 | 12,694 | 12,686 | 12,671 | 12,644 | 12,623 | 12,591 | 12,575 | 12,537 | 12,480 | 12,425 | 12,377 | 12,330 | 12,295 | 12,250 | 12,194 | 12,139 | 12,087 | 12,026 | 11,969 | 11,910 | 11,856 | 11,813 | 11,799 | 11,775 | 11,764 | 11,762 |
| **Finland** | 18,815 | 18,595 | 18,379 | 18,172 | 17,988 | 17,843 | 17,699 | 17,520 | 17,344 | 17,187 | 17,101 | 17,043 | 16,990 | 16,952 | 16,925 | 16,919 | 16,929 | 16,947 | 16,965 | 16,981 | 16,994 | 17,017 | 17,063 | 17,096 | 17,122 | 17,123 | 17,102 | 17,076 | 17,037 | 16,975 |
| **France** | 18,722 | 18,551 | 18,407 | 18,287 | 18,206 | 18,170 | 18,172 | 18,189 | 18,219 | 18,249 | 18,281 | 18,319 | 18,362 | 18,411 | 18,440 | 18,442 | 18,396 | 18,317 | 18,227 | 18,143 | 18,093 | 18,088 | 18,085 | 18,089 | 18,092 | 18,095 | 18,113 | 18,123 | 18,114 | 18,105 |
| **Germany** | 15,427 | 15,530 | 15,617 | 15,709 | 15,771 | 15,828 | 15,869 | 15,933 | 15,982 | 16,029 | 16,058 | 16,145 | 16,321 | 16,525 | 16,700 | 16,782 | 16,755 | 16,674 | 16,577 | 16,489 | 16,441 | 16,416 | 16,407 | 16,386 | 16,364 | 16,344 | 16,285 | 16,195 | 16,120 | 16,031 |
| **Greece** | 18,098 | 18,091 | 18,076 | 18,074 | 18,083 | 18,084 | 18,158 | 18,315 | 18,511 | 18,670 | 18,723 | 18,697 | 18,632 | 18,562 | 18,505 | 18,476 | 18,466 | 18,455 | 18,439 | 18,427 | 18,410 | 18,360 | 18,269 | 18,161 | 18,075 | 18,051 | 18,106 | 18,159 | 18,174 | 18,195 |
| **Hungary** | 11,697 | 11,726 | 11,746 | 11,763 | 11,779 | 11,779 | 11,766 | 11,741 | 11,702 | 11,670 | 11,643 | 11,614 | 11,579 | 11,548 | 11,525 | 11,500 | 11,480 | 11,466 | 11,445 | 11,426 | 11,407 | 11,380 | 11,341 | 11,300 | 11,261 | 11,232 | 11,225 | 11,223 | 11,215 | 11,203 |
| **Iceland** | 16,573 | 16,580 | 16,575 | 16,560 | 16,542 | 16,544 | 16,553 | 16,550 | 16,550 | 16,543 | 16,522 | 16,498 | 16,463 | 16,435 | 16,429 | 16,432 | 16,430 | 16,406 | 16,390 | 16,373 | 16,361 | 16,355 | 16,363 | 16,368 | 16,368 | 16,377 | 16,377 | 16,362 | 16,375 | 16,405 |
| **Ireland** | 18,916 | 18,953 | 18,983 | 19,008 | 19,020 | 19,029 | 18,943 | 18,720 | 18,445 | 18,202 | 18,099 | 18,093 | 18,105 | 18,110 | 18,128 | 18,161 | 18,382 | 18,878 | 19,457 | 19,946 | 20,162 | 20,172 | 20,175 | 20,171 | 20,185 | 20,208 | 20,384 | 20,530 | 20,258 | 19,664 |
| **Italy** | 17,021 | 16,915 | 16,807 | 16,697 | 16,595 | 16,496 | 16,396 | 16,290 | 16,187 | 16,104 | 16,059 | 16,050 | 16,057 | 16,068 | 16,081 | 16,082 | 16,087 | 16,114 | 16,149 | 16,186 | 16,218 | 16,269 | 16,361 | 16,459 | 16,541 | 16,581 | 16,573 | 16,561 | 16,583 | 16,637 |
| **Latvia** | 12,627 | 12,655 | 12,677 | 12,685 | 12,681 | 12,674 | 12,653 | 12,631 | 12,614 | 12,609 | 12,601 | 12,593 | 12,571 | 12,544 | 12,518 | 12,497 | 12,487 | 12,449 | 12,411 | 12,366 | 12,331 | 12,297 | 12,268 | 12,226 | 12,186 | 12,148 | 12,127 | 12,097 | 12,074 | 12,061 |
| **Lithuania** | 13,229 | 13,266 | 13,303 | 13,328 | 13,346 | 13,352 | 13,391 | 13,492 | 13,619 | 13,728 | 13,775 | 13,764 | 13,732 | 13,696 | 13,655 | 13,623 | 13,600 | 13,571 | 13,538 | 13,492 | 13,457 | 13,388 | 13,280 | 13,161 | 13,063 | 13,000 | 12,966 | 12,933 | 12,900 | 12,867 |
| **Luxembourg** | 16,937 | 16,910 | 16,880 | 16,862 | 16,846 | 16,832 | 16,818 | 16,800 | 16,793 | 16,783 | 16,750 | 16,713 | 16,674 | 16,631 | 16,589 | 16,562 | 16,550 | 16,526 | 16,500 | 16,473 | 16,469 | 16,467 | 16,475 | 16,477 | 16,471 | 16,472 | 16,468 | 16,438 | 16,435 | 16,463 |
| **Malta** | 17,506 | 17,491 | 17,478 | 17,462 | 17,451 | 17,428 | 17,417 | 17,408 | 17,390 | 17,379 | 17,372 | 17,367 | 17,373 | 17,392 | 17,409 | 17,422 | 17,420 | 17,399 | 17,376 | 17,349 | 17,337 | 17,351 | 17,378 | 17,407 | 17,422 | 17,437 | 17,387 | 17,338 | 17,362 | 17,440 |
| **Netherlands** | 17,119 | 17,208 | 17,300 | 17,373 | 17,438 | 17,480 | 17,527 | 17,591 | 17,667 | 17,730 | 17,783 | 17,859 | 17,967 | 18,082 | 18,177 | 18,199 | 18,194 | 18,180 | 18,164 | 18,154 | 18,153 | 18,170 | 18,227 | 18,298 | 18,364 | 18,383 | 18,159 | 17,934 | 17,947 | 17,991 |
| **Norway** | 18,534 | 18,520 | 18,495 | 18,463 | 18,431 | 18,405 | 18,332 | 18,197 | 18,047 | 17,919 | 17,859 | 17,905 | 18,044 | 18,216 | 18,372 | 18,450 | 18,463 | 18,454 | 18,437 | 18,417 | 18,409 | 18,398 | 18,376 | 18,350 | 18,331 | 18,335 | 18,421 | 18,488 | 18,408 | 18,212 |
| **Poland** | 11,042 | 11,067 | 11,089 | 11,104 | 11,113 | 11,119 | 11,120 | 11,108 | 11,093 | 11,078 | 11,064 | 11,041 | 11,007 | 10,966 | 10,929 | 10,898 | 10,881 | 10,866 | 10,856 | 10,845 | 10,832 | 10,813 | 10,791 | 10,769 | 10,745 | 10,724 | 10,708 | 10,691 | 10,698 | 10,735 |
| **Portugal** | 20,295 | 20,301 | 20,312 | 20,324 | 20,321 | 20,317 | 20,350 | 20,420 | 20,488 | 20,563 | 20,593 | 20,556 | 20,486 | 20,405 | 20,322 | 20,278 | 20,267 | 20,253 | 20,242 | 20,224 | 20,190 | 20,112 | 19,986 | 19,844 | 19,736 | 19,682 | 19,725 | 19,783 | 19,793 | 19,786 |
| **Romania** | 11,487 | 11,513 | 11,538 | 11,559 | 11,584 | 11,595 | 11,602 | 11,603 | 11,601 | 11,590 | 11,588 | 11,591 | 11,596 | 11,599 | 11,611 | 11,609 | 11,576 | 11,536 | 11,499 | 11,467 | 11,430 | 11,415 | 11,398 | 11,391 | 11,371 | 11,339 | 11,315 | 11,289 | 11,284 | 11,281 |
| **Slovakia** | 11,528 | 11,547 | 11,567 | 11,587 | 11,600 | 11,604 | 11,600 | 11,588 | 11,574 | 11,542 | 11,529 | 11,516 | 11,501 | 11,483 | 11,467 | 11,451 | 11,434 | 11,408 | 11,380 | 11,357 | 11,333 | 11,320 | 11,313 | 11,310 | 11,298 | 11,287 | 11,270 | 11,252 | 11,234 | 11,220 |
| **Slovenia** | 12,072 | 12,078 | 12,084 | 12,090 | 12,099 | 12,113 | 12,121 | 12,111 | 12,093 | 12,074 | 12,056 | 12,029 | 12,000 | 11,975 | 11,939 | 11,899 | 11,855 | 11,785 | 11,713 | 11,645 | 11,602 | 11,579 | 11,546 | 11,516 | 11,499 | 11,485 | 11,470 | 11,463 | 11,453 | 11,445 |
| **Spain** | 19,490 | 19,400 | 19,328 | 19,262 | 19,200 | 19,145 | 19,100 | 19,035 | 18,962 | 18,921 | 18,898 | 18,934 | 19,015 | 19,132 | 19,262 | 19,374 | 19,553 | 19,843 | 20,171 | 20,448 | 20,615 | 20,692 | 20,748 | 20,785 | 20,817 | 20,845 | 20,905 | 20,962 | 20,831 | 20,530 |
| **Sweden** | 16,444 | 16,500 | 16,535 | 16,556 | 16,577 | 16,582 | 16,563 | 16,528 | 16,494 | 16,455 | 16,430 | 16,433 | 16,450 | 16,494 | 16,548 | 16,600 | 16,637 | 16,656 | 16,655 | 16,651 | 16,645 | 16,635 | 16,625 | 16,607 | 16,591 | 16,570 | 16,548 | 16,532 | 16,560 | 16,633 |
| **Switzerland** | 19,191 | 19,154 | 19,132 | 19,102 | 19,082 | 19,063 | 19,051 | 19,051 | 19,045 | 19,032 | 19,001 | 18,912 | 18,739 | 18,568 | 18,426 | 18,375 | 18,443 | 18,574 | 18,720 | 18,839 | 18,890 | 18,894 | 18,883 | 18,863 | 18,840 | 18,827 | 18,794 | 18,759 | 18,738 | 18,723 |
| **UK** | 16,417 | 16,359 | 16,298 | 16,245 | 16,207 | 16,185 | 16,168 | 16,142 | 16,110 | 16,073 | 16,033 | 15,971 | 15,888 | 15,803 | 15,734 | 15,704 | 15,741 | 15,835 | 15,953 | 16,063 | 16,137 | 16,200 | 16,288 | 16,374 | 16,438 | 16,458 | 16,370 | 16,274 | 16,255 | 16,248 |
| Red gradations = higher prevalence; White gradations = medium prevalence; Green gradations = lower prevalence  UK United Kingdom | | | | | | | | | | | | | | | | | | | | | | | | | | | | | | |

**Supplement Table 6: Prevalence per 100,000 population aged 10-24 years of substance use disorders in 31 European countries from 1990 to 2019**

|  | **1990** | **1991** | **1992** | **1993** | **1994** | **1995** | **1996** | **1997** | **1998** | **1999** | **2000** | **2001** | **2002** | **2003** | **2004** | **2005** | **2006** | **2007** | **2008** | **2009** | **2010** | **2011** | **2012** | **2013** | **2014** | **2015** | **2016** | **2017** | **2018** | **2019** |
| --- | --- | --- | --- | --- | --- | --- | --- | --- | --- | --- | --- | --- | --- | --- | --- | --- | --- | --- | --- | --- | --- | --- | --- | --- | --- | --- | --- | --- | --- | --- |
| **Austria** | 4,156 | 4,137 | 4,096 | 4,033 | 3,966 | 3,909 | 3,907 | 3,963 | 4,040 | 4,115 | 4,155 | 4,195 | 4,258 | 4,325 | 4,387 | 4,428 | 4,435 | 4,435 | 4,451 | 4,478 | 4,503 | 4,511 | 4,464 | 4,380 | 4,305 | 4,275 | 4,249 | 4,205 | 4,203 | 4,239 |
| **Belgium** | 4,432 | 4,544 | 4,646 | 4,726 | 4,775 | 4,784 | 4,747 | 4,696 | 4,637 | 4,589 | 4,566 | 4,501 | 4,368 | 4,212 | 4,080 | 4,017 | 4,002 | 3,989 | 3,984 | 3,977 | 3,980 | 3,992 | 4,002 | 4,021 | 4,017 | 4,005 | 3,984 | 3,948 | 3,893 | 3,808 |
| **Bulgaria** | 2,358 | 2,417 | 2,469 | 2,507 | 2,550 | 2,582 | 2,620 | 2,658 | 2,682 | 2,698 | 2,710 | 2,732 | 2,765 | 2,807 | 2,858 | 2,912 | 2,956 | 3,012 | 3,060 | 3,082 | 3,075 | 3,041 | 2,978 | 2,906 | 2,831 | 2,763 | 2,701 | 2,636 | 2,587 | 2,567 |
| **Croatia** | 2,826 | 2,854 | 2,883 | 2,915 | 2,949 | 2,986 | 3,018 | 3,039 | 3,060 | 3,086 | 3,117 | 3,140 | 3,159 | 3,181 | 3,196 | 3,198 | 3,189 | 3,177 | 3,168 | 3,159 | 3,156 | 3,161 | 3,176 | 3,175 | 3,171 | 3,161 | 3,109 | 3,056 | 3,030 | 2,981 |
| **Cyprus** | 2,832 | 2,767 | 2,708 | 2,664 | 2,634 | 2,626 | 2,631 | 2,649 | 2,681 | 2,717 | 2,753 | 2,797 | 2,836 | 2,881 | 2,926 | 2,966 | 3,004 | 3,037 | 3,065 | 3,088 | 3,108 | 3,129 | 3,138 | 3,134 | 3,117 | 3,089 | 3,049 | 3,006 | 2,994 | 3,015 |
| **Czech Rep** | 3,449 | 3,599 | 3,756 | 3,913 | 4,050 | 4,139 | 4,196 | 4,228 | 4,240 | 4,231 | 4,211 | 4,184 | 4,170 | 4,162 | 4,178 | 4,234 | 4,315 | 4,397 | 4,478 | 4,534 | 4,552 | 4,543 | 4,516 | 4,472 | 4,403 | 4,315 | 4,200 | 4,077 | 3,859 | 3,572 |
| **Denmark** | 3,861 | 3,882 | 3,889 | 3,903 | 3,930 | 3,949 | 3,947 | 3,877 | 3,788 | 3,701 | 3,623 | 3,557 | 3,503 | 3,452 | 3,404 | 3,370 | 3,359 | 3,373 | 3,405 | 3,452 | 3,507 | 3,544 | 3,560 | 3,578 | 3,578 | 3,590 | 3,602 | 3,599 | 3,568 | 3,514 |
| **Estonia** | 2,843 | 2,934 | 3,006 | 3,063 | 3,097 | 3,115 | 3,127 | 3,137 | 3,156 | 3,184 | 3,230 | 3,282 | 3,349 | 3,430 | 3,540 | 3,652 | 3,742 | 3,827 | 3,921 | 3,987 | 4,038 | 4,044 | 3,996 | 3,908 | 3,782 | 3,646 | 3,531 | 3,415 | 3,288 | 3,172 |
| **Finland** | 3,103 | 3,112 | 3,105 | 3,091 | 3,108 | 3,143 | 3,200 | 3,268 | 3,335 | 3,376 | 3,400 | 3,435 | 3,511 | 3,617 | 3,702 | 3,752 | 3,749 | 3,705 | 3,666 | 3,638 | 3,646 | 3,680 | 3,720 | 3,736 | 3,750 | 3,743 | 3,744 | 3,721 | 3,623 | 3,467 |
| **France** | 4,076 | 4,059 | 4,039 | 4,017 | 4,000 | 3,980 | 3,957 | 3,929 | 3,900 | 3,881 | 3,883 | 3,901 | 3,934 | 3,978 | 4,017 | 4,044 | 4,065 | 4,079 | 4,084 | 4,085 | 4,074 | 4,052 | 4,032 | 4,011 | 4,002 | 4,007 | 4,023 | 4,034 | 3,988 | 3,874 |
| **Germany** | 4,246 | 4,268 | 4,257 | 4,226 | 4,173 | 4,136 | 4,088 | 4,071 | 4,069 | 4,065 | 4,070 | 4,083 | 4,079 | 4,077 | 4,082 | 4,105 | 4,127 | 4,139 | 4,164 | 4,177 | 4,191 | 4,135 | 4,001 | 3,832 | 3,691 | 3,630 | 3,653 | 3,679 | 3,672 | 3,652 |
| **Greece** | 2,709 | 2,767 | 2,822 | 2,858 | 2,893 | 2,930 | 2,953 | 2,952 | 2,942 | 2,927 | 2,935 | 2,957 | 2,978 | 2,997 | 3,018 | 3,020 | 3,025 | 3,039 | 3,049 | 3,058 | 3,049 | 3,017 | 2,962 | 2,887 | 2,813 | 2,753 | 2,692 | 2,646 | 2,642 | 2,669 |
| **Hungary** | 2,431 | 2,492 | 2,542 | 2,595 | 2,657 | 2,725 | 2,770 | 2,797 | 2,798 | 2,781 | 2,753 | 2,735 | 2,722 | 2,713 | 2,707 | 2,711 | 2,732 | 2,771 | 2,814 | 2,854 | 2,882 | 2,889 | 2,871 | 2,842 | 2,806 | 2,778 | 2,748 | 2,715 | 2,660 | 2,584 |
| **Iceland** | 3,296 | 3,268 | 3,257 | 3,254 | 3,266 | 3,286 | 3,324 | 3,340 | 3,308 | 3,261 | 3,263 | 3,248 | 3,237 | 3,250 | 3,262 | 3,258 | 3,267 | 3,271 | 3,300 | 3,338 | 3,363 | 3,401 | 3,444 | 3,486 | 3,505 | 3,517 | 3,503 | 3,488 | 3,460 | 3,434 |
| **Ireland** | 4,586 | 4,571 | 4,582 | 4,608 | 4,649 | 4,710 | 4,760 | 4,801 | 4,853 | 4,917 | 5,002 | 5,107 | 5,225 | 5,339 | 5,426 | 5,452 | 5,444 | 5,378 | 5,286 | 5,187 | 5,103 | 5,028 | 4,953 | 4,885 | 4,829 | 4,790 | 4,753 | 4,737 | 4,678 | 4,561 |
| **Italy** | 4,246 | 4,309 | 4,354 | 4,376 | 4,376 | 4,347 | 4,280 | 4,179 | 4,064 | 3,957 | 3,880 | 3,846 | 3,838 | 3,840 | 3,850 | 3,845 | 3,838 | 3,827 | 3,819 | 3,812 | 3,795 | 3,738 | 3,637 | 3,524 | 3,410 | 3,335 | 3,316 | 3,313 | 3,271 | 3,195 |
| **Latvia** | 3,293 | 3,393 | 3,467 | 3,500 | 3,500 | 3,479 | 3,426 | 3,344 | 3,261 | 3,203 | 3,171 | 3,169 | 3,195 | 3,243 | 3,317 | 3,398 | 3,465 | 3,512 | 3,531 | 3,521 | 3,504 | 3,481 | 3,434 | 3,372 | 3,284 | 3,201 | 3,158 | 3,089 | 2,932 | 2,763 |
| **Lithuania** | 2,282 | 2,350 | 2,404 | 2,431 | 2,430 | 2,404 | 2,361 | 2,299 | 2,239 | 2,194 | 2,159 | 2,118 | 2,064 | 2,018 | 1,997 | 2,010 | 2,056 | 2,113 | 2,169 | 2,208 | 2,242 | 2,280 | 2,317 | 2,354 | 2,383 | 2,391 | 2,417 | 2,423 | 2,366 | 2,274 |
| **Luxembourg** | 4,175 | 4,213 | 4,240 | 4,246 | 4,233 | 4,210 | 4,201 | 4,190 | 4,182 | 4,174 | 4,163 | 4,118 | 4,065 | 3,996 | 3,971 | 3,966 | 3,973 | 3,962 | 3,979 | 4,018 | 4,033 | 4,047 | 4,070 | 4,077 | 4,065 | 4,074 | 4,096 | 4,135 | 4,155 | 4,166 |
| **Malta** | 2,721 | 2,750 | 2,786 | 2,820 | 2,870 | 2,917 | 2,975 | 3,053 | 3,133 | 3,192 | 3,219 | 3,228 | 3,251 | 3,279 | 3,311 | 3,346 | 3,388 | 3,419 | 3,462 | 3,510 | 3,554 | 3,587 | 3,592 | 3,562 | 3,512 | 3,479 | 3,550 | 3,609 | 3,554 | 3,446 |
| **Netherlands** | 3,988 | 3,958 | 3,928 | 3,893 | 3,848 | 3,786 | 3,725 | 3,648 | 3,569 | 3,506 | 3,468 | 3,443 | 3,418 | 3,396 | 3,379 | 3,376 | 3,385 | 3,386 | 3,384 | 3,382 | 3,382 | 3,383 | 3,388 | 3,394 | 3,411 | 3,432 | 3,412 | 3,370 | 3,379 | 3,435 |
| **Norway** | 2,980 | 2,945 | 2,908 | 2,874 | 2,849 | 2,836 | 2,830 | 2,804 | 2,766 | 2,726 | 2,684 | 2,636 | 2,582 | 2,540 | 2,506 | 2,493 | 2,492 | 2,493 | 2,490 | 2,504 | 2,527 | 2,554 | 2,571 | 2,579 | 2,566 | 2,551 | 2,517 | 2,483 | 2,485 | 2,509 |
| **Poland** | 2,509 | 2,521 | 2,536 | 2,554 | 2,576 | 2,606 | 2,641 | 2,683 | 2,728 | 2,776 | 2,819 | 2,858 | 2,897 | 2,941 | 2,986 | 3,029 | 3,071 | 3,106 | 3,130 | 3,146 | 3,154 | 3,160 | 3,171 | 3,173 | 3,163 | 3,133 | 3,052 | 2,963 | 2,862 | 2,725 |
| **Portugal** | 3,994 | 4,012 | 4,041 | 4,071 | 4,104 | 4,146 | 4,198 | 4,250 | 4,289 | 4,307 | 4,312 | 4,288 | 4,245 | 4,203 | 4,170 | 4,131 | 4,080 | 4,023 | 3,956 | 3,893 | 3,834 | 3,802 | 3,801 | 3,809 | 3,822 | 3,828 | 3,843 | 3,855 | 3,817 | 3,707 |
| **Romania** | 1,979 | 2,032 | 2,062 | 2,039 | 2,026 | 2,032 | 2,042 | 2,045 | 2,035 | 2,017 | 2,005 | 2,003 | 1,994 | 1,978 | 1,970 | 1,978 | 1,988 | 2,012 | 2,041 | 2,062 | 2,055 | 2,019 | 1,964 | 1,906 | 1,853 | 1,805 | 1,774 | 1,758 | 1,768 | 1,804 |
| **Slovakia** | 2,570 | 2,611 | 2,663 | 2,730 | 2,788 | 2,837 | 2,884 | 2,923 | 2,967 | 2,998 | 3,018 | 3,026 | 3,019 | 3,010 | 3,008 | 3,032 | 3,068 | 3,116 | 3,157 | 3,186 | 3,196 | 3,193 | 3,180 | 3,153 | 3,116 | 3,079 | 3,064 | 3,049 | 2,969 | 2,849 |
| **Slovenia** | 3,217 | 3,210 | 3,215 | 3,226 | 3,252 | 3,279 | 3,297 | 3,306 | 3,308 | 3,314 | 3,329 | 3,363 | 3,390 | 3,422 | 3,449 | 3,466 | 3,485 | 3,505 | 3,532 | 3,555 | 3,560 | 3,539 | 3,511 | 3,466 | 3,416 | 3,375 | 3,338 | 3,297 | 3,185 | 2,975 |
| **Spain** | 4,479 | 4,613 | 4,753 | 4,896 | 5,034 | 5,160 | 5,281 | 5,404 | 5,519 | 5,610 | 5,681 | 5,759 | 5,858 | 5,942 | 6,005 | 6,024 | 5,928 | 5,730 | 5,499 | 5,286 | 5,146 | 5,073 | 5,036 | 5,021 | 5,000 | 4,979 | 4,945 | 4,890 | 4,830 | 4,788 |
| **Sweden** | 3,200 | 3,242 | 3,268 | 3,289 | 3,311 | 3,304 | 3,243 | 3,155 | 3,044 | 2,939 | 2,856 | 2,792 | 2,752 | 2,741 | 2,744 | 2,784 | 2,857 | 2,944 | 3,048 | 3,151 | 3,248 | 3,339 | 3,436 | 3,504 | 3,532 | 3,497 | 3,178 | 2,862 | 2,800 | 2,768 |
| **Switzerland** | 6,268 | 6,259 | 6,228 | 6,174 | 6,107 | 6,039 | 5,937 | 5,822 | 5,704 | 5,615 | 5,576 | 5,594 | 5,648 | 5,730 | 5,808 | 5,845 | 5,833 | 5,784 | 5,722 | 5,663 | 5,643 | 5,670 | 5,689 | 5,698 | 5,695 | 5,689 | 5,657 | 5,612 | 5,402 | 4,986 |
| **UK** | 5,964 | 5,964 | 5,951 | 5,925 | 5,890 | 5,838 | 5,756 | 5,653 | 5,563 | 5,514 | 5,504 | 5,522 | 5,559 | 5,610 | 5,655 | 5,701 | 5,767 | 5,854 | 5,947 | 6,033 | 6,098 | 6,163 | 6,234 | 6,286 | 6,302 | 6,270 | 6,121 | 5,954 | 5,818 | 5,643 |
| Red gradations = higher prevalence; White gradations = medium prevalence; Green gradations = lower prevalence  UK United Kingdom | | | | | | | | | | | | | | | | | | | | | | | | | | | | | | |

**Supplement Table 7: Incidence per 100,000 population aged 10-24 years of self-harm in 31 European countries from 1990 to 2019**

|  | **1990** | **1991** | **1992** | **1993** | **1994** | **1995** | **1996** | **1997** | **1998** | **1999** | **2000** | **2001** | **2002** | **2003** | **2004** | **2005** | **2006** | **2007** | **2008** | **2009** | **2010** | **2011** | **2012** | **2013** | **2014** | **2015** | **2016** | **2017** | **2018** | **2019** |
| --- | --- | --- | --- | --- | --- | --- | --- | --- | --- | --- | --- | --- | --- | --- | --- | --- | --- | --- | --- | --- | --- | --- | --- | --- | --- | --- | --- | --- | --- | --- |
| **Austria** | 186∙4 | 186∙2 | 184∙8 | 182∙6 | 180∙2 | 178∙1 | 176∙1 | 173∙2 | 169∙8 | 166∙5 | 163∙6 | 161∙2 | 158∙7 | 156∙3 | 153∙8 | 151∙1 | 147∙3 | 142∙7 | 138∙3 | 134∙4 | 131∙8 | 130∙5 | 129∙6 | 128∙5 | 127∙2 | 125∙5 | 121∙9 | 119∙3 | 119∙1 | 119∙9 |
| **Belgium** | 170∙0 | 171∙6 | 173∙2 | 174∙6 | 175∙5 | 175∙7 | 175∙4 | 175∙0 | 174∙6 | 174∙3 | 174∙2 | 174∙3 | 174∙4 | 174∙6 | 174∙7 | 174∙7 | 172∙6 | 168∙1 | 162∙7 | 157∙9 | 155∙2 | 154∙4 | 153∙9 | 153∙5 | 152∙6 | 151∙2 | 146∙5 | 141∙4 | 138∙6 | 135∙6 |
| **Bulgaria** | 164∙1 | 164∙4 | 164∙3 | 163∙5 | 162∙5 | 161∙3 | 159∙8 | 157∙8 | 155∙3 | 152∙4 | 149∙6 | 146∙3 | 142∙1 | 137∙6 | 133∙2 | 129∙3 | 125∙3 | 120∙8 | 116∙3 | 112∙0 | 108∙9 | 106∙7 | 104∙7 | 102∙7 | 101∙1 | 99∙5 | 97∙3 | 95∙5 | 95∙0 | 95∙4 |
| **Croatia** | 163∙6 | 162∙5 | 161∙5 | 160∙8 | 160∙3 | 160∙1 | 159∙9 | 159∙1 | 158∙2 | 157∙0 | 155∙6 | 152∙5 | 147∙6 | 141∙9 | 136∙3 | 131∙6 | 127∙6 | 123∙6 | 119∙9 | 116∙8 | 114∙7 | 113∙4 | 112∙6 | 111∙4 | 109∙7 | 107∙4 | 101∙3 | 96∙1 | 95∙5 | 96∙1 |
| **Cyprus** | 59∙3 | 59∙3 | 59∙3 | 59∙5 | 59∙7 | 60∙0 | 60∙4 | 60∙9 | 61∙5 | 62∙1 | 62∙6 | 63∙0 | 63∙4 | 63∙7 | 64∙0 | 64∙4 | 64∙9 | 65∙2 | 65∙4 | 65∙6 | 65∙7 | 65∙6 | 65∙4 | 65∙0 | 64∙7 | 64∙4 | 65∙0 | 65∙5 | 65∙1 | 64∙6 |
| **Czech Rep** | 146∙4 | 150∙0 | 153∙5 | 157∙0 | 159∙8 | 161∙7 | 162∙0 | 160∙9 | 158∙7 | 156∙3 | 154∙2 | 152∙7 | 151∙5 | 150∙6 | 150∙1 | 150∙3 | 150∙2 | 149∙5 | 148∙5 | 147∙2 | 145∙9 | 144∙7 | 143∙5 | 142∙1 | 140∙4 | 138∙2 | 135∙5 | 132∙6 | 127∙7 | 120∙7 |
| **Denmark** | 124∙6 | 124∙8 | 124∙6 | 124∙3 | 124∙2 | 123∙7 | 122∙0 | 117∙8 | 112∙6 | 107∙4 | 102∙6 | 98∙3 | 94∙4 | 90∙7 | 87∙4 | 84∙6 | 82∙0 | 79∙5 | 77∙2 | 75∙3 | 74∙2 | 73∙7 | 73∙2 | 72∙9 | 72∙3 | 71∙5 | 70∙2 | 69∙3 | 69∙5 | 70∙0 |
| **Estonia** | 225∙0 | 222∙6 | 220∙2 | 217∙8 | 215∙2 | 213∙0 | 211∙7 | 211∙3 | 211∙3 | 211∙4 | 211∙0 | 207∙7 | 201∙9 | 195∙0 | 189∙2 | 185∙1 | 181∙6 | 177∙4 | 173∙4 | 168∙8 | 163∙9 | 158∙4 | 152∙3 | 145∙7 | 139∙2 | 133∙5 | 129∙1 | 126∙3 | 124∙7 | 124∙6 |
| **Finland** | 266∙2 | 265∙8 | 263∙8 | 261∙1 | 259∙9 | 259∙6 | 259∙3 | 258∙7 | 257∙2 | 254∙4 | 251∙4 | 249∙3 | 248∙4 | 248∙9 | 249∙0 | 248∙3 | 245∙5 | 240∙7 | 235∙5 | 230∙8 | 227∙8 | 226∙1 | 224∙7 | 222∙0 | 218∙9 | 214∙6 | 202∙1 | 190∙7 | 187∙9 | 185∙8 |
| **France** | 142∙8 | 142∙8 | 142∙7 | 142∙5 | 142∙0 | 141∙1 | 139∙5 | 136∙8 | 133∙7 | 131∙0 | 129∙0 | 127∙4 | 126∙0 | 124∙5 | 122∙8 | 120∙9 | 118∙6 | 115∙7 | 112∙5 | 109∙4 | 107∙0 | 105∙2 | 103∙8 | 102∙3 | 100∙8 | 98∙9 | 94∙1 | 90∙3 | 90∙3 | 90∙8 |
| **Germany** | 128∙5 | 128∙6 | 127∙9 | 126∙6 | 125∙2 | 124∙0 | 122∙4 | 120∙5 | 118∙5 | 116∙6 | 115∙2 | 114∙5 | 113∙9 | 113∙4 | 112∙7 | 112∙0 | 110∙1 | 107∙1 | 104∙1 | 101∙5 | 100∙2 | 100∙0 | 100∙3 | 100∙6 | 100∙7 | 100∙6 | 99∙6 | 98∙7 | 98∙7 | 99∙2 |
| **Greece** | 52∙9 | 52∙5 | 52∙0 | 51∙6 | 51∙3 | 51∙1 | 51∙0 | 50∙8 | 50∙6 | 50∙3 | 50∙3 | 50∙4 | 50∙7 | 51∙1 | 51∙3 | 51∙4 | 51∙3 | 51∙0 | 50∙6 | 50∙3 | 50∙1 | 49∙9 | 49∙7 | 49∙4 | 49∙0 | 48∙8 | 50∙1 | 51∙5 | 51∙6 | 51∙6 |
| **Hungary** | 155∙6 | 157∙8 | 159∙5 | 160∙8 | 161∙9 | 162∙7 | 161∙5 | 157∙4 | 151∙5 | 145∙1 | 139∙8 | 135∙7 | 131∙7 | 128∙1 | 124∙9 | 122∙4 | 120∙5 | 118∙8 | 117∙3 | 116∙0 | 114∙7 | 113∙3 | 111∙7 | 110∙1 | 108∙6 | 107∙1 | 105∙3 | 103∙8 | 103∙2 | 103∙4 |
| **Iceland** | 130∙9 | 130∙3 | 129∙8 | 129∙1 | 128∙8 | 129∙0 | 129∙4 | 128∙0 | 125∙0 | 121∙3 | 118∙8 | 116∙0 | 113∙2 | 111∙0 | 109∙1 | 106∙8 | 104∙3 | 100∙9 | 97∙9 | 95∙5 | 94∙1 | 93∙5 | 93∙2 | 93∙1 | 92∙6 | 92∙1 | 90∙9 | 89∙8 | 89∙1 | 88∙6 |
| **Ireland** | 127∙5 | 132∙0 | 136∙8 | 142∙1 | 147∙7 | 153∙6 | 159∙5 | 165∙3 | 171∙1 | 176∙5 | 181∙4 | 185∙8 | 190∙3 | 194∙2 | 196∙8 | 197∙5 | 195∙1 | 189∙0 | 180∙7 | 172∙3 | 165∙4 | 160∙3 | 156∙0 | 152∙0 | 147∙2 | 141∙3 | 125∙8 | 113∙7 | 113∙5 | 114∙2 |
| **Italy** | 60∙2 | 61∙9 | 63∙3 | 64∙3 | 65∙0 | 65∙2 | 64∙9 | 64∙2 | 63∙2 | 61∙8 | 60∙4 | 58∙4 | 55∙8 | 53∙0 | 50∙3 | 48∙0 | 46∙1 | 43∙9 | 42∙0 | 40∙4 | 39∙5 | 39∙1 | 38∙8 | 38∙6 | 38∙3 | 37∙7 | 35∙7 | 33∙9 | 34∙6 | 36∙9 |
| **Latvia** | 184∙5 | 181∙7 | 178∙8 | 175∙6 | 172∙4 | 169∙7 | 168∙0 | 167∙3 | 167∙3 | 167∙7 | 167∙4 | 165∙7 | 163∙1 | 160∙2 | 157∙9 | 156∙2 | 154∙9 | 152∙9 | 150∙4 | 147∙2 | 144∙3 | 141∙4 | 138∙2 | 134∙7 | 130∙6 | 126∙8 | 121∙3 | 116∙4 | 114∙3 | 114∙5 |
| **Lithuania** | 227∙8 | 228∙7 | 230∙4 | 232∙2 | 233∙8 | 235∙3 | 238∙8 | 245∙7 | 253∙9 | 261∙5 | 264∙7 | 263∙8 | 261∙6 | 259∙2 | 257∙6 | 257∙5 | 257∙9 | 257∙3 | 255∙3 | 251∙4 | 247∙6 | 245∙0 | 242∙6 | 239∙9 | 236∙1 | 230∙2 | 216∙5 | 205∙3 | 203∙7 | 207∙1 |
| **Luxembourg** | 150∙3 | 149∙7 | 148∙6 | 147∙0 | 144∙8 | 142∙5 | 140∙2 | 137∙5 | 134∙6 | 131∙6 | 128∙5 | 124∙8 | 120∙7 | 116∙1 | 112∙2 | 108∙7 | 105∙3 | 101∙4 | 97∙9 | 95∙4 | 93∙8 | 93∙1 | 92∙9 | 92∙8 | 92∙5 | 92∙3 | 93∙1 | 94∙5 | 94∙8 | 94∙9 |
| **Malta** | 53∙7 | 54∙1 | 54∙5 | 55∙0 | 55∙6 | 56∙4 | 57∙2 | 58∙3 | 59∙4 | 60∙2 | 60∙7 | 61∙2 | 62∙0 | 63∙2 | 64∙2 | 64∙9 | 65∙0 | 64∙8 | 64∙4 | 64∙1 | 64∙0 | 64∙2 | 64∙3 | 64∙2 | 63∙9 | 63∙6 | 64∙6 | 65∙6 | 64∙5 | 62∙0 |
| **Netherlands** | 116∙7 | 119∙0 | 121∙2 | 123∙2 | 124∙7 | 125∙6 | 126∙4 | 126∙9 | 127∙3 | 127∙7 | 128∙0 | 128∙3 | 128∙8 | 129∙2 | 129∙3 | 129∙2 | 128∙9 | 128∙3 | 127∙4 | 126∙1 | 124∙6 | 122∙8 | 120∙6 | 118∙0 | 115∙1 | 111∙9 | 107∙1 | 102∙7 | 100∙2 | 98∙3 |
| **Norway** | 201∙8 | 204∙2 | 205∙3 | 205∙2 | 204∙7 | 203∙5 | 200∙2 | 193∙4 | 185∙2 | 177∙1 | 171∙1 | 167∙2 | 164∙2 | 162∙1 | 160∙3 | 158∙6 | 156∙3 | 153∙2 | 150∙0 | 147∙5 | 145∙9 | 145∙5 | 145∙6 | 145∙6 | 144∙5 | 142∙6 | 135∙8 | 129∙7 | 129∙2 | 129∙7 |
| **Poland** | 111∙7 | 113∙1 | 114∙5 | 115∙9 | 117∙5 | 119∙2 | 121∙2 | 123∙5 | 126∙0 | 128∙3 | 130∙3 | 131∙8 | 133∙1 | 134∙4 | 135∙5 | 136∙5 | 137∙2 | 137∙5 | 137∙4 | 137∙0 | 136∙6 | 136∙2 | 135∙7 | 134∙9 | 133∙7 | 132∙1 | 128∙0 | 124∙0 | 122∙3 | 121∙3 |
| **Portugal** | 118∙3 | 119∙4 | 120∙5 | 121∙3 | 121∙7 | 122∙1 | 120∙7 | 116∙5 | 110∙6 | 104∙5 | 99∙6 | 95∙8 | 92∙1 | 88∙8 | 85∙9 | 83∙2 | 80∙6 | 77∙9 | 75∙4 | 73∙2 | 71∙4 | 70∙2 | 69∙4 | 68∙8 | 68∙1 | 67∙3 | 65∙2 | 63∙5 | 63∙7 | 64∙5 |
| **Romania** | 80∙9 | 81∙5 | 82∙1 | 82∙3 | 82∙7 | 83∙1 | 83∙7 | 84∙2 | 84∙5 | 85∙0 | 85∙7 | 86∙8 | 88∙5 | 90∙2 | 91∙9 | 93∙1 | 93∙7 | 94∙1 | 94∙4 | 94∙5 | 94∙4 | 94∙2 | 93∙7 | 93∙2 | 92∙5 | 91∙7 | 90∙1 | 88∙7 | 88∙7 | 89∙5 |
| **Slovakia** | 107∙0 | 107∙6 | 108∙3 | 109∙1 | 109∙9 | 110∙5 | 110∙5 | 110∙0 | 109∙2 | 108∙4 | 107∙8 | 107∙4 | 106∙8 | 106∙1 | 105∙8 | 106∙0 | 106∙6 | 107∙1 | 107∙6 | 107∙9 | 108∙0 | 107∙9 | 107∙6 | 107∙2 | 106∙4 | 105∙4 | 105∙1 | 104∙7 | 103∙9 | 103∙6 |
| **Slovenia** | 191∙3 | 193∙8 | 196∙4 | 199∙0 | 201∙5 | 203∙6 | 205∙0 | 205∙7 | 205∙4 | 204∙8 | 204∙3 | 203∙4 | 201∙9 | 200∙1 | 198∙0 | 195∙4 | 191∙5 | 185∙7 | 179∙0 | 172∙6 | 167∙1 | 161∙9 | 156∙3 | 150∙6 | 146∙0 | 143∙1 | 141∙5 | 140∙2 | 138∙0 | 134∙4 |
| **Spain** | 67∙7 | 68∙5 | 69∙4 | 70∙1 | 70∙9 | 71∙7 | 71∙9 | 71∙4 | 70∙4 | 69∙1 | 68∙0 | 67∙1 | 66∙1 | 65∙0 | 63∙8 | 62∙8 | 61∙5 | 60∙0 | 58∙6 | 57∙3 | 56∙2 | 55∙3 | 54∙4 | 53∙7 | 53∙0 | 52∙3 | 52∙1 | 52∙2 | 52∙1 | 52∙2 |
| **Sweden** | 173∙9 | 175∙3 | 176∙0 | 176∙4 | 176∙8 | 176∙1 | 173∙2 | 168∙7 | 163∙3 | 158∙5 | 155∙4 | 154∙2 | 154∙2 | 155∙4 | 157∙3 | 160∙0 | 163∙0 | 166∙1 | 168∙9 | 171∙4 | 173∙1 | 173∙9 | 173∙7 | 172∙4 | 170∙4 | 167∙3 | 159∙6 | 152∙4 | 150∙0 | 148∙4 |
| **Switzerland** | 251∙6 | 249∙4 | 246∙6 | 243∙4 | 240∙2 | 237∙3 | 233∙9 | 230∙1 | 225∙8 | 221∙7 | 218∙1 | 214∙8 | 211∙3 | 208∙0 | 204∙4 | 200∙4 | 195∙3 | 189∙0 | 181∙9 | 174∙4 | 167∙4 | 160∙9 | 153∙9 | 146∙6 | 139∙5 | 133∙1 | 126∙0 | 120∙7 | 118∙0 | 116∙0 |
| **UK** | 112∙9 | 113∙1 | 112∙8 | 112∙2 | 111∙4 | 110∙3 | 108∙4 | 105∙6 | 102∙8 | 100∙6 | 99∙1 | 98∙0 | 97∙0 | 96∙2 | 95∙1 | 94∙0 | 92∙6 | 90∙6 | 88∙5 | 86∙7 | 85∙6 | 85∙3 | 85∙0 | 84∙7 | 84∙2 | 83∙2 | 81∙1 | 79∙3 | 78∙8 | 78∙6 |
| Red gradations = higher incidence; White gradations = medium incidence; Green gradations = lower incidence  UK United Kingdom | | | | | | | | | | | | | | | | | | | | | | | | | | | | | | |

**Supplement Table 8: Ranking of Years Lived with Disability (YLDs) for the first 22 all-causes of diseases at level 2 in European Union, Iceland, Norway and Switzerland, year 2019, both sexes, age 10-24**

**
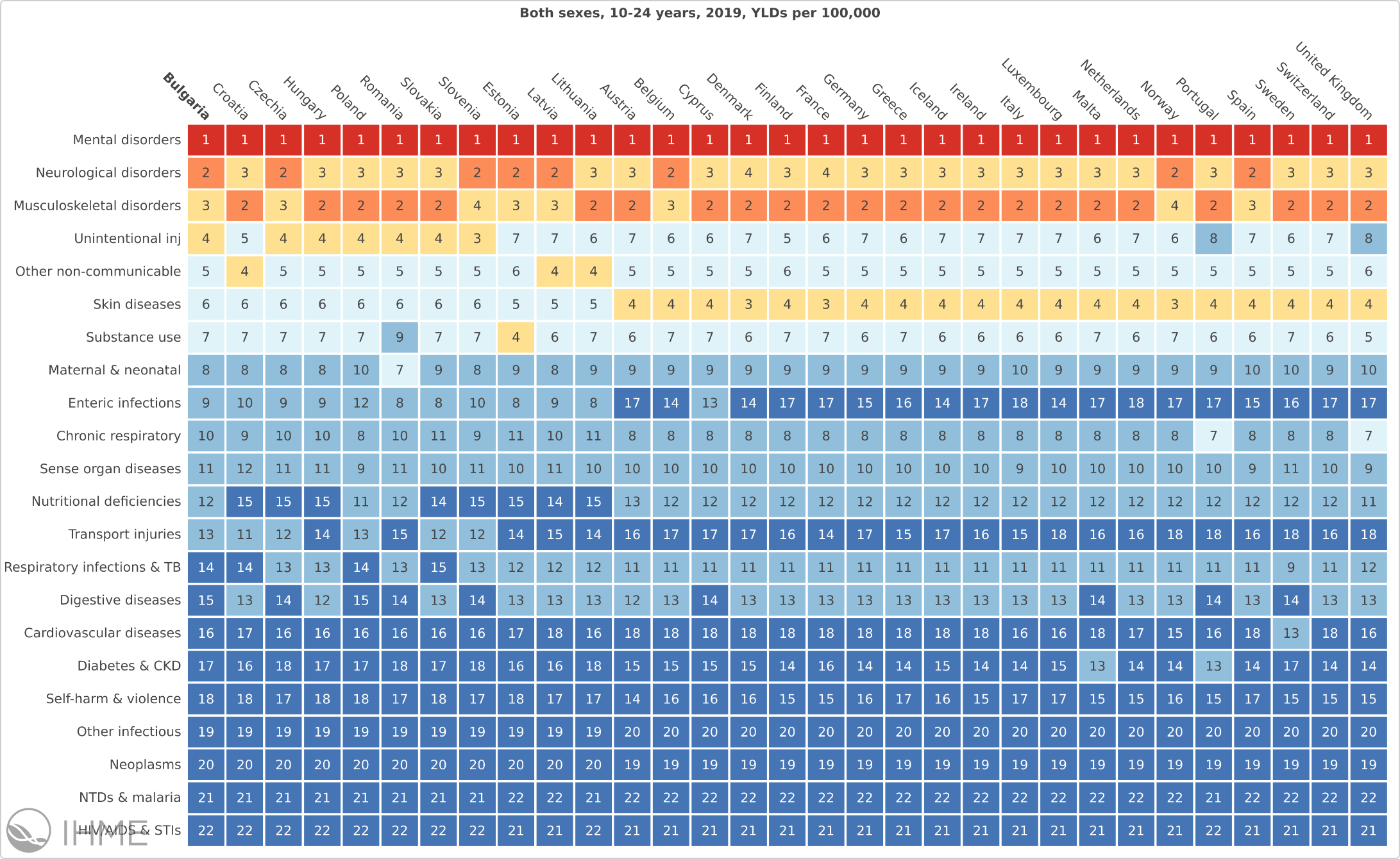
**

**Supplement Table 9: Ranking of Years Lived with Disability (YLDs) for the first 25 all-causes of diseases at level 3 in European Union, Iceland, Norway and Switzerland, year 2019, both sexes, age 10-24**

**
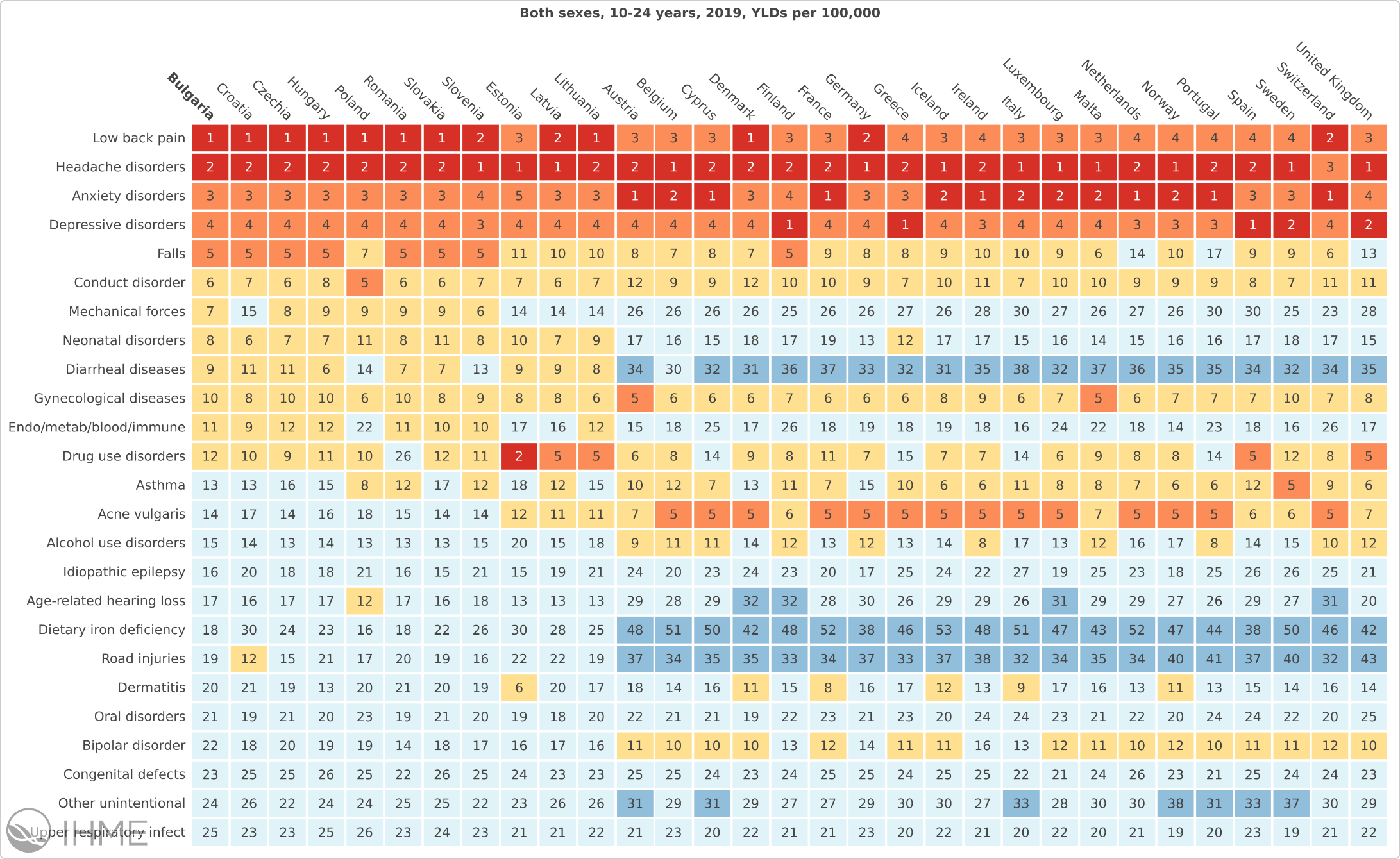
**

**Supplement Table 10: Ranking of Years of Life Lost (YLLs) for the first 25 all-causes of diseases at level 3 in European Union, Iceland, Norway and Switzerland, year 2019, both sexes, age 10-24**

**
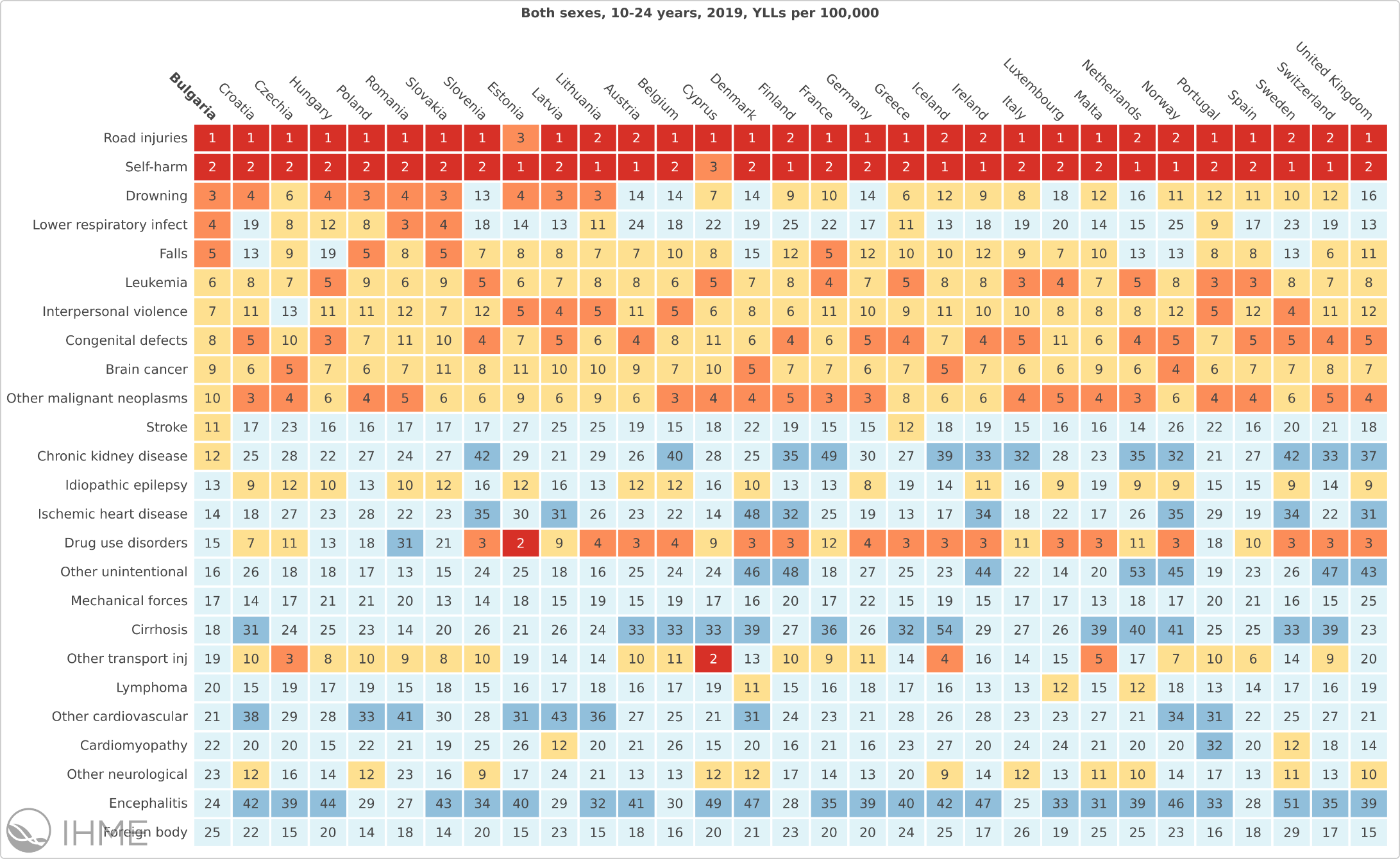
**
