# Supplementary material for: The burden of mental disorders, substance use disorders and self-harm among young people in Europe, 1990–2019: Findings from the Global Burden of Disease Study 2019
Source: Lancet Reg Health Eur. 2022 Apr 1;16:100341. doi: 10.1016/j.lanepe.2022.100341 (PMC8980870; doi:10.1016/j.lanepe.2022.100341)
Supplement: Supplementary file 3 [file mmc3.docx]

**Appendix - Data coverage of mental and substance use disorders and self-harm among young people in Europe**

**Summary**

**Aim:** Gain an overview over existing data-sources for mental and substance use disorders in the population aged 10 to 24 in the European Union, Iceland, Norway, Switzerland, and United Kingdom employed in the latest iteration of the GBD study (GBD 2019).

**Methods:** Lists over GBD 2019 input data-sources for mental and substance use disorders disorders from the European Union, Iceland, Norway, Switzerland, and United Kingdom were retrieved from GHDx, and sorted by country-sex-age group. References were inserted in a table by 5-year span age-groups (10-14, 15-19 and 20-24). A reference was included in an age-group if at least one of the age-years in the group were endorsed in the reference.

**Results:** A total of 528 unique data-sources (references) for mental and substance use disorders, and self-harm, in the ages 10-24 were listed as included in GBD 2019 for countries in European Union, Iceland, Norway, Switzerland and United Kingdom, with an average of 29.7 per country. Table 1 gives an overview over number of data-sources that were included from each country. Drug use disorders was the cause with the highest number of data-sources, with an average of 16.1 references per country, followed by depressive disorders (3.8), eating disorders (2.0) and schizophrenia (1.6). None of the countries had any data-sources for Idiopathic developmental intellectual disability. Apart from this cause, only two countries (Netherlands and United Kingdom) were listed with at least one data-source for all other causes. There was large discrepancies between number of data-sources from Western-European, Central-European and Eastern-European countries. While Western-Europe had on average 36.3 data-sources per country, the corresponding numbers were 18.5 and 18.3 for Central-Europe and Eastern-Europe, respectively. Table 2 gives an overview over each reference by cause-country-sex-age-group. Data-sources were also scarce for the age-group 10-14 compared to the other age-groups.

**Conclusion:** There is still a lack of prevalence data on mental disorders among adolescents and young adults in Europe, in particular from countries in Central and Eastern Europe. Data is very scarce for the age-group 10 to 14 years. With the widespread concern around a potential increase in mental health problems among younger persons, updated high-quality data-collection from this population group should be a priority in several European countries.

Table 1. Number of data-sources for mental disorders among males and females aged 10-24 per country in the European Union, Iceland, Norway, Switzerland and United Kingdom.

|  | **Countries** | | | | | | | | | | | | | | | | | | | | | | | | | | | | | |  |
| --- | --- | --- | --- | --- | --- | --- | --- | --- | --- | --- | --- | --- | --- | --- | --- | --- | --- | --- | --- | --- | --- | --- | --- | --- | --- | --- | --- | --- | --- | --- | --- |
| **Causes** | **AU** | **BE** | **BU** | **CR** | **CY** | **CZ** | **DE** | **ES** | **FIN** | **FR** | **GE** | **GR** | **HU** | **ICE** | **IRE** | **ITA** | **LA** | **LIT** | **LU** | **NE** | **NO** | **PO** | **PT** | **RO** | **SL** | **SV** | **SP** | **SW** | **SZ** | **UK** | **Avg** |
| **SCZ** | 0 | 0 | 1 | 0 | 0 | 1 | 6 | 0 | 3 | 3 | 3 | 0 | 0 | 0 | 5 | 0 | 0 | 0 | 0 | 4 | 2 | 0 | 0 | 0 | 0 | 0 | 2 | 6 | 0 | 13 | 1.6 |
| **DEP** | 2 | 4 | 1 | 1 | 2 | 2 | 3 | 2 | 5 | 6 | 10 | 5 | 1 | 0 | 5 | 2 | 3 | 0 | 1 | 10 | 10 | 1 | 3 | 1 | 1 | 1 | 10 | 5 | 2 | 14 | 3.8 |
| **BIP** | 1 | 0 | 1 | 0 | 0 | 0 | 3 | 0 | 2 | 1 | 4 | 0 | 0 | 1 | 4 | 1 | 0 | 0 | 0 | 3 | 1 | 1 | 1 | 1 | 0 | 0 | 3 | 2 | 0 | 5 | 1.2 |
| **ANX** | 1 | 1 | 1 | 0 | 0 | 0 | 1 | 0 | 1 | 1 | 6 | 1 | 0 | 0 | 3 | 1 | 0 | 1 | 0 | 9 | 1 | 0 | 1 | 1 | 0 | 0 | 5 | 0 | 1 | 5 | 1.4 |
| **EATING** | 1 | 1 | 0 | 0 | 0 | 0 | 3 | 0 | 5 | 5 | 6 | 1 | 2 | 0 | 1 | 1 | 0 | 0 | 0 | 5 | 1 | 0 | 3 | 1 | 0 | 0 | 8 | 6 | 2 | 7 | 2.0 |
| **ASD** | 0 | 0 | 0 | 0 | 0 | 0 | 8 | 0 | 1 | 2 | 0 | 0 | 0 | 3 | 1 | 0 | 0 | 1 | 0 | 1 | 2 | 0 | 0 | 0 | 0 | 0 | 0 | 6 | 0 | 9 | 1.1 |
| **ADHD** | 1 | 0 | 0 | 0 | 1 | 0 | 1 | 0 | 1 | 1 | 4 | 1 | 1 | 0 | 0 | 1 | 0 | 0 | 0 | 1 | 0 | 0 | 0 | 0 | 0 | 0 | 7 | 2 | 1 | 6 | 1.0 |
| **CONDUCT** | 1 | 0 | 0 | 0 | 0 | 0 | 1 | 0 | 0 | 2 | 1 | 0 | 0 | 0 | 0 | 0 | 0 | 0 | 0 | 1 | 0 | 0 | 0 | 0 | 0 | 0 | 0 | 0 | 0 | 2 | 0.3 |
| **ID** | 0 | 0 | 0 | 0 | 0 | 0 | 0 | 0 | 0 | 0 | 0 | 0 | 0 | 0 | 0 | 0 | 0 | 0 | 0 | 0 | 0 | 0 | 0 | 0 | 0 | 0 | 0 | 0 | 0 | 0 | 0.0 |
| **AUD** | 1 | 2 | 0 | 2 | 0 | 0 | 0 | 1 | 4 | 2 | 10 | 0 | 0 | 1 | 1 | 0 | 0 | 0 | 0 | 5 | 2 | 1 | 1 | 1 | 0 | 0 | 2 | 17 | 3 | 7 | 2.1 |
| **DUD** | 14 | 9 | 11 | 12 | 8 | 20 | 14 | 15 | 15 | 15 | 19 | 15 | 13 | 7 | 18 | 21 | 14 | 14 | 8 | 26 | 13 | 14 | 11 | 9 | 16 | 14 | 38 | 16 | 9 | 56 | 16.1 |
| **SH** | 1 | 2 | 1 | 2 | 2 | 3 | 2 | 1 | 2 | 1 | 1 | 1 | 1 | 3 | 1 | 1 | 2 | 2 | 2 | 2 | 1 | 2 | 2 | 2 | 2 | 2 | 2 | 2 | 2 | 4 | 1.8 |
| **All causes^1^** | 19 | 21 | 16 | 17 | 13 | 26 | 42 | 19 | 36 | 39 | 50 | 22 | 18 | 15 | 35 | 27 | 19 | 17 | 11 | 51 | 30 | 19 | 21 | 16 | 19 | 17 | 69 | 59 | 18 | 111 | 29.7 |

^1^Number of unique references per country

**Abbreviations:**

Countries: AU=Austria, BE=Belgium, BU=Bulgaria, CR=Croatia, CY=Cyprus, CZ=Czech Republic, DE=Denmark, ES=Estonia, FIN=Finland, FR=France, GE=Germany, GR=Greece, HU=Hungary, ICE=Iceland , IRE=Ireland, ITA=Italy, LA=Latvia, LI=Lithuania, LU=Luxembourg, NE=Netherlands, NO=Norway, PO=Poland, PT= Portugal, RO=Romania, SL=Slovakia, SV=Slovenia, SP=Spain, SW=Sweden, SZ=Switzerland, UK=United Kingdom

Avg=Average

Causes: SCZ=Schizophrenia, DEP=Depressive disorders, BIP=Bipolar disorder, EATING=Eating disorders, ASD=Autism spectrum disorders, ADHD=Attention deficit hyperactivity disorders, CON=Conduct disorders, ID=Idiopathic development disorder, AUD=Alcohol use disorder, DUD=Drug use disorders, SH=Self-harm

Table 2. Overview over data-sources for mental and substance use disorders among males and females aged 10-24 in the European Union, Iceland, Norway, Switzerland and United Kingdom.

| Country | Cause | Males |  |  | Females |  |  | Both sexes |  |  |
| --- | --- | --- | --- | --- | --- | --- | --- | --- | --- | --- |
|  |  | 10-14 | 15-19 | 20-24 | 10-14 | 15-19 | 20-24 | 10-14 | 15-19 | 20-24 |
| Austria | Schizophrenia |  |  |  |  |  |  |  |  |  |
|  | Depressive disorders | ^1^ | ^1^ | ^2^ | ^1^ | ^1^ | ^2^ |  |  |  |
|  | Bipolar disorder |  |  |  |  |  |  | ^3^ | ^3^ | ^3^ |
|  | Anxiety disorders | ^1^ | ^1^ |  | ^1^ | ^1^ |  |  |  |  |
|  | Eating disorders | ^1^ | ^1^ |  | ^1^ | ^1^ |  |  |  |  |
|  | Autism spectrum disorders |  |  |  |  |  |  |  |  |  |
|  | Attention-deficit/ hypeactivity disorder | ^1^ | ^1^ |  | ^1^ | ^1^ |  |  |  |  |
|  | Conduct disorder | ^1^ | ^1^ |  | ^1^ | ^1^ |  |  |  |  |
|  | Idiopathic develpomental intellectual disability |  |  |  |  |  |  |  |  |  |
|  | Alcohol use disorder |  | ^4^ | ^4^ |  | ^4^ |  |  |  |  |
|  | Drug use disorder | ^5-8^ | ^5-20^ | ^11-18^ | ^5-8^ | ^5-20^ |  |  |  |  |
|  | Self-harm | ^21^ | ^21^ | ^21^ | ^21^ | ^21^ |  |  |  |  |
| Belgium | Schizophrenia |  |  |  |  |  |  |  |  |  |
|  | Depressive disorders |  | ^22-25^ | ^22-26^ |  | ^22-26^ | ^22-26^ |  |  |  |
|  | Bipolar disorder |  |  |  |  |  |  |  |  |  |
|  | Anxiety disorders |  |  | ^27^ |  |  | ^27^ |  |  |  |
|  | Eating disorders |  | ^28^ | ^28^ |  | ^28^ | ^28^ |  |  |  |
|  | Autism spectrum disorders |  |  |  |  |  |  |  |  |  |
|  | Attention-deficit/ hypeactivity disorder |  |  |  |  |  |  |  |  |  |
|  | Conduct disorder |  |  |  |  |  |  |  |  |  |
|  | Idiopathic develpomental intellectual disability |  |  |  |  |  |  |  |  |  |
|  | Alcohol use disorder |  | ^29,30^ | ^29^ |  | ^29,30^ |  |  |  |  |
|  | Drug use disorder | ^6,7,31^ | ^6,7,9-11,13,19,20,31,32^ | ^11,13,32^ | ^6,7,31^ | ^6,7,9-11,13,19,20,31,32^ | ^11,13,32^ |  |  |  |
|  | Self-harm | ^33,34^ | ^33,34^ | ^33,34^ | ^33,34^ | ^33,34^ | ^33,34^ |  |  |  |
| Bulgaria | Schizophrenia |  | ^35^ | ^35^ |  | ^35^ | ^35^ |  |  |  |
|  | Depressive disorders |  | ^24^ | ^24^ |  | ^24^ | ^24^ |  |  |  |
|  | Bipolar disorder |  | ^36^ | ^36^ |  |  |  |  |  |  |
|  | Anxiety disorders |  |  | ^27^ |  |  | ^27^ |  |  |  |
|  | Eating disorders |  |  |  |  |  |  |  |  |  |
|  | Autism spectrum disorders |  |  |  |  |  |  |  |  |  |
|  | Attention-deficit/ hypeactivity disorder |  |  |  |  |  |  |  |  |  |
|  | Conduct disorder |  |  |  |  |  |  |  |  |  |
|  | Idiopathic develpomental intellectual disability |  |  |  |  |  |  |  |  |  |
|  | Alcohol use disorder |  |  |  |  |  |  |  |  |  |
|  | Drug use disorder | ^6,37^ | ^6,9-13,19,20,32,37,38^ | ^11-13,32,38^ | ^6,37^ | ^6,9-13,19,20,32,37,38^ | ^11-13,32^ |  |  |  |
|  | Self-harm | ^39^ | ^39^ | ^39^ | ^39^ | ^39^ | ^39^ |  |  |  |
| Croatia | Schizophrenia |  |  |  |  |  |  |  |  |  |
|  | Depressive disorders |  | ^40^ | ^40^ |  | ^40^ | ^40^ |  |  |  |
|  | Bipolar disorder |  |  |  |  |  |  |  |  |  |
|  | Anxiety disorders |  |  |  |  |  |  |  |  |  |
|  | Eating disorders |  |  |  |  |  |  |  |  |  |
|  | Autism spectrum disorders |  |  |  |  |  |  |  |  |  |
|  | Attention-deficit/ hypeactivity disorder |  |  |  |  |  |  |  |  |  |
|  | Conduct disorder |  |  |  |  |  |  |  |  |  |
|  | Idiopathic develpomental intellectual disability |  |  |  |  |  |  |  |  |  |
|  | Alcohol use disorder | ^41,42^ |  |  | ^41,42^ |  |  |  |  |  |
|  | Drug use disorder | ^6,7,31,37^ | ^6,7,9-11,13,14,19,20,31,37,43^ | ^11,13,14^ | ^6,7,31,37^ | ^6,7,9-11,13,14,19,20,31,37,43^ | ^11,13,14^ |  |  |  |
|  | Self-harm | ^44,45^ | ^44,45^ | ^44,45^ | ^44,45^ | ^44,45^ | ^44,45^ |  |  |  |
| Cyprus | Schizophrenia |  |  |  |  |  |  |  |  |  |
|  | Depressive disorders | ^46^ | ^47^ | ^47^ | ^46^ | ^47^ | ^47^ |  |  |  |
|  | Bipolar disorder |  |  |  |  |  |  |  |  |  |
|  | Anxiety disorders |  |  |  |  |  |  |  |  |  |
|  | Eating disorders |  |  |  |  |  |  |  |  |  |
|  | Autism spectrum disorders |  |  |  |  |  |  |  |  |  |
|  | Attention-deficit/ hypeactivity disorder | ^48^ |  |  | ^48^ |  |  |  |  |  |
|  | Conduct disorder |  |  |  |  |  |  |  |  |  |
|  | Idiopathic develpomental intellectual disability |  |  |  |  |  |  |  |  |  |
|  | Alcohol use disorder |  |  |  |  |  |  |  |  |  |
|  | Drug use disorder |  | ^9-14,32,49^ | ^11-14,32,49^ |  | ^9-14,32,49^ | ^11-14,32,49^ |  |  |  |
|  | Self-harm | ^34,50^ | ^34,50^ | ^34,50^ | ^34,50^ | ^34,50^ | ^34,50^ |  |  |  |
| Czechia | Schizophrenia |  | ^35^ | ^35^ |  | ^35^ | ^35^ |  |  |  |
|  | Depressive disorders |  | ^51,52^ | ^51,52^ |  | ^51,52^ | ^51,52^ |  |  |  |
|  | Bipolar disorder |  |  |  |  |  |  |  |  |  |
|  | Anxiety disorders |  |  |  |  |  |  |  |  |  |
|  | Eating disorders |  |  |  |  |  |  |  |  |  |
|  | Autism spectrum disorders |  |  |  |  |  |  |  |  |  |
|  | Attention-deficit/ hypeactivity disorder |  |  |  |  |  |  |  |  |  |
|  | Conduct disorder |  |  |  |  |  |  |  |  |  |
|  | Idiopathic develpomental intellectual disability |  |  |  |  |  |  |  |  |  |
|  | Alcohol use disorder |  |  |  |  |  |  |  |  |  |
|  | Drug use disorder | ^6,7,31^ | ^6,7,9-14,19,20,31,32,53-60^ | ^11-14,32,53-56,58-60^ | ^6,7,31^ | ^6,7,9-14,19,20,31,32,53-60^ | ^11-14,32,53-56,58-60^ |  |  |  |
|  | Self-harm | ^34,61,62^ | ^34,61,62^ | ^34,61,62^ | ^34,61,62^ | ^34,61,62^ | ^34,61,62^ |  |  |  |
| Denmark | Schizophrenia | ^63,64^ | ^63-68^ | ^63,65-68^ | ^63,64^ | ^63-68^ | ^63,65-68^ |  |  |  |
|  | Depressive disorders | ^69^ | ^70,71^ | ^70,71^ | ^69^ | ^70,71^ | ^70,71^ |  |  |  |
|  | Bipolar disorder |  | ^72,73^ | ^72,73^ |  | ^72,73^ | ^72,73^ |  |  | ^74^ |
|  | Anxiety disorders | ^75^ | ^75^ | ^75^ | ^75^ | ^75^ | ^75^ |  |  |  |
|  | Eating disorders |  | ^76,77^ | ^76,77^ |  |  | ^76-78^ | ^76-78^ |  |  |
|  | Autism spectrum disorders | ^79-85^ | ^79-86^ | ^81,82,85,86^ | ^79-85^ | ^79-86^ | ^81,82,85,86^ |  |  |  |
|  | Attention-deficit/ hypeactivity disorder | ^87^ | ^87^ |  | ^87^ | ^87^ |  |  |  |  |
|  | Conduct disorder | ^88^ | ^88^ | ^88^ | ^88^ | ^88^ | ^88^ |  |  |  |
|  | Idiopathic develpomental intellectual disability |  |  |  |  |  |  |  |  |  |
|  | Alcohol use disorder |  |  |  |  |  |  |  |  |  |
|  | Drug use disorder | ^6,7,31^ | ^6,7,9-13,19,20,31,32,89-91^ | ^11-13,32,89-91^ | ^6,7,31^ | ^6,7,9-13,19,20,31,32,89-91^ | ^11-13,32,89-91^ |  |  |  |
|  | Self-harm | ^34,92^ | ^34,92^ | ^34,92^ | ^34,92^ | ^34,92^ | ^34,92^ |  |  |  |
| Estonia | Schizophrenia |  |  |  |  |  |  |  |  |  |
|  | Depressive disorders |  | ^93,94^ | ^93,94^ |  | ^93,94^ | ^93,94^ |  |  |  |
|  | Bipolar disorder |  |  |  |  |  |  |  |  |  |
|  | Anxiety disorders |  |  |  |  |  |  |  |  |  |
|  | Eating disorders |  |  |  |  |  |  |  |  |  |
|  | Autism spectrum disorders |  |  |  |  |  |  |  |  |  |
|  | Attention-deficit/ hypeactivity disorder |  |  |  |  |  |  |  |  |  |
|  | Conduct disorder |  |  |  |  |  |  |  |  |  |
|  | Idiopathic develpomental intellectual disability |  |  |  |  |  |  |  |  |  |
|  | Alcohol use disorder |  |  |  |  | ^4^ | ^4^ |  |  |  |
|  | Drug use disorder | ^5-7^ | ^5-7,9-13,19,20,32,43,95-97^ | ^11-13,32,95-97^ | ^5-7^ | ^5-7,9-13,19,20,32,43,95-97^ | ^11-13,32,95-97^ |  |  |  |
|  | Self-harm | ^98^ | ^98^ | ^98^ | ^98^ | ^98^ | ^98^ |  |  |  |
| Finland | Schizophrenia | ^99^ | ^99-101^ | ^99-101^ | ^99^ | ^99-101^ | ^99-101^ |  |  |  |
|  | Depressive disorders |  | ^102-104^ | ^102,104-106^ |  | ^102-104^ | ^102,104-106^ |  |  |  |
|  | Bipolar disorder |  |  | ^105^ |  |  | ^105^ | ^107^ | ^107^ | ^107^ |
|  | Anxiety disorders |  |  | ^105^ |  |  | ^105^ |  |  |  |
|  | Eating disorders |  | ^108,109^ | ^105^ | ^110^ | ^108-111^ | ^105,109,110^ |  |  |  |
|  | Autism spectrum disorders | ^112^ | ^112^ |  | ^112^ | ^112^ |  |  |  |  |
|  | Attention-deficit/ hypeactivity disorder |  | ^113^ |  |  | ^113^ |  |  |  |  |
|  | Conduct disorder |  |  |  |  |  |  |  |  |  |
|  | Idiopathic develpomental intellectual disability |  |  |  |  |  |  |  |  |  |
|  | Alcohol use disorder | ^114^ | ^114-116^ | ^105,114-116^ |  |  |  |  |  |  |
|  | Drug use disorder | ^6,7,31^ | ^6,7,9-11,13,14,19,20,31,32,43,53,117,118^ | ^11,13,14,32,53,117,118^ | ^6,7,31^ | ^6,7,9-11,13,14,19,20,31,32,43,53,117,118^ | ^11,13,14,32,53,117,118^ |  |  |  |
|  | Self-harm | ^34,119^ | ^34,119^ | ^34,119^ | ^34,119^ | ^34,119^ | ^34,119^ |  |  |  |
| France | Schizophrenia |  | ^120-122^ | ^120-122^ |  | ^120-122^ | ^120-122^ |  |  |  |
|  | Depressive disorders |  | ^23,26,123-126^ | ^23,26,123-127^ |  | ^23,26,123-126^ | ^23,26,123-127^ |  |  |  |
|  | Bipolar disorder |  | ^124^ | ^124^ |  | ^124^ | ^124^ |  |  |  |
|  | Anxiety disorders |  | ^128^ | ^128^ |  | ^128^ | ^128^ |  |  |  |
|  | Eating disorders | ^129^ | ^124,129,130^ | ^124,126,130,131^ | ^129^ | ^124,129,130^ | ^124,126,130,132^ |  |  |  |
|  | Autism spectrum disorders | ^133^ | ^28,133^ | ^28^ | ^133^ | ^28,133^ | ^28^ |  |  |  |
|  | Attention-deficit/ hypeactivity disorder | ^134^ |  |  | ^134^ |  |  |  |  |  |
|  | Conduct disorder | ^135,136^ | ^136^ |  | ^135,136^ | ^136^ |  |  |  |  |
|  | Idiopathic develpomental intellectual disability |  |  |  |  |  |  |  |  |  |
|  | Alcohol use disorder | ^137^ | ^137^ | ^137,138^ | ^137^ | ^137^ | ^137,138^ |  |  |  |
|  | Drug use disorder | ^6,7,31,139^ | ^6,7,9-11,13,19,20,31,32,139-144^ | ^11,13,32,142-145^ | ^6,7,31,139^ | ^6,7,9-11,13,19,20,31,32,139-144^ | ^11,13,32,142-145^ |  |  |  |
|  | Self-harm | ^146^ | ^146^ | ^146^ | ^146^ | ^146^ | ^146^ |  |  |  |
| Germany | Schizophrenia |  | ^35,147,148^ | ^35,147,148^ |  | ^35,147,148^ | ^35,147,148^ |  |  |  |
|  | Depressive disorders | ^26,149,150^ | ^23,24,26,149-155^ | ^23,24,26,125,151-155^ | ^26,149,150^ | ^23,24,26,149-155^ | ^23,24,26,125,151-155^ |  |  |  |
|  | Bipolar disorder |  | ^152,154,156,157^ | ^152,154,156,157^ |  | ^152,154,156,157^ | ^152,154,156,157^ |  |  |  |
|  | Anxiety disorders |  | ^151,152,154,158^ | ^151,152,154,155,158^ |  | ^151,152,154,158,159^ | ^151,152,154,155,158,159^ |  |  |  |
|  | Eating disorders |  | ^28,152,154^ | ^28,152,154^ |  | ^28,152,154,160-162^ | ^28,152,154,160^ |  |  |  |
|  | Autism spectrum disorders |  |  |  |  |  |  |  |  |  |
|  | Attention-deficit/ hypeactivity disorder | ^150,163-165^ | ^150,163,164^ |  | ^150,163,164^ | ^150,163-165^ |  |  |  |  |
|  | Conduct disorder | ^150^ | ^150^ |  | ^150^ | ^150^ |  |  |  |  |
|  | Idiopathic develpomental intellectual disability |  |  |  |  |  |  |  |  |  |
|  | Alcohol use disorder | ^166^ | ^152,154,166-173^ | ^152,154,166-173^ | ^166^ | ^152,154,166-173^ | ^152,154,166-173^ |  |  |  |
|  | Drug use disorder | ^6,7,31,174^ | ^6,7,9-11,13,17,19,20,31,169,174-181^ | ^11,13,17,169,174-181^ | ^6,7,31,174^ | ^6,7,9-11,13,17,19,20,31,169,174-181^ | ^11,13,17,169,174-181^ |  |  |  |
|  | Self-harm | ^182^ | ^182^ | ^182^ | ^182^ | ^182^ | ^182^ |  |  |  |
| Greece | Schizophrenia |  |  |  |  |  |  |  |  |  |
|  | Depressive disorders |  | ^183-187^ | ^183-187^ |  | ^183-187^ | ^183-187^ |  |  |  |
|  | Bipolar disorder |  |  |  |  |  |  |  |  |  |
|  | Anxiety disorders |  | ^185^ | ^185^ |  | ^185^ | ^185^ |  |  |  |
|  | Eating disorders | ^188^ | ^188^ | ^188^ | ^188^ | ^188^ | ^188^ |  |  |  |
|  | Autism spectrum disorders |  |  |  |  |  |  |  |  |  |
|  | Attention-deficit/ hypeactivity disorder | ^189^ |  |  | ^189^ |  |  |  |  |  |
|  | Conduct disorder |  |  |  |  |  |  |  |  |  |
|  | Idiopathic develpomental intellectual disability |  |  |  |  |  |  |  |  |  |
|  | Alcohol use disorder |  |  |  |  |  |  |  |  |  |
|  | Drug use disorder | ^6,7,31^ | ^6,7,9-11,13,14,19,20,31,43,140,185,190,191^ | ^11,13,14,185,190,191^ | ^6,7,31^ | ^6,7,9-11,13,14,19,20,31,43,140,185,190,191^ | ^11,13,14,185,190,191^ |  |  |  |
|  | Self-harm | ^192^ | ^192^ | ^192^ | ^192^ | ^192^ | ^192^ |  |  |  |
| Hungary | Schizophrenia |  |  |  |  |  |  |  |  |  |
|  | Depressive disorders | ^193^ | ^193^ |  | ^193^ | ^193^ |  |  |  |  |
|  | Bipolar disorder |  |  |  |  |  |  |  |  |  |
|  | Anxiety disorders |  |  |  |  |  |  |  |  |  |
|  | Eating disorders | ^194^ | ^194^ |  |  | ^194,195^ | ^194,195^ |  |  |  |
|  | Autism spectrum disorders |  |  |  |  |  |  |  |  |  |
|  | Attention-deficit/ hypeactivity disorder |  | ^196^ | ^196^ |  | ^196^ | ^196^ |  |  |  |
|  | Conduct disorder |  |  |  |  |  |  |  |  |  |
|  | Idiopathic develpomental intellectual disability |  |  |  |  |  |  |  |  |  |
|  | Alcohol use disorder |  |  |  |  |  |  |  |  |  |
|  | Drug use disorder | ^6,7,31^ | ^6,7,9-11,13,19,20,31,32,43,53,197^ | ^11,13,32,53^ | ^6,7,31^ | ^6,7,9-11,13,19,20,31,32,43,53,197^ | ^11,13,32,53^ |  |  |  |
|  | Self-harm | ^198^ | ^198^ | ^198^ | ^198^ | ^198^ | ^198^ |  |  |  |
| Iceland | Schizophrenia |  |  |  |  |  |  |  |  |  |
|  | Depressive disorders |  |  |  |  |  |  |  |  |  |
|  | Bipolar disorder | ^199^ | ^199^ | ^199^ | ^199^ | ^199^ | ^199^ |  |  |  |
|  | Anxiety disorders |  |  |  |  |  |  |  |  |  |
|  | Eating disorders |  |  |  |  |  |  |  |  |  |
|  | Autism spectrum disorders | ^200-202^ | ^201,202^ | ^201^ | ^200-202^ | ^201,202^ | ^201^ |  |  |  |
|  | Attention-deficit/ hypeactivity disorder |  |  |  |  |  |  |  |  |  |
|  | Conduct disorder |  |  |  |  |  |  |  |  |  |
|  | Idiopathic develpomental intellectual disability |  |  |  |  |  |  |  |  |  |
|  | Alcohol use disorder |  | ^203^ | ^203^ |  |  |  |  |  |  |
|  | Drug use disorder | ^6,204^ | ^6,9,10,19,20,43,204^ |  | ^6,204^ | ^6,9,10,19,20,43,204^ |  |  |  |  |
|  | Self-harm | ^34,205,206^ | ^34,205,206^ | ^34,205,206^ | ^34,205,206^ | ^34,205,206^ | ^34,205,206^ |  |  |  |
| Ireland | Schizophrenia |  | ^35,207-209^ | ^35,207-210^ |  | ^35,207-209^ | ^35,207-210^ |  |  |  |
|  | Depressive disorders | ^211^ | ^24,102,193,212^ | ^24,102,193,212^ | ^211^ | ^24,102,193,212^ | ^24,102,193,212^ |  |  |  |
|  | Bipolar disorder |  | ^67,207,213,214^ | ^67,207,213,214^ |  | ^67,207,213,214^ | ^67,207,213,214^ |  |  |  |
|  | Anxiety disorders | ^211,215^ | ^207,215^ | ^207^ | ^211,215^ | ^207,215^ | ^207^ |  |  |  |
|  | Eating disorders |  | ^207^ | ^207^ |  | ^207^ | ^207^ |  |  |  |
|  | Autism spectrum disorders | ^216^ |  |  | ^216^ |  |  |  |  |  |
|  | Attention-deficit/ hypeactivity disorder |  |  |  |  |  |  |  |  |  |
|  | Conduct disorder |  |  |  |  |  |  |  |  |  |
|  | Idiopathic develpomental intellectual disability |  |  |  |  |  |  |  |  |  |
|  | Alcohol use disorder |  | ^217^ | ^217^ |  |  |  |  |  |  |
|  | Drug use disorder | ^6,7,31,218^ | ^6,7,9-11,13,17,19,20,31,32,197,207,218-222^ | ^11,13,17,32,207,219-222^ | ^6,7,31^ | ^6,7,9-11,13,31,32^ | ^11,13,32^ |  |  |  |
|  | Self-harm | ^223^ | ^223^ | ^223^ | ^223^ | ^223^ | ^223^ |  |  |  |
| Italy | Schizophrenia |  |  |  |  |  |  |  |  |  |
|  | Depressive disorders |  | ^23,224^ | ^23,224^ |  | ^23,224^ | ^23,224^ |  |  |  |
|  | Bipolar disorder |  |  |  |  |  |  |  | ^225^ | ^225^ |
|  | Anxiety disorders |  |  | ^224^ |  |  | ^224^ |  |  |  |
|  | Eating disorders |  | ^28^ | ^28^ |  | ^28^ | ^28^ |  |  |  |
|  | Autism spectrum disorders |  |  |  |  |  |  |  |  |  |
|  | Attention-deficit/ hyperactivity disorder | ^226^ | ^226^ |  | ^226^ | ^226^ |  |  |  |  |
|  | Conduct disorder |  |  |  |  |  |  |  |  |  |
|  | Idiopathic develpomental intellectual disability |  |  |  |  |  |  |  |  |  |
|  | Alcohol use disorder |  |  |  |  |  |  |  |  |  |
|  | Drug use disorder | ^6,31,204^ | ^6,9-11,13,14,17,19,20,31,32,43,53,140,197,204,227-231^ | ^11,13,14,17,32,53,227-231^ | ^6,31,204^ | ^6,9-11,13,14,17,19,20,31,32,43,53,140,197,204,227-231^ | ^11,13,14,17,32,53,227-231^ |  |  |  |
|  | Self-harm | ^232^ | ^232^ | ^232^ | ^232^ | ^232^ | ^232^ |  |  |  |
| Latvia | Schizophrenia |  |  |  |  |  |  |  |  |  |
|  | Depressive disorders |  | ^233-235^ | ^233-235^ |  | ^233-235^ | ^233-235^ |  |  |  |
|  | Bipolar disorder |  |  |  |  |  |  |  |  |  |
|  | Anxiety disorders |  |  |  |  |  |  |  |  |  |
|  | Eating disorders |  |  |  |  |  |  |  |  |  |
|  | Autism spectrum disorders |  |  |  |  |  |  |  |  |  |
|  | Attention-deficit/ hypeactivity disorder |  |  |  |  |  |  |  |  |  |
|  | Conduct disorder |  |  |  |  |  |  |  |  |  |
|  | Idiopathic develpomental intellectual disability |  |  |  |  |  |  |  |  |  |
|  | Alcohol use disorder |  |  |  |  |  |  |  |  |  |
|  | Drug use disorder | ^5,6,204^ | ^5,6,9-11,13,14,19,20,32,43,140,197,204^ | ^11,13,14,32^ | ^5,6,204^ | ^5,6,9-11,13,14,19,20,32,43,140,197,204^ | ^11,13,14,32^ |  |  |  |
|  | Self-harm | ^34,236^ | ^34,236^ | ^34,236^ | ^34,236^ | ^34,236^ | ^34,236^ |  |  |  |
| Lithuania | Schizophrenia |  |  |  |  |  |  |  |  |  |
|  | Depressive disorders |  |  |  |  |  |  |  |  |  |
|  | Bipolar disorder |  |  |  |  |  |  |  |  |  |
|  | Anxiety disorders | ^237^ | ^237^ |  | ^237^ | ^237^ |  |  |  |  |
|  | Eating disorders |  |  |  |  |  |  |  |  |  |
|  | Autism spectrum disorders | ^237^ | ^237^ |  | ^237^ | ^237^ |  |  |  |  |
|  | Attention-deficit/ hypeactivity disorder |  |  |  |  |  |  |  |  |  |
|  | Conduct disorder |  |  |  |  |  |  |  |  |  |
|  | Idiopathic develpomental intellectual disability |  |  |  |  |  |  |  |  |  |
|  | Alcohol use disorder |  |  |  |  |  |  |  |  |  |
|  | Drug use disorder | ^6,7,31^ | ^6,7,9-11,13,14,19,20,31,32,43,197,238^ | ^11,13,14,32,238^ | ^6,7,31^ | ^6,7,9-11,13,14,19,20,31,32,43,197,238^ | ^11,13,14,32,238^ |  |  |  |
|  | Self-harm | ^34,239^ | ^34,239^ | ^34,239^ | ^34,239^ | ^34,239^ | ^34,239^ |  |  |  |
| Luxembourg | Schizophrenia |  |  |  |  |  |  |  |  |  |
|  | Depressive disorders |  | ^240^ | ^240^ |  | ^240^ | ^240^ |  |  |  |
|  | Bipolar disorder |  |  |  |  |  |  |  |  |  |
|  | Anxiety disorders |  |  |  |  |  |  |  |  |  |
|  | Eating disorders |  |  |  |  |  |  |  |  |  |
|  | Autism spectrum disorders |  |  |  |  |  |  |  |  |  |
|  | Attention-deficit/ hypeactivity disorder |  |  |  |  |  |  |  |  |  |
|  | Conduct disorder |  |  |  |  |  |  |  |  |  |
|  | Idiopathic develpomental intellectual disability |  |  |  |  |  |  |  |  |  |
|  | Alcohol use disorder |  |  |  |  |  |  |  |  |  |
|  | Drug use disorder | ^6,7^ | ^6,7,11,14,17,19,20,228^ | ^11,14,17,228^ | ^6,7^ | ^6,7,11,14,17,19,20,228^ | ^11,14,17,228^ |  |  |  |
|  | Self-harm | ^34,241^ | ^34,241^ | ^34,241^ | ^34,241^ | ^34,241^ | ^34,241^ |  |  |  |
| Netherlands | Schizophrenia |  | ^35,242,243^ | ^35,242-244^ |  | ^35,242,243^ | ^35,242-244^ |  |  |  |
|  | Depressive disorders | ^245^ | ^23,24,26,242,245-250^ | ^23,24,26,242,246-250^ | ^245^ | ^23,24,26,242,245-250^ | ^23,24,26,242,246-250^ |  |  |  |
|  | Bipolar disorder |  | ^242,246,247^ | ^242,246,247^ |  | ^242,246,247^ | ^242,246,247^ |  |  |  |
|  | Anxiety disorders | ^245^ | ^242,245-248,251-253^ | ^242,246-248,250-253^ | ^245^ | ^242,245-248,251-253^ | ^242,246-248,250-253^ |  |  |  |
|  | Eating disorders | ^245,254^ | ^28,242,245,254,255^ | ^28,242,254^ | ^245,254^ | ^28,242,245,254,255^ | ^28,242,254^ |  |  |  |
|  | Autism spectrum disorders | ^256^ | ^256^ |  | ^256^ | ^256^ |  |  |  |  |
|  | Attention-deficit/ hypeactivity disorder | ^245^ | ^245^ |  | ^245^ | ^245^ |  |  |  |  |
|  | Conduct disorder | ^245^ | ^245^ |  | ^245^ | ^245^ |  |  |  |  |
|  | Idiopathic develpomental intellectual disability |  |  |  |  |  |  |  |  |  |
|  | Alcohol use disorder |  | ^246,257-260^ | ^246,257-260^ |  | ^246,257-260^ | ^246,257-260^ |  |  |  |
|  | Drug use disorder | ^261-268^ | ^9-11,13,14,17,19,20,32,197,261-276^ | ^11,13,14,17,32,264-276^ | ^261-268^ | ^9-11,13,14,17,19,20,32,197,261-276^ | ^11,13,14,17,32,264-276^ |  |  |  |
|  | Self-harm | ^277,278^ | ^277,278^ | ^277,278^ | ^277,278^ | ^277,278^ | ^277,278^ |  |  |  |
| Norway | Schizophrenia |  | ^279^ | ^279,280^ |  | ^279^ | ^279,280^ |  |  |  |
|  | Depressive disorders | ^281^ | ^102,282-289^ | ^102,282,284,287-290^ | ^281^ | ^102,282-289^ | ^102,282,284,287-290^ |  |  |  |
|  | Bipolar disorder |  | ^289^ | ^289^ |  | ^289^ | ^289^ |  |  |  |
|  | Anxiety disorders |  |  | ^291^ |  |  | ^291^ |  |  |  |
|  | Eating disorders |  |  |  |  | ^292^ | ^292^ |  |  |  |
|  | Autism spectrum disorders | ^293,294^ |  |  | ^293,294^ |  |  |  |  |  |
|  | Attention-deficit/ hypeactivity disorder |  |  |  |  |  |  |  |  |  |
|  | Conduct disorder |  |  |  |  |  |  |  |  |  |
|  | Idiopathic develpomental intellectual disability |  |  |  |  |  |  |  |  |  |
|  | Alcohol use disorder |  | ^284,288^ | ^284,288^ |  | ^284,288^ | ^284,288^ |  |  |  |
|  | Drug use disorder | ^204,295^ | ^9-11,13,14,19,32,204,295-299^ | ^11,13,14,32,295-300^ | ^204,295^ | ^9-11,13,14,19,32,204,295-299^ | ^11,13,14,32,295-300^ |  |  |  |
|  | Self-harm | ^301^ | ^301^ | ^301^ | ^301^ | ^301^ | ^301^ |  |  |  |
| Poland | Schizophrenia |  |  |  |  |  |  |  |  |  |
|  | Depressive disorders |  | ^302^ | ^302^ |  | ^302^ | ^302^ |  |  |  |
|  | Bipolar disorder |  |  |  |  |  |  | ^303^ | ^303^ | ^303^ |
|  | Anxiety disorders |  |  |  |  |  |  |  |  |  |
|  | Eating disorders |  |  |  |  |  |  |  |  |  |
|  | Autism spectrum disorders |  |  |  |  |  |  |  |  |  |
|  | Attention-deficit/ hypeactivity disorder |  |  |  |  |  |  |  |  |  |
|  | Conduct disorder |  |  |  |  |  |  |  |  |  |
|  | Idiopathic develpomental intellectual disability |  |  |  |  |  |  |  |  |  |
|  | Alcohol use disorder |  | ^304^ | ^304^ |  | ^304^ | ^304^ |  |  |  |
|  | Drug use disorder | ^6,7,31^ | ^6,7,9-11,13,19,20,31,32,43,140,197,305^ | ^11,13,32^ | ^6,7,31^ | ^6,7,9-11,13,19,20,31,32,43,140,197,305^ | ^11,13,32^ |  |  |  |
|  | Self-harm | ^34,306^ | ^34,306^ | ^34,306^ | ^34,306^ | ^34,306^ | ^34,306^ |  |  |  |
| Portugal | Schizophrenia |  |  |  |  |  |  |  |  |  |
|  | Depressive disorders | ^307^ | ^24,308^ | ^24,308^ | ^307^ | ^24,308^ | ^24,308^ |  |  |  |
|  | Bipolar disorder |  | ^309^ | ^309^ |  | ^309^ | ^309^ |  |  |  |
|  | Anxiety disorders |  | ^309^ | ^309^ |  | ^309^ | ^309^ |  |  |  |
|  | Eating disorders | ^310^ | ^28,310^ | ^28,310^ | ^310,311^ | ^28,310,311^ | ^28,310,311^ |  |  |  |
|  | Autism spectrum disorders |  |  |  |  |  |  |  |  |  |
|  | Attention-deficit/ hypeactivity disorder |  |  |  |  |  |  |  |  |  |
|  | Conduct disorder |  |  |  |  |  |  |  |  |  |
|  | Idiopathic develpomental intellectual disability |  |  |  |  |  |  |  |  |  |
|  | Alcohol use disorder |  |  |  |  | ^4^ | ^4^ |  |  |  |
|  | Drug use disorder | ^6,31,204,312^ | ^6,9-11,13,19,20,31,32,197,204^ | ^11,13,32^ | ^6,31,204,312^ | ^6,9-11,13,19,20,31,32,197,204^ | ^11,13,32^ |  |  |  |
|  | Self-harm | ^313,314^ | ^313,314^ | ^313,314^ | ^313,314^ | ^313,314^ | ^313,314^ |  |  |  |
| Romania | Schizophrenia |  |  |  |  |  |  |  |  |  |
|  | Depressive disorders |  | ^24^ | ^24^ |  | ^24^ | ^24^ |  |  |  |
|  | Bipolar disorder |  | ^36^ | ^36^ |  | ^36^ | ^36^ |  |  |  |
|  | Anxiety disorders |  |  | ^27^ |  |  |  |  |  |  |
|  | Eating disorders |  | ^28^ | ^28^ |  | ^28^ | ^28^ |  |  |  |
|  | Autism spectrum disorders |  |  |  |  |  |  |  |  |  |
|  | Attention-deficit/ hypeactivity disorder |  |  |  |  |  |  |  |  |  |
|  | Conduct disorder |  |  |  |  |  |  |  |  |  |
|  | Idiopathic develpomental intellectual disability |  |  |  |  |  |  |  |  |  |
|  | Alcohol use disorder |  |  | ^38^ |  |  |  |  |  |  |
|  | Drug use disorder | ^6,7^ | ^6,7,9-11,13,19,20,32^ | ^11,13,32^ | ^6,7^ | ^6,7,9-11,13,19,20,32^ | ^11,13,32^ |  |  |  |
|  | Self-harm | ^34,315^ | ^34,315^ | ^34,315^ | ^34,315^ | ^34,315^ | ^34,315^ |  |  |  |
| Slovakia | Schizophrenia |  |  |  |  |  |  |  |  |  |
|  | Depressive disorders |  | ^316^ | ^316^ |  | ^316^ | ^316^ |  |  |  |
|  | Bipolar disorder |  |  |  |  |  |  |  |  |  |
|  | Anxiety disorders |  |  |  |  |  |  |  |  |  |
|  | Eating disorders |  |  |  |  |  |  |  |  |  |
|  | Autism spectrum disorders |  |  |  |  |  |  |  |  |  |
|  | Attention-deficit/ hypeactivity disorder |  |  |  |  |  |  |  |  |  |
|  | Conduct disorder |  |  |  |  |  |  |  |  |  |
|  | Idiopathic develpomental intellectual disability |  |  |  |  |  |  |  |  |  |
|  | Alcohol use disorder |  |  |  |  |  |  |  |  |  |
|  | Drug use disorder | ^6,7,317^ | ^6,7,9-11,13,14,19,20,32,53,140,317-320^ | ^11,13,14,32,53,317,318,320^ | ^6,7,317^ | ^6,7,9-11,13,14,19,20,32,53,140,317-320^ | ^11,13,14,32,53,317,318,320^ |  |  |  |
|  | Self-harm | ^34,321^ | ^34,321^ | ^34,321^ | ^34,321^ | ^34,321^ | ^34,321^ |  |  |  |
| Slovenia | Schizophrenia |  |  |  |  |  |  |  |  |  |
|  | Depressive disorders |  | ^322^ | ^322^ |  | ^322^ | ^322^ |  |  |  |
|  | Bipolar disorder |  |  |  |  |  |  |  |  |  |
|  | Anxiety disorders |  |  |  |  |  |  |  |  |  |
|  | Eating disorders |  |  |  |  |  |  |  |  |  |
|  | Autism spectrum disorders |  |  |  |  |  |  |  |  |  |
|  | Attention-deficit/ hypeactivity disorder |  |  |  |  |  |  |  |  |  |
|  | Conduct disorder |  |  |  |  |  |  |  |  |  |
|  | Idiopathic develpomental intellectual disability |  |  |  |  |  |  |  |  |  |
|  | Alcohol use disorder |  |  |  |  |  |  |  |  |  |
|  | Drug use disorder | ^6,7,31^ | ^6,7,9-11,13,14,19,20,31,32,43,197,323^ | ^11,13,14,32,323^ | ^6,7,31^ | ^6,7,9-11,13,14,19,20,31,32,43,197,323^ | ^11,13,14,32,323^ |  |  |  |
|  | Self-harm | ^34,324^ | ^34,324^ | ^34,324^ | ^34,324^ | ^34,324^ | ^34,324^ |  |  |  |
| Spain | Schizophrenia | ^325,326^ | ^325,326^ | ^325,326^ | ^325,326^ | ^325,326^ | ^325,326^ |  |  |  |
|  | Depressive disorders | ^327-329^ | ^23,26,102,330-333^ | ^23,26,102,331-333^ | ^327-329^ | ^23,26,102,330-333^ | ^23,26,102,331-333^ |  |  |  |
|  | Bipolar disorder |  | ^331^ | ^331^ |  | ^331^ | ^331^ | ^334^ | ^334,335^ | ^334,335^ |
|  | Anxiety disorders | ^336^ | ^330,331,333,337^ | ^331,333,337^ | ^336^ | ^330,331,333,337^ | ^331,333,337^ |  |  |  |
|  | Eating disorders | ^338-341^ | ^28,330,338-340^ | ^28,339,340^ | ^338-343^ | ^28,330,338-343^ | ^28,339,340,342,343^ |  |  |  |
|  | Autism spectrum disorders |  |  |  |  |  |  |  |  |  |
|  | Attention-deficit/ hyperactivity disorder | ^344-350^ | ^345,348-350^ |  | ^344-350^ | ^345,348-350^ |  |  |  |  |
|  | Conduct disorder |  |  |  |  |  |  |  |  |  |
|  | Idiopathic develpomental intellectual disability |  |  |  |  |  |  |  |  |  |
|  | Alcohol use disorder |  | ^351,352^ | ^351,352^ |  | ^351,352^ | ^351,352^ |  |  |  |
|  | Drug use disorder | ^6,7,31,353-360^ | ^6,7,11,13,14,19,20,31,32,197,351,353-378^ | ^11,13,14,32,351,360-377,379^ | ^6,7,31,353-360^ | ^6,7,11,13,14,19,20,31,32,197,351,353-378^ | ^11,13,14,32,351,360-377,379^ |  |  |  |
|  | Self-harm | ^380,381^ | ^380,381^ | ^380,381^ | ^380,381^ | ^380,381^ | ^380,381^ |  |  |  |
| Sweden | Schizophrenia |  | ^382-387^ | ^382-387^ |  | ^382,385-387^ | ^382,385-387^ |  |  |  |
|  | Depressive disorders |  | ^384,388-390^ | ^384,389-391^ |  | ^388-390^ | ^389-391^ |  |  |  |
|  | Bipolar disorder |  |  | ^392,393^ |  |  | ^392,393^ |  |  |  |
|  | Anxiety disorders |  |  |  |  |  |  |  |  |  |
|  | Eating disorders | ^394^ | ^394,395^ | ^394,395^ | ^394,396,397^ | ^394-399^ | ^394-399^ |  |  |  |
|  | Autism spectrum disorders | ^385,400-404^ | ^401-404^ | ^401-404^ | ^385,400-404^ | ^401-404^ | ^401-404^ |  |  |  |
|  | Attention-deficit/ hyperactivity disorder | ^405,406^ | ^406^ | ^406^ | ^405,406^ | ^406^ | ^406^ |  |  |  |
|  | Conduct disorder |  |  |  |  |  |  |  |  |  |
|  | Idiopathic develpomental intellectual disability |  |  |  |  |  |  |  |  |  |
|  | Alcohol use disorder | ^407^ | ^391,407-418^ | ^391,407-419^ | ^407^ | ^391,407,409,411-418^ | ^391,407,409,411-421^ |  | ^422^ | ^422^ |
|  | Drug use disorder |  | ^9-11,13,19,20,43,140,197,423-429^ | ^9,11,13,423,424,426-429^ |  | ^9-11,13,19,20,43,140,197,423-429^ | ^9,11,13,423,424,426-429^ |  |  |  |
|  | Self-harm | ^430,431^ | ^430,431^ | ^430,431^ | ^430,431^ | ^430,431^ | ^430,431^ |  |  |  |
| Switzerland | Schizophrenia |  |  |  |  |  |  |  |  |  |
|  | Depressive disorders |  | ^432,433^ | ^432,433^ |  | ^432,433^ | ^432,433^ |  |  |  |
|  | Bipolar disorder |  |  |  |  |  |  |  |  |  |
|  | Anxiety disorders |  | ^432^ | ^432^ |  | ^432^ | ^432^ |  |  |  |
|  | Eating disorders | ^434^ | ^432,434^ | ^432^ | ^434^ | ^432,434^ | ^432^ |  |  |  |
|  | Autism spectrum disorders |  |  |  |  |  |  |  |  |  |
|  | Attention-deficit/ hyperactivity disorder | ^435^ | ^435^ |  | ^435^ | ^435^ |  |  |  |  |
|  | Conduct disorder |  |  |  |  |  |  |  |  |  |
|  | Idiopathic develpomental intellectual disability |  |  |  |  |  |  |  |  |  |
|  | Alcohol use disorder |  | ^4,436,437^ | ^4,436,437^ |  | ^4,436^ | ^4,436^ |  |  |  |
|  | Drug use disorder | ^6,7,31^ | ^6,7,9,10,19,20,31,197^ | ^438^ | ^6,7,31^ | ^6,7,9,10,19,20,31,197^ |  |  |  |  |
|  | Self-harm | ^34,439^ | ^34,439^ | ^34,439^ | ^34,439^ | ^34,439^ | ^34,439^ |  |  |  |
| United Kingdom | Schizophrenia | ^440^ | ^35,214,440-449^ | ^35,214,440-449^ | ^440^ | ^35,214,440-450^ | ^35,214,440-450^ |  |  |  |
|  | Depressive disorders | ^451-453^ | ^26,102,213,451-461^ | ^26,102,213,454-459,461^ | ^451-453^ | ^26,102,213,451-461^ | ^26,102,213,454-459,461^ |  |  |  |
|  | Bipolar disorder |  | ^213,462,463^ | ^213,462,463^ |  | ^213,462,463^ | ^213,462,463^ | ^464^ | ^464,465^ | ^464,465^ |
|  | Anxiety disorders | ^453,466^ | ^213,453,455,460,466^ | ^213,455^ | ^453,466^ | ^213,453,455,460,466^ | ^213,455^ |  |  |  |
|  | Eating disorders | ^467,468^ | ^28,460,467-470^ | ^28,467-470^ | ^467,468^ | ^28,460,467-472^ | ^28,467-472^ |  |  |  |
|  | Autism spectrum disorders | ^473-479^ | ^473-475,477,480,481^ | ^480,481^ | ^473-479^ | ^473-475,477,480,481^ | ^480,481^ |  |  |  |
|  | Attention-deficit/ hyperactivity disorder | ^453,466,482-485^ | ^453,466,482,484^ |  | ^453,466,482-485^ | ^453,466,482,484^ |  |  |  |  |
|  | Conduct disorder | ^453,466^ | ^453,466^ |  | ^453,466^ | ^453,466^ |  |  |  |  |
|  | Idiopathic develpomental intellectual disability |  |  |  |  |  |  |  |  |  |
|  | Alcohol use disorder |  | ^455,458,481,486-489^ | ^455,458,481,486-489^ | ^455,458,481,487-489^ | ^455,458,481,487-489^ |  |  |  |  |
|  | Drug use disorder | ^6,31,204,295,453,490-499^ | ^6,10,11,13,14,19,20,31,32,197,204,219,220,269,295,453,456,457,481,490-523^ | ^11,13,14,32,219,220,269,456,457,481,500-522^ | ^6,31,204,295,453,490-499,524^ | ^6,10,11,13,14,19,20,31,32,197,204,219,220,269,295,453,456,457,481,490-507,509-524^ | ^11,13,14,32,219,220,269,456,457,481,500-507,509-522^ |  |  |  |
|  | Self-harm | ^525-528^ | ^525-528^ | ^525-528^ | ^525-528^ | ^525-528^ | ^525-528^ |  |  |  |

# References

1. Wagner G, Zeiler M, Waldherr K, et al. Mental health problems in Austrian adolescents: a nationwide, two-stage epidemiological study applying DSM-5 criteria. *Eur Child Adolesc Psychiatry* 2017; **26**(12): 1483-99.

2. World Health Organization (WHO). Austria World Health Survey 2003. Geneva, Switzerland: World Health Organization (WHO), 2005.

3. Fellinger M, Waldhor T, Bluml V, Williams N, Vyssoki B. Influence of gender on inpatient treatment for bipolar disorder: An analysis of 60,607 hospitalisations. *J Affect Disord* 2018; **225**: 104-7.

4. World Health Organization Regional Office for Europe (WHO/Europe). Alcohol in the European Region: Consumption, Harm and Policies. Copenhagen, Denmark: World Health Organization Regional Office for Europe (WHO/Europe), 2001.

5. World Health Organization Regional Office for Europe (EURO-WHO). Health Behaviour in School-aged Children: WHO Collaborative Cross-National survey/study (HBSC) 2001, 2001.

6. World Health Organization Regional Office for Europe (EURO-WHO). Health Behaviour in School-aged Children: WHO Collaborative Cross-National survey/study (HBSC), 2006.

7. World Health Organization Regional Office for Europe (EURO-WHO). Health Behaviour in School-aged Children: WHO Collaborative Cross-National survey/study (HBSC), 2010.

8. United Nations Office on Drugs and Crime (UNODC). World Drug Report 2008. Vienna, Austria: United Nations Office on Drugs and Crime (UNODC), 2008.

9. European Monitoring Centre for Drugs and Drug Addiction (EMCDDA). ESPAD Report 2015: Results from the European School Survey Project on Alcohol and Other Drugs. Lisbon, Portugal: European School Survey Project on Alcohol and Other Drugs (ESPAD), 2016.

10. European School Survey Project on Alcohol and Other Drugs (ESPAD), Pompidou Group CoE, Swedish Council for Information on Alcohol and Other Drugs (CAN). ESPAD Report 2003: Alcohol and Other Drug Use Among Students in 35 European Countries. Stockholm, Sweden: Swedish Council for Information on Alcohol and Other Drugs (CAN), 2004.

11. European Monitoring Centre for Drugs and Drug Addiction (EMCDDA). European Monitoring Centre for Drugs and Drug Addiction Statistical Bulletin 2008. Lisbon, Portugal: European Monitoring Centre for Drugs and Drug Addiction (EMCDDA), 2008.

12. European Monitoring Centre for Drugs and Drug Addiction (EMCDDA). European Monitoring Centre for Drugs and Drug Addiction Statistical Bulletin 2012 Lisbon, Portugal: European Monitoring Centre for Drugs and Drug Addiction (EMCDDA), 2012.

13. European Monitoring Centre for Drugs and Drug Addiction (EMCDDA). European Monitoring Centre for Drugs and Drug Addiction Statistical Bulletin 2013. Lisbon, Portugal: European Monitoring Centre for Drugs and Drug Addiction (EMCDDA), 2013.

14. European Monitoring Centre for Drugs and Drug Addiction (EMCDDA). European Monitoring Centre for Drugs and Drug Addiction Statistical Bulletin 2015. Lisbon, Portugal: European Monitoring Centre for Drugs and Drug Addiction (EMCDDA), 2015.

15. Bauer SM, Loipl R, Jagsch R, et al. Mortality in opioid-maintained patients after release from an addiction clinic. *Eur Addict Res* 2008; **14**(2): 82-91.

16. European Monitoring Centre for Drugs and Drug Addiction (EMCDDA). Austria National Report to the EMCDDA 2006, 2006.

17. Kraus L, Augustin R, Frischer M, Kümmler P, Uhl A, Wiessing L. Estimating prevalence of problem drug use at national level in countries of the European Union and Norway. *Addiction* 2003; **98**(4): 471-85.

18. Risser D, Honigschnabl S, Stichenwirth M, et al. Mortality of opiate users in Vienna, Austria. *Drug Alcohol Depend* 2001; **64**(3): 251-6.

19. Shi Y, Lenzi M, An R. Cannabis Liberalization and Adolescent Cannabis Use: A Cross-National Study in 38 Countries. *Plos One* 2015; **10**(11).

20. World Health Organization Regional Office for Europe (WHO/Europe). Inequalities in Young People's Health: HBSC International Report from the 2005-2006 Survey. Copenhagen, Denmark: World Health Organization Regional Office for Europe (WHO/Europe), 2008.

21. Registration AV. Deaths ICD10 as it appears in World Health Organization (WHO). WHO Mortality Database . . Geneva, Switzerland: World Health Organization (WHO).

22. Bracke P. Sex differences in the course of depression: evidence from a longitudinal study of a representative sample of the Belgian population. *Soc Psychiatry Psychiatr Epidemiol* 1998; **33**(9): 420-9.

23. Kessler RC, Birnbaum HG, Shahly V, et al. Age differences in the prevalence and co-morbidity of DSM-IV major depressive episodes: results from the WHO World Mental Health Survey Initiative. *Depress Anxiety* 2010; **27**(4): 351-64.

24. McDowell RD, Ryan A, Bunting BP, et al. Mood and anxiety disorders across the adult lifespan: a European perspective. *Psychol Med* 2014; **44**(4): 707-22.

25. World Health Organization (WHO). Belgium World Health Survey 2002. Geneva, Switzerland: World Health Organization (WHO), 2005.

26. Lepine JP, Gastpar M, Mendlewicz J, Tylee A. Depression in the community: the first pan-European study DEPRES (Depression Research in European Society). *Int Clin Psychopharmacol* 1997; **12**(1): 19-29.

27. World Health Organization (WHO). WHO World Mental Health Surveys: Global Perspectives on the Epidemiology of Mental Disorders. Cambridge, United Kingdom: Cambridge University Press; 2011.

28. Kessler RC, Berglund PA, Chiu WT, et al. The prevalence and correlates of binge eating disorder in the World Health Organization World Mental Health Surveys. *Biol Psychiatry* 2013; **73**(9): 904-14.

29. Aertgeerts B, Buntinx F, Ansoms S, Fevery J. Screening properties of questionnaires and laboratory tests for the detection of alcohol abuse or dependence in a general practice population. *Br J Gen Pract* 2001; **51**(464): 206-17.

30. Aertgeerts B, Buntinx F, Vandermeulen C, Roelants M, Fevery J, Ansoms S. [Prevalence of alcohol abuse and alcohol dependence according to DSM-IV criteria in first year university students]. *Ned Tijdschr Geneeskd* 1999; **143**(52): 2621-4.

31. World Health Organization Regional Office for Europe (EURO-WHO). Health Behaviour in School-aged Children: WHO Collaborative Cross-National survey/study (HBSC) 2002, 2002.

32. European Monitoring Centre for Drugs and Drug Addiction (EMCDDA). European Monitoring Centre for Drugs and Drug Addiction Statistical Bulletin 2016 Lisbon, Portugal:: European Monitoring Centre for Drugs and Drug Addiction (EMCDDA), 2016.

33. World Health Organization (WHO). Belgium Vital Registration. Deaths ICD10 as it appears in WHO Mortality Database Geneva, Switzerland: World Health Organization (WHO).

34. World Health Organization Regional Office for Europe (WHO/Europe). European Hospital Morbidity Database. Copenhagen, Denmark World Health Organization Regional Office for Europe (WHO/Europe); 2008.

35. Harrison G, Hopper K, Craig T, et al. Recovery from psychotic illness: a 15- and 25-year international follow-up study. *Br J Psychiatry* 2001; **178**: 506-17.

36. Merikangas KR, Jin R, He JP, et al. Prevalence and correlates of bipolar spectrum disorder in the world mental health survey initiative. *Arch Gen Psychiatry* 2011; **68**(3): 241-51.

37. Atilola O, Stevanovic D, Balhara YP, et al. Role of personal and family factors in alcohol and substance use among adolescents: an international study with focus on developing countries. *J Psychiatr Ment Health Nurs* 2014; **21**(7): 609-17.

38. World Health Organization (WHO). WHO World Mental Health Surveys: Global Perspectives on the Epidemiology of Mental Disorders. Cambridge, United Kingdom: Cambridge University Press; 2008.

39. World Health Organization (WHO). Bulgaria Vital Registration. Deaths ICD10 as it appears in WHO Mortality Database Geneva, Switzerland: World Health Organization (WHO).

40. World Health Organization (WHO). Croatia World Health Survey 2003. Geneva, Switzerland: World Health Organization (WHO), 2005.

41. Petkovic G, Barisic I. FAS prevalence in a sample of urban schoolchildren in Croatia. *Reprod Toxicol* 2010; **29**(2): 237-41.

42. Petkovic G, Barisic I. Prevalence of fetal alcohol syndrome and maternal characteristics in a sample of schoolchildren from a rural province of Croatia. *Int J Environ Res Public Health* 2013; **10**(4): 1547-61.

43. European School Survey Project on Alcohol and Other Drugs (ESPAD), Pompidou Group CoE, Swedish Council for Information on Alcohol and Other Drugs (CAN). ESPAD Report 2007: Alcohol and Other Drug Use Among Students in 35 European Countries. Stockholm, Sweden: Swedish Council for Information on Alcohol and Other Drugs (CAN), 2009.

44. World Health Organization (WHO). Croatia Vital Registration. Deaths ICD10 as it appears in WHO Mortality Database Geneva, Switzerland: World Health Organization (WHO).

45. World Health Organization (WHO). Yugoslavia Vital Registration. Deaths ICD10 as it appears in WHO Mortality Database Geneva, Switzerland: World Health Organization (WHO).

46. Sokratis S, Christos Z, Despo P, Maria K. Prevalence of depressive symptoms among schoolchildren in Cyprus: a cross-sectional descriptive correlational study. *Child Adolesc Psychiatry Ment Health* 2017; **11**: 7.

47. Cakici M, Gokce O, Babayigit A, Cakici E, Es A. Depression: point-prevalence and risk factors in a North Cyprus household adult cross-sectional study. *BMC Psychiatry* 2017; **17**(1): 387.

48. Hekim Bozkurt O, Dirik E, Uneri OS. Prevalence of ADHD in Northern Cyprus. *Anadolu Psikiyatr De* 2017; **18**(5): 478-84.

49. European Monitoring Centre for Drugs and Drug Addiction (EMCDDA). Cyprus National Report to the EMCDDA 2004, 2004.

50. World Health Organization (WHO). Cyprus Vital Registration. Deaths ICD10 as it appears in WHO Mortality Database Geneva, Switzerland: World Health Organization (WHO).

51. World Health Organization (WHO). Czech Republic World Health Survey 2002-2003. Geneva, Switzerland: World Health Organization (WHO), 2005.

52. Andrade L, Caraveo-Anduaga JJ, Berglund P, et al. The epidemiology of major depressive episodes: results from the International Consortium of Psychiatric Epidemiology (ICPE) Surveys. *Int J Methods Psychiatr Res* 2003; **12**(1): 3-21.

53. European Monitoring Centre for Drugs and Drug Addiction (EMCDDA). European Monitoring Centre for Drugs and Drug Addiction Statistical Bulletin 2011. Lisbon, Portugal: European Monitoring Centre for Drugs and Drug Addiction (EMCDDA), 2011.

54. Csemy L, Sovinova H, Prochazka B. Alcohol consumption and marijuana use in young adult Czechs. *Cent Eur J Public Health* 2012; **20**(4): 244-7.

55. Czech National Monitoring Centre for Drugs and Drug Addiction. Czech Republic National Report to the EMCDDA 2005. Lisbon, Portugal: European Monitoring Centre for Drugs and Drug Addiction (EMCDDA), 2005.

56. Institute of Health Information and Statistics of the Czech Republic. Czech Republic Health Status Sample Survey 2004.

57. Kazmer L, Dzurova D, Csemy L, Spilkova J. Multiple health risk behaviour in Czech adolescents: family, school and geographic factors. *Health Place* 2014; **29**: 18-25.

58. Lejckova P, Mravcik V. [Mortality of drug users. Summary of cohort study results]. *Epidemiol Mikrobiol Imunol* 2005; **54**(4): 154-60.

59. Lejckova P, Mravcik V. Mortality of hospitalized drug users in the Czech Republic. *J Drug Issues* 2007; **37**(1): 103-18.

60. Sopko B, Skarupova K, Necas V, Mravcik V. Estimation of Problem Drug Users in Prague in 2011 from Low-threshold Data: Modified Capture-Recapture Method, Adjusted for Clients Avoiding Any Identification (Non-Coded Clients). *Cent Eur J Public Health* 2016; **24**(1): 39-44.

61. World Health Organization (WHO). Czechia Vital Registration. Deaths ICD10 as it appears in WHO Mortality Database Geneva, Switzerland: World Health Organization (WHO).

62. World Health Organization (WHO). Czechoslovakia Vital Registration. Deaths ICD10 as it appears in WHO Mortality Database Geneva, Switzerland: World Health Organization (WHO).

63. Munk-Jorgensen P, Weeke A, Jensen EB, Dupont A, Stromgren E. Changes in utilization of Danish psychiatric institutions. II. Census studies 1977 and 1982. *Compr Psychiatry* 1986; **27**(5): 416-29.

64. Okkels N, Vernal DL, Jensen SO, McGrath JJ, Nielsen RE. Changes in the diagnosed incidence of early onset schizophrenia over four decades. *Acta Psychiatr Scand* 2013; **127**(1): 62-8.

65. Bojholm S, Stromgren E. Prevalence of schizophrenia on the island of Bornholm in 1935 and in 1983. *Acta Psychiatr Scand Suppl* 1989; **348**: 157-66; discussion 67-78.

66. Castagnini A, Foldager L. Variations in incidence and age of onset of acute and transient psychotic disorders. *Soc Psychiatry Psychiatr Epidemiol* 2013; **48**(12): 1917-22.

67. Hoye A, Jacobsen BK, Hansen V. Increasing mortality in schizophrenia: are women at particular risk? A follow-up of 1111 patients admitted during 1980-2006 in Northern Norway. *Schizophr Res* 2011; **132**(2-3): 228-32.

68. Nielsen RE, Uggerby AS, Jensen SO, McGrath JJ. Increasing mortality gap for patients diagnosed with schizophrenia over the last three decades--a Danish nationwide study from 1980 to 2010. *Schizophr Res* 2013; **146**(1-3): 22-7.

69. Wesselhoeft R, Heiervang ER, Kragh-Sorensen P, Juul Sorensen M, Bilenberg N. Major depressive disorder and subthreshold depression in prepubertal children from the Danish National Birth Cohort. *Compr Psychiatry* 2016; **70**: 65-76.

70. Laursen TM, Musliner KL, Benros ME, Vestergaard M, Munk-Olsen T. Mortality and life expectancy in persons with severe unipolar depression. *J Affect Disord* 2016; **193**: 203-7.

71. World Health Organization (WHO). Denmark World Health Survey 2003. Geneva, Switzerland: World Health Organization (WHO), 2005.

72. Laursen TM, Munk-Olsen T, Nordentoft M, Mortensen PB. Increased mortality among patients admitted with major psychiatric disorders: a register-based study comparing mortality in unipolar depressive disorder, bipolar affective disorder, schizoaffective disorder, and schizophrenia. *J Clin Psychiatry* 2007; **68**(6): 899-907.

73. Weeke A, Vaeth M. Excess mortality of bipolar and unipolar manic-depressive patients. *J Affect Disord* 1986; **11**(3): 227-34.

74. Jacoby AS, Munkholm K, Vinberg M, et al. Glycogen synthase kinase-3beta in patients with bipolar I disorder: results from a prospective study. *Bipolar Disord* 2016; **18**(4): 334-41.

75. Meier SM, Mattheisen M, Mors O, Mortensen PB, Laursen TM, Penninx BW. Increased mortality among people with anxiety disorders: total population study. *Br J Psychiatry* 2016; **209**(3): 216-21.

76. Helverskov JL, Clausen L, Mors O, Frydenberg M, Thomsen PH, Rokkedal K. Trans-Diagnostic Outcome of Eating Disorders: A 30-Month Follow-Up Study of 629 Patients. *Eur Eat Disord Rev* 2010; **18**(6): 453-63.

77. Stoving RK, Andries A, Brixen K, Bilenberg N, Horder K. Gender differences in outcome of eating disorders: a retrospective cohort study. *Psychiatry Res* 2011; **186**(2-3): 362-6.

78. Clausen L. Time to remission for eating disorder patients: a 2(1/2)-year follow-up study of outcome and predictors. *Nord J Psychiatry* 2008; **62**(2): 151-9.

79. Atladottir HO, Schendel DE, Parner ET, Henriksen TB. A Descriptive Study on the Neonatal Morbidity Profile of Autism Spectrum Disorders, Including a Comparison with Other Neurodevelopmental Disorders. *J Autism Dev Disord* 2015; **45**(8): 2429-42.

80. Ellefsen A, Kampmann H, Billstedt E, Gillberg IC, Gillberg C. Autism in the Faroe Islands: an epidemiological study. *J Autism Dev Disord* 2007; **37**(3): 437-44.

81. Isager T, Mouridsen SE, Rich B. Mortality and Causes of Death in Pervasive Developmental Disorders. *Autism* 1999; **3**(1): 7-16.

82. Li J, Vestergaard M, Obel C, et al. A nationwide study on the risk of autism after prenatal stress exposure to maternal bereavement. *Pediatrics* 2009; **123**(4): 1102-7.

83. Parner ET, Baron-Cohen S, Lauritsen MB, et al. Parental Age and Autism Spectrum Disorders. *Ann Epidemiol* 2012; **22**(3): 143-50.

84. Singer AB, Burstyn I, Thygesen M, Mortensen PB, Fallin MD, Schendel DE. Parental exposures to occupational asthmagens and risk of autism spectrum disorder in a Danish population-based case-control study. *Environ Health* 2017; **16**(1): 31.

85. Vassos E, Agerbo E, Mors O, Pedersen CB. Urban-rural differences in incidence rates of psychiatric disorders in Denmark. *Br J Psychiatry* 2016; **208**(5): 435-40.

86. Kocovska E, Biskupsto R, Carina Gillberg I, et al. The rising prevalence of autism: a prospective longitudinal study in the Faroe Islands. *J Autism Dev Disord* 2012; **42**(9): 1959-66.

87. Dalsgaard S, Ostergaard SD, Leckman JF, Mortensen PB, Pedersen MG. Mortality in children, adolescents, and adults with attention deficit hyperactivity disorder: a nationwide cohort study. *Lancet* 2015; **385**(9983): 2190-6.

88. Scott JG, Giortz Pedersen M, Erskine HE, et al. Mortality in individuals with disruptive behavior disorders diagnosed by specialist services - A nationwide cohort study. *Psychiatry Res* 2017; **251**: 255-60.

89. Arendt M, Munk-Jorgensen P, Sher L, Jensen SO. Mortality among individuals with cannabis, cocaine, amphetamine, MDMA, and opioid use disorders: a nationwide follow-up study of Danish substance users in treatment. *Drug Alcohol Depend* 2011; **114**(2-3): 134-9.

90. Ishoy T, Haastrup L, Hay G. Estimating the prevalence of problem opioid use in Copenhagen 1997-1998. *Dan Med Bull* 2004; **51**(1): 114-6.

91. Sorensen HJ, Jepsen PW, Haastrup S, Juel K. Drug-use pattern, comorbid psychosis and mortality in people with a history of opioid addiction. *Acta Psychiatr Scand* 2005; **111**(3): 244-9.

92. World Health Organization (WHO). Denmark Vital Registration. Deaths ICD10 as it appears in WHO Mortality Database Geneva, Switzerland: World Health Organization (WHO).

93. Kleinberg A, Aluoja A, Vasar V. Point prevalence of major depression in Estonia. Results from the 2006 Estonian Health Survey. *Eur Psychiatry* 2010; **25**(8): 485-90.

94. World Health Organization (WHO). Estonia World Health Survey 2003. Geneva, Switzerland: World Health Organization (WHO), 2005.

95. National Institute for Health Development (Estonia). Estonia Health Behavior Among the Adult Population 2006, 2006.

96. National Institute for Health Development (Estonia). Estonia Health Behavior Among the Adult Population 2010, 2010.

97. National Institute for Health Development (Estonia). Estonia Health Behavior Among the Adult Population 2014, 2014.

98. World Health Organization (WHO). Estonia Vital Registration. Deaths ICD10 as it appears in WHO Mortality Database Geneva, Switzerland: World Health Organization (WHO).

99. Kiviniemi M, Suvisaari J, Pirkola S, Hakkinen U, Isohanni M, Hakko H. Regional differences in five-year mortality after a first episode of schizophrenia in Finland. *Psychiatr Serv* 2010; **61**(3): 272-9.

100. Heila H, Haukka J, Suvisaari J, Lonnqvist J. Mortality among patients with schizophrenia and reduced psychiatric hospital care. *Psychol Med* 2005; **35**(5): 725-32.

101. Lauronen E, Koskinen J, Veijola J, et al. Recovery from schizophrenic psychoses within the northern Finland 1966 Birth Cohort. *J Clin Psychiatry* 2005; **66**(3): 375-83.

102. Ayuso-Mateos JL, Vazquez-Barquero JL, Dowrick C, et al. Depressive disorders in Europe: prevalence figures from the ODIN study. *Br J Psychiatry* 2001; **179**: 308-16.

103. Frojd S, Marttunen M, Pelkonen M, von der Pahlen B, Kaltiala-Heino R. Adult and peer involvement in help-seeking for depression in adolescent population: a two-year follow-up in Finland. *Soc Psychiatry Psychiatr Epidemiol* 2007; **42**(12): 945-52.

104. World Health Organization (WHO). Finland World Health Survey 2004. Geneva, Switzerland: World Health Organization (WHO), 2005.

105. Aalto-Setala T, Marttunen M, Tuulio-Henriksson A, Poikolainen K, Lonnqvist J. One-month prevalence of depression and other DSM-IV disorders among young adults. *Psychol Med* 2001; **31**(5): 791-801.

106. Jylha P, Isometsa E. The relationship of neuroticism and extraversion to symptoms of anxiety and depression in the general population. *Depress Anxiety* 2006; **23**(5): 281-9.

107. Pallaskorpi S, Suominen K, Ketokivi M, et al. Five-year outcome of bipolar I and II disorders: findings of the Jorvi Bipolar Study. *Bipolar Disord* 2015; **17**(4): 363-74.

108. Isomaa R, Isomaa AL, Marttunen M, Kaltiala-Heino R, Bjorkqvist K. The prevalence, incidence and development of eating disorders in Finnish adolescents: a two-step 3-year follow-up study. *Eur Eat Disord Rev* 2009; **17**(3): 199-207.

109. Lahteenmaki S, Saarni S, Suokas J, et al. Prevalence and correlates of eating disorders among young adults in Finland. *Nord J Psychiatry* 2014; **68**(3): 196-203.

110. Backstrom J, Saarijarvi S, Helenius H. The prognosis of Finnish female anorexia patients. *Nord J Psychiat* 1997; **51**(1): 59-60.

111. Keski-Rahkonen A, Hoek HW, Susser ES, et al. Epidemiology and course of anorexia nervosa in the community. *Am J Psychiatry* 2007; **164**(8): 1259-65.

112. Kielinen M, Linna SL, Moilanen I. Autism in Northern Finland. *Eur Child Adolesc Psychiatry* 2000; **9**(3): 162-7.

113. Smalley SL, McGough JJ, Moilanen IK, et al. Prevalence and psychiatric comorbidity of attention-deficit/hyperactivity disorder in an adolescent Finnish population. *J Am Acad Child Adolesc Psychiatry* 2007; **46**(12): 1575-83.

114. Patja K, Molsa P, Iivanainen M. Cause-specific mortality of people with intellectual disability in a population-based, 35-year follow-up study. *J Intellect Disabil Res* 2001; **45**(Pt 1): 30-40.

115. Pirkola SP, Poikolainen K, Lonnqvist JK. Currently active and remitted alcohol dependence in a nationwide adult general population--results from the Finnish Health 2000 study. *Alcohol Alcohol* 2006; **41**(3): 315-20.

116. Poikolainen K. Risk factors for alcohol dependence: a questionnaire survey. *Alcohol Clin Exp Res* 1997; **21**(6): 957-61.

117. European Monitoring Centre for Drugs and Drug Addiction (EMCDDA), National Research and Development Center for Welfare and Health (STAKES) (Finland). Finland National Report to the EMCDDA 2006. Finland: National Research and Development Center for Welfare and Health (STAKES), 2006.

118. Turpeinen P. Outcome of drug abuse in a 20-year follow-up study of drug-experimenting schoolchildren in Finland. *Nord J Psychiatry* 2001; **55**(4): 263-70.

119. World Health Organization (WHO). Finland Vital Registration. Deaths ICD10 as it appears in WHO Mortality Database Geneva, Switzerland: World Health Organization (WHO).

120. Bralet MC, Yon V, Loas G, Noisette C. Mortality in schizophrenia: a 8-year follow-up study in 150 chronic schizophrenics. *Encephale* 2000; **26**(6): 32-41.

121. Casadebaig F, Philippe A. [Mortality in schizophrenic patients. 3 years follow-up of a cohort]. *Encephale* 1999; **25**(4): 329-37.

122. Loas G, Yon V, Marechal V, Decle P. Relationships between subjective or objective symptoms and mortality in schizophrenia: a prospective study on 310 schizophrenic patients with a median follow-up of 8.4 years. *Psychiatry Res* 2011; **185**(1-2): 49-53.

123. Carta MG, Kovess V, Hardoy MC, Morosini P, Murgia S, Carpiniello B. Psychiatric disorders in Sardinian immigrants to Paris: a comparison with Parisians and Sardinians resident in Sardinia. *Soc Psychiatry Psychiatr Epidemiol* 2002; **37**(3): 112-7.

124. Font H, Roelandt JL, Behal H, et al. Prevalence and predictors of no lifetime utilization of mental health treatment among people with mental disorders in France: findings from the 'Mental Health in General Population' (MHGP) survey. *Soc Psychiatry Psychiatr Epidemiol* 2018; **53**(6): 567-76.

125. Weissman MM, Bland RC, Canino GJ, et al. Cross-national epidemiology of major depression and bipolar disorder. *JAMA* 1996; **276**(4): 293-9.

126. World Health Organization (WHO). France World Health Survey 2003. Geneva, Switzerland: World Health Organization (WHO), 2005.

127. Kovess-Masfety V, Briffault X, Sapinho D. Prevalence, risk factors, and use of health care in depression: a survey in a large region of France between 1991 and 2005. *Can J Psychiatry* 2009; **54**(10): 701-9.

128. Pignon B, Geoffroy PA, Thomas P, et al. Prevalence and clinical severity of mood disorders among first-, second- and third-generation migrants. *J Affect Disord* 2017; **210**: 174-80.

129. Mathet F, Martin-Guel C, Maurice-Tison S, Bouvard M-P. [Prevalence of depressive disorders in children and adolescents attending primary care. A survey with the Aquitaine Sentinelle Network]. *Encephale* 2003; **29**(5): 391-400.

130. Husky MM, Mazure CM, Ruffault A, Flahault C, Kovess-Masfety V. Differential Associations Between Excess Body Weight and Psychiatric Disorders in Men and Women. *J Womens Health (Larchmt)* 2018; **27**(2): 183-90.

131. Gueguen J, Godart N, Chambry J, et al. Severe anorexia nervosa in men: comparison with severe AN in women and analysis of mortality. *Int J Eat Disord* 2012; **45**(4): 537-45.

132. Huas C, Caille A, Godart N, et al. Factors predictive of ten-year mortality in severe anorexia nervosa patients. *Acta Psychiatr Scand* 2011; **123**(1): 62-70.

133. Ledoux S, Choquet M, Flament M. Eating Disorders among Adolescents in an Unselected French Population. *Int J Eat Disorder* 1991; **10**(1): 81-9.

134. Aussilloux C, Collery F, Roy J. Epidemiologie de l’autisme infantile dans le departement de le’Herault. *Rev Fr Psychiatr* 1989; **7**(6): 24-8.

135. Fombonne E. The Chartres Study: I. Prevalence of psychiatric disorders among French school-age children. *Br J Psychiatry* 1994; **164**(1): 69-79.

136. Lecendreux M, Konofal E, Cortese S, Faraone SV. A 4-year follow-up of attention-deficit/hyperactivity disorder in a population sample. *J Clin Psychiatry* 2015; **76**(6): 712-9.

137. Serreau R, Maillard T, Verdier R, et al. [Clinical study and prevalence of fetal alcohol syndrome in medico-social institutions of the Reunion Island]. *Arch Pediatr* 2002; **9**(1): 14-20.

138. Melchior M, Chollet A, Elidemir G, Galera C, Younes N. Unemployment and substance use in young adults: does educational attainment modify the association? *Eur Addict Res* 2015; **21**(3): 115-23.

139. Chau K, Baumann M, Chau N. Socioeconomic inequities patterns of multi-morbidity in early adolescence. *Int J Equity Health* 2013; **12**: 65.

140. European School Survey Project on Alcohol and Other Drugs (ESPAD), Pompidou Group CoE, Swedish Council for Information on Alcohol and Other Drugs (CAN). Alcohol and Drug Use Among European 17-18 Year Old Students: Data from the ESPAD Project Stockholm, Sweden Swedish Council for Information on Alcohol and Other Drugs (CAN), 2017.

141. European Monitoring Centre for Drugs and Drug Addiction (EMCDDA), French Monitoring Centre for Drugs and Drug Addiction. France National Report to the EMCDDA 2006. Lisbon, Portugal: European Monitoring Centre for Drugs and Drug Addiction (EMCDDA), 2006.

142. French Monitoring Centre for Drugs and Drug Addiction. France Reincarceration and Mortality Among Opioid-Dependent Prisoners Study 2003-2006.

143. Lopez D, Martineau H, Palle C. Mortality of individuals arrested for heroin, cocaine or crack use. *Tendances* 2004: 1-8.

144. Melchior M, Chastang JF, Goldberg P, Fombonne E. High prevalence rates of tobacco, alcohol and drug use in adolescents and young adults in France: results from the GAZEL Youth study. *Addict Behav* 2008; **33**(1): 122-33.

145. Yaogo A, Fombonne E, Lert F, Melchior M. Adolescent Repeated Alcohol Intoxication as a Predictor of Young Adulthood Alcohol Abuse: The Role of Socioeconomic Context. *Subst Use Misuse* 2015; **50**(14): 1795-804.

146. World Health Organization (WHO). France Vital Registration. Deaths ICD10 as it appears in WHO Mortality Database Geneva, Switzerland: World Health Organization (WHO).

147. Fichter MM, Narrow WE, Roper MT, et al. Prevalence of mental illness in Germany and the United States - Comparison of the Upper Bavarian Study and the Epidemiologic Catchment Area Program. *J Nerv Ment Dis* 1996; **184**(10): 598-606.

148. Hewer W, Rossler W. [Mortality of patients with functional psychiatric illnesses during inpatient treatment]. *Fortschr Neurol Psychiatr* 1997; **65**(4): 171-81.

149. Bettge S, Wille N, Barkmann C, Schulte-Markwort M, Ravens-Sieberer U, group Bs. Depressive symptoms of children and adolescents in a German representative sample: results of the BELLA study. *Eur Child Adolesc Psychiatry* 2008; **17 Suppl 1**: 71-81.

150. Ravens-Sieberer U, Wille N, Erhart M, et al. Prevalence of mental health problems among children and adolescents in Germany: results of the BELLA study within the National Health Interview and Examination Survey. *Eur Child Adolesc Psychiatry* 2008; **17 Suppl 1**: 22-33.

151. Asselmann E, Hilbert K, Hoyer J, et al. Self-reported volitional control in adolescents and young adults from a community cohort: Associations with current, past and future mental disorders. *Psychiatry Res* 2018; **260**: 292-9.

152. Jacobi F, Hofler M, Strehle J, et al. Twelve-months prevalence of mental disorders in the German Health Interview and Examination Survey for Adults - Mental Health Module (DEGS1-MH): a methodological addendum and correction. *Int J Methods Psychiatr Res* 2015; **24**(4): 305-13.

153. Maske UE, Buttery AK, Beesdo-Baum K, Riedel-Heller S, Hapke U, Busch MA. Prevalence and correlates of DSM-IV-TR major depressive disorder, self-reported diagnosed depression and current depressive symptoms among adults in Germany. *J Affect Disord* 2016; **190**: 167-77.

154. Wittchen HU, Nelson CB, Lachner G. Prevalence of mental disorders and psychosocial impairments in adolescents and young adults. *Psychol Med* 1998; **28**(1): 109-26.

155. World Health Organization (WHO). Germany World Health Survey 2004. Geneva, Switzerland: World Health Organization (WHO), 2006.

156. Baune BT, Adrian I, Arolt V, Berger K. Associations between major depression, bipolar disorders, dysthymia and cardiovascular diseases in the general adult population. *Psychother Psychosom* 2006; **75**(5): 319-26.

157. Schneider B, Muller MJ, Philipp M. Mortality in affective disorders. *J Affect Disord* 2001; **65**(3): 263-74.

158. Meyer C, Rumpf HJ, Hapke U, John U. Prevalence of DSM-IV psychiatric disorders including nicotine dependence in the general population: Results from the Northern German TACOS study. *Neurol Psychiat Br* 2001; **9**(2): 75-80.

159. Lukat J, Becker ES, Lavallee KL, van der Veld WM, Margraf J. Predictors of Incidence, Remission and Relapse of Axis I Mental Disorders in Young Women: A Transdiagnostic Approach. *Clin Psychol Psychother* 2017; **24**(2): 322-31.

160. Bogh EH, Rokkedal K, Valbak K. A 4-year follow-up on bulimia nervosa. *Eur Eat Disord Rev* 2005; **13**(1): 48-53.

161. Fichter MM, Quadflieg N, Hedlund S. Twelve-year course and outcome predictors of anorexia nervosa. *Int J Eat Disord* 2006; **39**(2): 87-100.

162. Zipfel S, Lowe B, Reas DL, Deter HC, Herzog W. Long-term prognosis in anorexia nervosa: lessons from a 21-year follow-up study. *Lancet* 2000; **355**(9205): 721-2.

163. Dopfner M, Breuer D, Wille N, Erhart M, Ravens-Sieberer U, group Bs. How often do children meet ICD-10/DSM-IV criteria of attention deficit-/hyperactivity disorder and hyperkinetic disorder? Parent-based prevalence rates in a national sample--results of the BELLA study. *Eur Child Adolesc Psychiatry* 2008; **17 Suppl 1**: 59-70.

164. Essau CA, Groen G, Conradt J, Turbanisch U, Petermann F. [Frequency, comorbidity and psychosocial correlates of attention-deficit/hyperactivity disorder. Results of a Bremen adolescent study]. *Fortschr Neurol Psychiatr* 1999; **67**(7): 296-305.

165. Weyerer S, Castell R, Biener A, Artner K, Dilling H. Prevalence and treatment of psychiatric disorders in 3 to 14-year-old children: results of a representative field study in the small town rural region of Traunstein, upper Bavaria. *Acta Psychiatr Scand* 1988; **77**(3): 290-6.

166. Nelson CB, Wittchen HU. DSM-IV alcohol disorders in a general population sample of adolescents and young adults. *Addiction* 1998; **93**(7): 1065-77.

167. Dybek I, Bischof G, Grothues J, et al. The reliability and validity of the alcohol use disorders identification test (AUDIT) in a German general practice population sample. *J Stud Alcohol* 2006; **67**(3): 473-81.

168. Federal Ministry of Health (Germany), Institute for Therapy and Health Research (IFT). Germany Population Survey on the Consumption of Psychoative Substances in the German Adult Population 1997.

169. Federal Ministry of Health (Germany), Institute for Therapy and Health Research (IFT). Germany Population Survey on the Consumption of Psychoative Substances in the German Adult Population 2000.

170. Hohne B, Pabst A, Hannemann TV, Kraus L. Patterns of concurrent alcohol, tobacco, and cannabis use in Germany: Prevalence and correlates. *Drug-Educ Prev Polic* 2014; **21**(2): 102-9.

171. Jacobi F, Wittchen HU, Holting C, et al. Prevalence, co-morbidity and correlates of mental disorders in the general population: results from the German Health Interview and Examination Survey (GHS). *Psychol Med* 2004; **34**(4): 597-611.

172. Pabst A, Kraus L, Piontek D, Baumeister SE. Age differences in diagnostic criteria of DSM-IV alcohol dependence among adults with similar drinking behaviour. *Addiction* 2012; **107**(2): 331-8.

173. Rumpf HJ, Hapke U, Meyer C, John U. Screening for alcohol use disorders and at-risk drinking in the general population: Psychometric performance of three questionnaires. *Alcohol Alcoholism* 2002; **37**(3): 261-8.

174. Perkonigg A, Goodwin RD, Fiedler A, et al. The natural course of cannabis use, abuse and dependence during the first decades of life. *Addiction* 2008; **103**(3): 439-49.

175. Augustin R, Kraus L. Changes in prevalence of problem opiate use in Germany between 1990 and 2000. *European Addiction Research* 2004; **10**(2): 61-7.

176. European Monitoring Centre for Drugs and Drug Addiction (EMCDDA). Germany National Report to the EMCDDA 2005. Lisbon, Portugal: European Monitoring Centre for Drugs and Drug Addiction (EMCDDA), 2005.

177. Kraus L, Pabst A, Piontek D, Gomes de Matos E. Substanzkonsum und substanzbezogene Storungen: Trends in Deutschland 1980-2012 [Substance use and substance use disorders: Trends in Germany 1980-2012]. *Sucht* 2013; **59**: 333-45.

178. Pabst A, Kraus L, Matos EG de, Piontek D. Substanzkonsum und substanzbezogene Störungen in Deutschland im Jahr 2012. *Sucht* 2013; **59**: 321-31.

179. Piontek D, Kraus L, Pabst A, Legleye S. An age-period-cohort analysis of cannabis use prevalence and frequency in Germany, 1990-2009. *J Epidemiol Commun H* 2012; **66**(10): 908-13.

180. Scherbaum N, Specka M, Hauptmann G, Gastpar M. [Does maintenance treatment reduce the mortality rate of opioid addicts?]. *Fortschr Neurol Psychiatr* 2002; **70**: 455-61.

181. Soyka M, Apelt SM, Lieb M, Wittchen HU. One-year mortality rates of patients receiving methadone and buprenorphine maintenance therapy: a nationally representative cohort study in 2694 patients. *J Clin Psychopharmacol* 2006; **26**(6): 657-60.

182. World Health Organization (WHO). Germany Vital Registration. Deaths ICD10 as it appears in WHO Mortality Database Geneva, Switzerland: World Health Organization (WHO).

183. Economou M, Madianos M, Peppou LE, Patelakis A, Stefanis CN. Major depression in the era of economic crisis: a replication of a cross-sectional study across Greece. *J Affect Disord* 2013; **145**(3): 308-14.

184. Madianos MG, Stefanis CN. Changes in the prevalence of symptoms of depression and depression across Greece. *Soc Psychiatry Psychiatr Epidemiol* 1992; **27**(5): 211-9.

185. Skapinakis P, Bellos S, Koupidis S, Grammatikopoulos I, Theodorakis PN, Mavreas V. Prevalence and sociodemographic associations of common mental disorders in a nationally representative sample of the general population of Greece. *BMC Psychiatry* 2013; **13**: 163.

186. Stylianidis S, Pantelidou S, Chondros P, Roelandt JL, Barbato A. Prevalence of mental disorders in a Greek island. *Psychiatriki* 2014; **25**(1): 19-26.

187. World Health Organization (WHO). Greece World Health Survey 2003. Geneva, Switzerland: World Health Organization (WHO), 2005.

188. Fichter MM, Quadflieg N, Georgopoulou E, Xepapadakos F, Fthenakis EW. Time trends in eating disturbances in young Greek migrants. *Int J Eat Disord* 2005; **38**(4): 310-22.

189. Skounti M, Giannoukas S, Dimitriou E, Nikolopoulou S, Linardakis E, Philalithis A. Prevalence of attention deficit hyperactivity disorder in schoolchildren in Athens, Greece. Association of ADHD subtypes with social and academic impairment. *Atten Defic Hyperact Disord* 2010; **2**(3): 127-32.

190. European Monitoring Centre for Drugs and Drug Addiction (EMCDDA). Greece National Report to the EMCDDA 2006. Lisbon, Portugal: European Monitoring Centre for Drugs and Drug Addiction (EMCDDA), 2006.

191. Kokkevi A, Fotiou A, Richardson C. Drug use in the general population of Greece over the last 20 years: Results from nationwide household surveys. *European Addiction Research* 2007; **13**(3): 167-76.

192. World Health Organization (WHO). Greece Vital Registration. Deaths ICD10 as it appears in WHO Mortality Database Geneva, Switzerland: World Health Organization (WHO).

193. World Health Organization (WHO). Ireland World Health Survey 2003. Geneva, Switzerland: World Health Organization (WHO), 2005.

194. Tolgyes T, Nemessury J. Epidemiological studies on adverse dieting behaviours and eating disorders among young people in Hungary. *Soc Psychiatry Psychiatr Epidemiol* 2004; **39**(8): 647-54.

195. Szumska I, Tury F, Csoboth CT, Rethelyi J, Purebl G, Hajnal A. The prevalence of eating disorders and weight-control methods among young women: A Hungarian representative study. *Eur Eat Disord Rev* 2005; **13**(4): 278-84.

196. Bitter I, Simon V, Balint S, Meszaros A, Czobor P. How do different diagnostic criteria, age and gender affect the prevalence of attention deficit hyperactivity disorder in adults? An epidemiological study in a Hungarian community sample. *Eur Arch Psychiatry Clin Neurosci* 2010; **260**(4): 287-96.

197. World Health Organization Regional Office for Europe (WHO/Europe). Young People's Health in Context. Health Behaviour in School-aged Children (HBSC) Study: International Report from the 2001-2002 Survey. . Copenhagen, Denmark: World Health Organization Regional Office for Europe (WHO/Europe), 2004.

198. World Health Organization (WHO). Hungary Vital Registration. Deaths ICD10 as it appears in WHO Mortality Database Geneva, Switzerland: World Health Organization (WHO).

199. Helgason T. Epidemiology of Mental Disorders in Iceland. A Psychiatric and Demographic Investigation of 5395 Icelanders. *Acta Psychiatr Scand* 1964; **40**: SUPPL 173:1+.

200. Jonsdottir SL, Saemundsen E, Antonsdottir IS, Sigurdardottir S, Olason D. Children diagnosed with autism spectrum disorder before or after the age of 6 years. *Res Autism Spect Dis* 2011; **5**(1): 175-84.

201. Magnusson P, Saemundsen E. Prevalence of autism in Iceland. *J Autism Dev Disord* 2001; **31**(2): 153-63.

202. Saemundsen E, Magnusson P, Georgsdottir I, Egilsson E, Rafnsson V. Prevalence of autism spectrum disorders in an Icelandic birth cohort. *BMJ Open* 2013; **3**(6).

203. Thorarinsson AA. Mortality among men alcoholics in Iceland, 1951--74. *J Stud Alcohol* 1979; **40**(7): 704-18.

204. World Health Organization Regional Office for Europe (EURO-WHO). Health Behaviour in School-aged Children: WHO Collaborative Cross-National survey/study (HBSC) 2009-2010, 2010.

205. Directorate of Health (Iceland). Iceland Causes of Death Register

206. World Health Organization (WHO). Iceland Vital Registration. Deaths ICD10 as it appears in WHO Mortality Database Geneva, Switzerland: World Health Organization (WHO).

207. Harley ME, Connor D, Clarke MC, et al. Prevalence of Mental Disorder among young adults in Ireland: a population based study. *Ir J Psychol Med* 2015; **32**(1): 79-91.

208. Pringle DG, Waddington JL, Youssef HA Schizophrenia in East County Cavan: spatial variations in prevalence and their aetiological implications. *Irish Geogr* 1995; **28**(1): 1-13.

209. Youssef HA, Scully PJ, Kinsella A, Waddington JL. Geographical variation in rate of schizophrenia in rural Ireland by place at birth vs place at onset. *Schizophr Res* 1999; **37**(3): 233-43.

210. Morgan MG, Scully PJ, Youssef HA, Kinsella A, Owens JM, Waddington JL. Prospective analysis of premature mortality in schizophrenia in relation to health service engagement: a 7.5-year study within an epidemiologically complete, homogeneous population in rural Ireland. *Psychiatry Res* 2003; **117**(2): 127-35.

211. Coughlan H, Tiedt L, Clarke M, et al. Prevalence of DSM-IV mental disorders, deliberate self-harm and suicidal ideation in early adolescence: an Irish population-based study. *J Adolesc* 2014; **37**(1): 1-9.

212. Economic and Social Research Institute (ESRI) (Ireland), Health Promotion Unit DoHaCI, National University of Ireland G, Royal College of Surgeons in Ireland (RCSI), Cork UC. Survey of Lifestyle Attitudes and Nutrition 2007. Dublin, Ireland: Health Promotion Unit, Department of Health and Children (Ireland).

213. Bunting B, Murphy S, O'Neill S, Ferry F. Prevalence and treatment of 12-month DSM-IV disorders in the Northern Ireland study of health and stress. *Soc Psychiatry Psychiatr Epidemiol* 2013; **48**(1): 81-93.

214. Scully PJ, Owens JM, Kinsella A, Waddington JL. Schizophrenia, schizoaffective and bipolar disorder within an epidemiologically complete, homogeneous population in rural Ireland: small area variation in rate. *Schizophr Res* 2004; **67**(2-3): 143-55.

215. Lynch F, Mills C, Daly I, Fitzpatrick C. Challenging times: prevalence of psychiatric disorders and suicidal behaviours in Irish adolescents. *J Adolesc* 2006; **29**(4): 555-73.

216. Boilson AM, Staines A, Ramirez A, Posada M, Sweeney MR. Operationalisation of the European Protocol for Autism Prevalence (EPAP) for Autism Spectrum Disorder Prevalence Measurement in Ireland. *J Autism Dev Disord* 2016; **46**(9): 3054-67.

217. Cousins G, Mongan D, Barry J, Smyth B, Rackard M, Long J. Estimating Risk of Alcohol Dependence Using Empirically Validated Ordinal Risk Zones Versus Recommended Binary Risk Zones of the RAPS4: A Validation Study Using Stratum-Specific Likelihood Ratio Analysis. *Alcohol Clin Exp Res* 2016; **40**(8): 1700-6.

218. Centre for Health Promotion Studies NUoI, Galway, , Department of Public Health Medicine and Epidemiology UCD, Health Promotion Unit DoHaCI. Ireland National Health and Lifestyle Surveys Report 1998-2002. Dublin, Ireland: Health Promotion Unit, Department of Health and Children (Ireland), 2003.

219. Department of Health SSaPSNI, National Advisory Committee on Drugs (Ireland). Ireland and Northern Ireland Drug Prevalence Survey 2002-2003.

220. Department of Health SSaPSNI, National Advisory Committee on Drugs (Ireland). Ireland and Northern Ireland Drug Prevalence Survey 2006-2007.

221. Kelly A, Carvalho M, Teljeur C. A 3-Source Capture Recapture Study of the Prevalence of Opiate Use in Ireland 2000 to 2001. Dublin, Ireland: Department of Community Health and General Practice, Trinity College Dublin, 2003.

222. Smyth BP, Barry J, Lane A, et al. In-patient treatment of opiate dependence: medium-term follow-up outcomes. *Br J Psychiatry* 2005; **187**: 360-5.

223. World Health Organization (WHO). Ireland Vital Registration. Deaths ICD10 as it appears in WHO Mortality Database Geneva, Switzerland: World Health Organization (WHO).

224. World Health Organization (WHO). Italy World Health Survey 2003. Geneva, Switzerland: World Health Organization (WHO), 2005.

225. Faravelli C, Degl'innocenti BG, Aiazzi L, Incerpi G, Pallanti S. Epidemiology of Mood Disorders - a Community Survey in Florence. *J Affect Disorders* 1990; **20**(2): 135-41.

226. Donfrancesco R, Marano A, Calderoni D, et al. Prevalence of severe ADHD: an epidemiological study in the Italian regions of Tuscany and Latium. *Epidemiol Psychiatr Sci* 2015; **24**(6): 525-33.

227. European Monitoring Centre for Drugs and Drug Addiction (EMCDDA). Italy National Report to the EMCDDA 2000. Lisbon, Portugal: European Monitoring Centre for Drugs and Drug Addiction (EMCDDA), 2000.

228. European Monitoring Centre for Drugs and Drug Addiction (EMCDDA). Luxembourg National Report to the EMCDDA 2007. Lisbon, Portugal: European Monitoring Centre for Drugs and Drug Addiction (EMCDDA), 2007.

229. Ferri M, Bargagli AM, Faggiano F, et al. Mortality of drug users attending Public Treatment Centers in Italy 1998-2001: a cohort study. *Epidemiol Prev* 2007; **31**(5): 276-82.

230. Manfredi R, Sabbatani S, Agostini D. Trend of mortality observed in a cohort of drug addicts of the metropolitan area of Bologna, North-Eastern Italy, during a 25-year-period. *Collegium Antropol* 2006; **30**(3): 479-88.

231. Zuccato E, Castiglioni S, Senta I, et al. Population surveys compared with wastewater analysis for monitoring illicit drug consumption in Italy in 2010-2014. *Drug Alcohol Depen* 2016; **161**: 178-88.

232. National Institute of Statistics (Italy). Italy Vital Registration - Deaths. Rome, Italy: National Institute of Statistics (Italy).

233. Rancans E, Vrublevska J, Snikere S, Koroleva I, Trapencieris M. The point prevalence of depression and associated sociodemographic correlates in the general population of Latvia. *J Affect Disord* 2014; **156**: 104-10.

234. Vrublevska J, Trapencieris M, Snikere S, et al. The 12-month prevalence of depression and health care utilization in the general population of Latvia. *J Affect Disord* 2017; **210**: 204-10.

235. World Health Organization (WHO). Latvia World Health Survey 2003. Geneva, Switzerland: World Health Organization (WHO), 2005.

236. World Health Organization (WHO). Latvia Vital Registration. Deaths ICD10 as it appears in WHO Mortality Database Geneva, Switzerland: World Health Organization (WHO).

237. Lesinskiene S, Girdzijauskiene S, Gintiliene G, et al. Epidemiological study of child and adolescent psychiatric disorders in Lithuania. *Bmc Public Health* 2018; **18**.

238. Astrauskiene A, Dobrovolskij V, Stukas R. The prevalence of problem drug use in lithuania. *Medicina (Kaunas)* 2011; **47**(6): 340-6.

239. World Health Organization (WHO). Lithuania Vital Registration. Deaths ICD10 as it appears in WHO Mortality Database Geneva, Switzerland: World Health Organization (WHO).

240. World Health Organization (WHO). Luxembourg World Health Survey 2003. Geneva, Switzerland: World Health Organization (WHO), 2005.

241. World Health Organization (WHO). Luxembourg Vital Registration. Deaths ICD10 as it appears in WHO Mortality Database Geneva, Switzerland: World Health Organization (WHO).

242. Bijl RV, Ravelli A, van Zessen G. Prevalence of psychiatric disorder in the general population: results of The Netherlands Mental Health Survey and Incidence Study (NEMESIS). *Soc Psychiatry Psychiatr Epidemiol* 1998; **33**(12): 587-95.

243. Hodiamont P, Peer N, Syben N. Epidemiologic Aspects of Psychiatric-Disorder in a Dutch Health Area. *Psychological Medicine* 1987; **17**(2): 495-505.

244. Hogerzeil SJ, van Hemert AM, Rosendaal FR, Susser E, Hoek HW. Direct comparison of first-contact versus longitudinal register-based case finding in the same population: early evidence that the incidence of schizophrenia may be three times higher than commonly reported. *Psychol Med* 2014; **44**(16): 3481-90.

245. Verhulst FC, van der Ende J, Ferdinand RF, Kasius MC. The prevalence of DSM-III-R diagnoses in a national sample of Dutch adolescents. *Arch Gen Psychiatry* 1997; **54**(4): 329-36.

246. de Graaf R, ten Have M, van Gool C, van Dorsselaer S. Prevalence of mental disorders and trends from 1996 to 2009. Results from the Netherlands Mental Health Survey and Incidence Study-2. *Soc Psychiatry Psychiatr Epidemiol* 2012; **47**(2): 203-13.

247. Ormel J, Raven D, van Oort F, et al. Mental health in Dutch adolescents: a TRAILS report on prevalence, severity, age of onset, continuity and co-morbidity of DSM disorders. *Psychol Med* 2015; **45**(2): 345-60.

248. Spinhoven P, van Hemert AM, Penninx BW. Repetitive negative thinking as a predictor of depression and anxiety: A longitudinal cohort study. *J Affect Disord* 2018; **241**: 216-25.

249. Trimbos Institute (Netherlands) Netherlands Mental Health Survey and Incidence Study 1996.

250. World Health Organization (WHO). Netherlands World Health Survey 2004. Geneva, Switzerland: World Health Organization (WHO), 2005.

251. Bijl RV, De Graaf R, Ravelli A, et al. Gender and age-specific first incidence of DSM-III-R psychiatric disorders in the general population. Results from the Netherlands Mental Health Survey and Incidence Study (NEMESIS). *Soc Psychiatry Psychiatr Epidemiol* 2002; **37**(8): 372-9.

252. Hendriks SM, Spijker J, Licht CM, Beekman AT, Penninx BW. Two-year course of anxiety disorders: different across disorders or dimensions? *Acta Psychiatr Scand* 2013; **128**(3): 212-21.

253. Wang Y, Henriksen CA, Ten Have M, et al. Common Mental Disorder Diagnosis and Need for Treatment are Not the Same: Findings from the NEMESIS Study. *Adm Policy Ment Health* 2016.

254. van Son GE, van Hoeken D, Bartelds AI, van Furth EF, Hoek HW. Time trends in the incidence of eating disorders: a primary care study in the Netherlands. *Int J Eat Disord* 2006; **39**(7): 565-9.

255. Smink FR, van Hoeken D, Oldehinkel AJ, Hoek HW. Prevalence and severity of DSM-5 eating disorders in a community cohort of adolescents. *Int J Eat Disord* 2014; **47**(6): 610-9.

256. Roelfsema MT, Hoekstra RA, Allison C, et al. Are autism spectrum conditions more prevalent in an information-technology region? A school-based study of three regions in the Netherlands. *J Autism Dev Disord* 2012; **42**(5): 734-9.

257. Bijl RV, van Zessen G, Ravelli A, de Rijk C, Langendoen Y. The Netherlands Mental Health Survey and Incidence Study (NEMESIS): objectives and design. *Soc Psychiatry Psychiatr Epidemiol* 1998; **33**(12): 581-6.

258. de Bruijn C, van den Brink W, de Graaf R, Vollebergh WAM. The three year course of alcohol use disorders in the general population: DSM-IV, ICD-10 and the Craving Withdrawal Model. *Addiction* 2006; **101**(3): 385-92.

259. de Graaf R, ten Have M, Tuithof M, van Dorsselaer S. First-incidence of DSM-IV mood, anxiety and substance use disorders and its determinants: results from the Netherlands Mental Health Survey and Incidence Study-2. *J Affect Disord* 2013; **149**(1-3): 100-7.

260. Tiemens B, van den Brink W, van der Meer K, Ormel J. Results from the Groningen Centre. In: TB Ü, N S, eds. Mental Illness in General Health Care: An International Study. Chichester, United Kingdom: John Wiley & Son, Inc; 1995: 137–50.

261. World Health Organization Regional Office for Europe (EURO-WHO). Health Behaviour in School-aged Children: WHO Collaborative Cross-National survey/study (HBSC) 2001-2002, 2001.

262. World Health Organization Regional Office for Europe (EURO-WHO). Health Behaviour in School-aged Children: WHO Collaborative Cross-National survey/study (HBSC) 2005, 2005.

263. World Health Organization Regional Office for Europe (EURO-WHO). Health Behaviour in School-aged Children: WHO Collaborative Cross-National survey/study (HBSC) 2009, 2009.

264. Centre for Drug Research UoA, Netherlands Amsterdam Licit and Illicit Drug Use Survey 1994. 1994.

265. Centre for Drug Research UoA, Netherlands Licit and Illicit Drug Use Survey 1997. 1997.

266. Monshouwer K, Van Dorsselaer S, Verdurmen J, Ter Bogt T, De Graaf R, Vollebergh W. Cannabis use and mental health in secondary school children. Findings from a Dutch survey. *Brit J Psychiat* 2006; **188**: 148-53.

267. Smit F, Monshouwer K, Verdurmen J. Polydrug use among secondary school students: Combinations, prevalences and risk profiles. *Drug-Educ Prev Polic* 2002; **9**(4): 355-65.

268. Verhagen CE, Uitenbroek DG, Schreuders EJ, El Messaoudi S, de Kroon ML. Does a reduction in alcohol use by Dutch high school students relate to higher use of tobacco and cannabis? *Bmc Public Health* 2015; **15**: 821.

269. Abraham MD, Kaal HL, Cohen PDA, Centre for Drug Research UoA. Licit and Illicit Drug Use in Amsterdam, 1987-2001. Amsterdam, Netherlands: Centre for Drug Research, University of Amsterdam, 2002.

270. Buster MC, van Brussel GH, van den Brink W. Estimating the number of opiate users in amsterdam by capture-recapture: the importance of case definition. *Eur J Epidemiol* 2001; **17**(10): 935-42.

271. European Monitoring Centre for Drugs and Drug Addiction (EMCDDA). Netherlands National Report to the EMCDDA 2006, 2006.

272. Korf DJ, Reijneveld SA, Toet J. Estimating the number of heroin users: a review of methods and empirical findings from The Netherlands. *Int J Addict* 1994; **29**(11): 1393-417.

273. Langendam MW, van Brussel GHA, Coutinho RA, van Ameijden EJC. The impact of harm-reduction-based methadone treatment on mortality among heroin users. *Am J Public Health* 2001; **91**(5): 774-80.

274. Perez AO, Cruyff MJLF, Benschop A, Koff DJ. Estimating the Prevalence of Crack Dependence Using Capture-Recapture With Institutional and Field Data: A Three-City Study in the Netherlands. *Substance Use & Misuse* 2013; **48**(1-2): 173-80.

275. Trimbos Institute (Netherlands). Netherlands National Drug Monitor Annual Report 2006, 2006.

276. van Haastrecht HJ, van Ameijden EJ, van den Hoek JA, Mientjes GH, Bax JS, Coutinho RA. Predictors of mortality in the Amsterdam cohort of human immunodeficiency virus (HIV)-positive and HIV-negative drug users. *Am J Epidemiol* 1996; **143**(4): 380-91.

277. World Health Organization (WHO). Netherlands Vital Registration. Deaths ICD10 as it appears in WHO Mortality Database Geneva, Switzerland: World Health Organization (WHO).

278. Consumer Safety Institute (Netherlands). Netherlands Injury Surveillance System.

279. Evensen S, Wisloff T, Lystad JU, Bull H, Ueland T, Falkum E. Prevalence, Employment Rate, and Cost of Schizophrenia in a High-Income Welfare Society: A Population-Based Study Using Comprehensive Health and Welfare Registers. *Schizophr Bull* 2016; **42**(2): 476-83.

280. Nesvag R, Knudsen GP, Bakken IJ, et al. Substance use disorders in schizophrenia, bipolar disorder, and depressive illness: a registry-based study. *Soc Psychiatry Psychiatr Epidemiol* 2015; **50**(8): 1267-76.

281. Heiervang E, Stormark KM, Lundervold AJ, et al. Psychiatric disorders in Norwegian 8- to 10-year-olds: an epidemiological survey of prevalence, risk factors, and service use. *J Am Acad Child Adolesc Psychiatry* 2007; **46**(4): 438-47.

282. Brunes A. The Association Between Physical Activity, Mental Health, and Personality: The HUNT Study. Trondheim, Norway: Norwegian University of Science and Technology; 2011.

283. Johannessen EL, Andersson HW, Bjorngaard JH, Pape K. Anxiety and depression symptoms and alcohol use among adolescents - a cross sectional study of Norwegian secondary school students. *Bmc Public Health* 2017; **17**(1): 494.

284. Kringlen E, Torgersen S, Cramer V. A Norwegian psychiatric epidemiological study. *Am J Psychiatry* 2001; **158**(7): 1091-8.

285. Sund AM, Larsson B, Wichstrom L. Prevalence and characteristics of depressive disorders in early adolescents in central Norway. *Child Adolesc Psychiatry Ment Health* 2011; **5**: 28.

286. von Soest T, Wichstrom L. Secular trends in depressive symptoms among Norwegian adolescents from 1992 to 2010. *J Abnorm Child Psychol* 2014; **42**(3): 403-15.

287. World Health Organization (WHO). Norway World Health Survey 2003. Geneva, Switzerland: World Health Organization (WHO), 2005.

288. Gustavson K, Knudsen AK, Nesvag R, Knudsen GP, Vollset SE, Reichborn-Kjennerud T. Prevalence and stability of mental disorders among young adults: findings from a longitudinal study. *Bmc Psychiatry* 2018; **18**.

289. Kringlen E, Torgersen S, Cramer V. Mental illness in a rural area: a Norwegian psychiatric epidemiological study. *Soc Psychiatry Psychiatr Epidemiol* 2006; **41**(9): 713-9.

290. Askim A, Gustad LT, Paulsen J, et al. Anxiety and Depression Symptoms in a General Population and Future Risk of Bloodstream Infection: The HUNT Study. *Psychosom Med* 2018; **80**(7): 673-9.

291. Sandanger I, Nygard JF, Ingebrigtsen G, Sorensen T, Dalgard OS. Prevalence, incidence and age at onset of psychiatric disorders in Norway. *Soc Psychiatry Psychiatr Epidemiol* 1999; **34**(11): 570-9.

292. Gotestam KG, Agras WS. General population-based epidemiological study of eating disorders in Norway. *Int J Eat Disord* 1995; **18**(2): 119-26.

293. Nilsen RM, Suren P, Gunnes N, et al. Analysis of self-selection bias in a population-based cohort study of autism spectrum disorders. *Paediatr Perinat Epidemiol* 2013; **27**(6): 553-63.

294. Suren P, Bakken IJ, Aase H, et al. Autism spectrum disorder, ADHD, epilepsy, and cerebral palsy in Norwegian children. *Pediatrics* 2012; **130**(1): e152-8.

295. Rossow I, Hawton K, Ystgaard M. Cannabis Use and Deliberate Self-Harm in Adolescence: A Comparative Analysis of Associations in England and Norway. *Arch Suicide Res* 2009; **13**(4): 340-8.

296. Eskild A, Magnus P, Samuelsen SO, Sohlberg C, Kittelsen P. Differences in mortality rates and causes of death between HIV positive and HIV negative intravenous drug users. *Int J Epidemiol* 1993; **22**(2): 315-20.

297. Haarr D, Nessa J. [Treatment of opiate-dependent patients in a general practice]. *Tidsskr Nor Laegeforen* 2007; **127**(13): 1770-2.

298. Odegard E, Amundsen EJ, Kielland KB. Fatal overdoses and deaths by other causes in a cohort of Norwegian drug abusers - A competing risk approach. *Drug Alcohol Depen* 2007; **89**(2-3): 176-82.

299. Rossow I. Suicide among Drug-Addicts in Norway. *Addiction* 1994; **89**(12): 1667-73.

300. Clausen T, Anchersen K, Waal H. Mortality prior to, during and after opioid maintenance treatment (OMT): a national prospective cross-registry study. *Drug Alcohol Depend* 2008; **94**(1-3): 151-7.

301. Health NIoP. Norway Cause of Death Registry. Oslo, Norway: Norwegian Institute of Public Health**,**.

302. Kessler RC, Sampson NA, Berglund P, et al. Anxious and non-anxious major depressive disorder in the World Health Organization World Mental Health Surveys. *Epidemiol Psychiatr Sci* 2015; **24**(3): 210-26.

303. Siwek M, Styczen K, Sowa-Kucma M, et al. The serum concentration of magnesium as a potential state marker in patients with diagnosis of bipolar disorder. *Psychiatr Pol* 2015; **49**(6): 1277-87.

304. Cherpitel CJ, Ye Y, Moskalewicz J, Swiatkiewicz G. Screening for alcohol problems in two emergency service samples in Poland: comparison of the RAPS4, CAGE and AUDIT. *Drug Alcohol Depend* 2005; **80**(2): 201-7.

305. National Bureau for Drug Prevention (Poland). Poland National Report to the EMCDDA 2006, 2006.

306. Central Statistical Office (Poland), National Institute of Public Health-National Institute of Hygiene (NIPH-NIH) (Poland). Poland Vital Registration - Causes of Death Data

307. Bulhoes C, Ramos E, Lindert J, Dias S, Barros H. Depressive symptoms and its associated factors in 13-year-old urban adolescents. *Int J Environ Res Public Health* 2013; **10**(10): 5026-38.

308. World Health Organization (WHO). Portugal World Health Survey 2003. Geneva, Switzerland: World Health Organization (WHO), 2005.

309. Antunes A, Frasquilho D, Azeredo-Lopes S, et al. Disability and common mental disorders: Results from the World Mental Health Survey Initiative Portugal. *Eur Psychiatry* 2018; **49**: 56-61.

310. Machado PP, Machado BC, Goncalves S, Hoek HW. The prevalence of eating disorders not otherwise specified. *Int J Eat Disord* 2007; **40**(3): 212-7.

311. doCarmo I, Reis D, Varandas P, et al. Prevalence of anorexia nervosa: A Portuguese population study. *Eur Eat Disord Rev* 1996; **4**(3): 157-70.

312. European Monitoring Centre for Drugs and Drug Addiction (EMCDDA). Portugal National Report to the EMCDDA 2002. Lisbon, Portugal: European Monitoring Centre for Drugs and Drug Addiction (EMCDDA), 2002.

313. World Health Organization (WHO). Portugal Vital Registration. Deaths ICD10 as it appears in WHO Mortality Database Geneva, Switzerland: World Health Organization (WHO).

314. Ministry of Health (Portugal). Portugal Hospital Inpatient Discharges 2015. 2015.

315. World Health Organization (WHO). Romania Vital Registration. Deaths ICD10 as it appears in WHO Mortality Database Geneva, Switzerland: World Health Organization (WHO).

316. World Health Organization (WHO). Slovakia World Health Survey 2003. Geneva, Switzerland: World Health Organization (WHO), 2005.

317. Okruhlica L, Mihalkova A, Klempova D, Skovayova L. Three-year follow-up study of heroin users in Bratislava. *Eur Addict Res* 2002; **8**(2): 103-6.

318. European Monitoring Centre for Drugs and Drug Addiction (EMCDDA). Slovakia National Report to the EMCDDA 2006, 2006.

319. Public Health Authority of the Slovak Republic, World Health Organization (WHO). Slovakia WHO Multi-country Survey Study on Health and Health System Responsiveness 2000. 2000.

320. Statistical Office of the Slovak Republic. Slovakia Drug Prevalence 2006 According to Gender and Age, 2006.

321. World Health Organization (WHO). Slovakia Vital Registration. Deaths ICD10 as it appears in WHO Mortality Database Geneva, Switzerland: World Health Organization (WHO).

322. World Health Organization (WHO). Slovenia World Health Survey 2003. Geneva, Switzerland: World Health Organization (WHO), 2005.

323. Nolimal D. Estimating the extent of the heroin problem in Slovenia: application of the key informant approach and the nomination technique where there are no other reliable sources of information. *Bull Narc* 1996; **48**(1-2): 121-34.

324. World Health Organization (WHO). Slovenia Vital Registration. Deaths ICD10 as it appears in WHO Mortality Database Geneva, Switzerland: World Health Organization (WHO).

325. Moreno B, Garcia-Alonso CR, Negrin Hernandez MA, Torres-Gonzalez F, Salvador-Carulla L. Spatial analysis to identify hotspots of prevalence of schizophrenia. *Soc Psychiatry Psychiatr Epidemiol* 2008; **43**(10): 782-91.

326. Moreno-Kustner B, Mayoral F, Navas-Campana D, et al. Prevalence of schizophrenia and related disorders in Malaga (Spain): results using multiple clinical databases. *Epidemiol Psychiatr Sci* 2016; **25**(1): 38-48.

327. Canals J, Marti-Henneberg C, Fernandez-Ballart J, Domenech E. A longitudinal study of depression in an urban Spanish pubertal population. *Eur Child Adolesc Psychiatry* 1995; **4**(2): 102-11.

328. Canals-Sans J, Hernandez-Martinez C, Saez-Carles M, Arija-Val V. Prevalence of DSM-5 depressive disorders and comorbidity in Spanish early adolescents: Has there been an increase in the last 20 years? *Psychiatry Res* 2018; **268**: 328-34.

329. Polaino-Lorente A, Domenech E. Prevalence of childhood depression: results of the first study in Spain. *J Child Psychol Psychiatry* 1993; **34**(6): 1007-17.

330. Canals J, Domenech E, Carbajo G, Blade J. Prevalence of DSM-III-R and ICD-10 psychiatric disorders in a Spanish population of 18-year-olds. *Acta Psychiatr Scand* 1997; **96**(4): 287-94.

331. Navarro-Mateu F, Tormo MJ, Salmeron D, et al. Prevalence of Mental Disorders in the South-East of Spain, One of the European Regions Most Affected by the Economic Crisis: The Cross-Sectional PEGASUS-Murcia Project. *PLoS One* 2015; **10**(9): e0137293.

332. Sendra-Gutierrez JM, Asensio-Moreno I, Vargas-Aragon ML. Characteristics and factors associated with depression in the elderly in Spain from a gender perspective. *Actas Esp Psiquiatr* 2017; **45**(5): 185-200.

333. World Health Organization (WHO). Spain World Health Survey 2002-2003. Geneva, Switzerland: World Health Organization (WHO), 2005.

334. De Dios C, Ezquiaga E, Agud JL, Vieta E, Soler B, Garcia-Lopez A. Subthreshold symptoms and time to relapse/recurrence in a community cohort of bipolar disorder outpatients. *J Affect Disord* 2012; **143**(1-3): 160-5.

335. Montes JM, Saiz J, de Dios C, et al. [Profile of bipolar disorder outpatients: a cross-sectional study in the Madrid Community]. *Actas Esp Psiquiatr* 2008; **36**(5): 277-84.

336. Canals J, Voltas N, Hernandez-Martinez C, Cosi S, Arija V. Prevalence of DSM-5 anxiety disorders, comorbidity, and persistence of symptoms in Spanish early adolescents. *Eur Child Adolesc Psychiatry* 2019; **28**(1): 131-43.

337. Cervilla JA, Gutierrez B, Rodriguez-Barranco M, et al. A Cross-Sectional Study on the Prevalence and Risk Correlates of Mental Disorders: The GRANADSigmaP Study. *J Nerv Ment Dis* 2018; **206**(9): 716-25.

338. Beato-Fernandez L, Rodriguez-Cano T, Belmonte-Llario A, Martinez-Delgado C. Risk factors for eating disorders in adolescents. A Spanish community-based longitudinal study. *Eur Child Adolesc Psychiatry* 2004; **13**(5): 287-94.

339. Pelaez Fernandez MA, Labrador FJ, Raich RM. Prevalence of eating disorders among adolescent and young adult scholastic population in the region of Madrid (Spain). *J Psychosom Res* 2007; **62**(6): 681-90.

340. Gual P, Perez-Gaspar M, Martinez-Gonzalez MA, Lahortiga F, de Irala-Estevez J, Cervera-Enguix S. Self-esteem, personality, and eating disorders: baseline assessment of a prospective population-based cohort. *Int J Eat Disord* 2002; **31**(3): 261-73.

341. Sancho C, Arija MV, Canals J. Personality in non-clinical adolescents with eating disorders. *Eur Eat Disord Rev* 2008; **16**(2): 133-8.

342. Lahortiga-Ramos F, De Irala-Estevez J, Cano-Prous A, Gual-Garcia P, Martinez-Gonzalez MA, Cervera-Enguix S. Incidence of eating disorders in Navarra (Spain). *Eur Psychiatry* 2005; **20**(2): 179-85.

343. University of Zaragoza (Spain). Spain Adolescent Eating Disorders Prevalence Survey 1999.

344. Andres MA, Catala MA, Gomez-Beneyto M. Prevalence, comorbidity, risk factors and service utilisation of disruptive behaviour disorders in a community sample of children in Valencia (Spain). *Soc Psychiatry Psychiatr Epidemiol* 1999; **34**(4): 175-9.

345. Benjumea Pino P, Mojarro Praxedes MD. Trastornos hipercineticos: estudio epidemiologico en doble fase de una poblacion sevillana. *Acta Psiquiatr Psicol Am Lat* 1993: 306–11.

346. Cardo E, Servera M, Vidal C, de Azua B, Redondo M, Riutort L. [The influence of different diagnostic criteria and the culture on the prevalence of attention deficit hyperactivity disorder]. *Rev Neurol* 2011; **52 Suppl 1**: S109-17.

347. Catala-Lopez F, Peiro S, Ridao M, Sanfelix-Gimeno G, Genova-Maleras R, Catala MA. Prevalence of attention deficit hyperactivity disorder among children and adolescents in Spain: a systematic review and meta-analysis of epidemiological studies. *BMC Psychiatry* 2012; **12**: 168.

348. Gomez-Beneyto M, Bonet A, Catala MA, Puche E, Vila V. Prevalence of mental disorders among children in Valencia, Spain. *Acta Psychiatr Scand* 1994; **89**(5): 352-7.

349. Lopez-Villalobos JA, Andres-De Llano J, Lopez-Sanchez MV, et al. Criterion validity and clinical usefulness of Attention Deficit Hyperactivity Disorder Rating Scale IV in attention deficit hyperactivity disorder (ADHD) as a function of method and age. *Psicothema* 2017; **29**(1): 103-10.

350. Rodriguez Molinero L, Lopez Villalobos J, Garrido Redondo M, Sacristan Martin A, Martinez Rivera M, Ruiz S. Estudio psicometrico-clinico de prevalencia y comorbilidad del trastorno por deficit de atencion con hiperactividad en Castilla y Leon (Espana). *Rev Pediatr Aten Primaria* 2009: 251-70.

351. Government Delegation for the National Plan on Drugs (Spain), Ministry of Health SSaES. Spain Household Survey on Alcohol and Drugs 2013. 2013.

352. Guitart AM, Espelt A, Castellano Y, et al. Impact of alcohol use disorder on mortality: are there age and gender differences? *Gaceta Sanitaria* 2011; **25**(5): 385-90.

353. Espada JP, Sussman S, Medina TBH, Alfonso JP. Relation between substance use and depression among Spanish adolescents. *Rev Int Psicol Ter Psicol* 2011; **11**(79-90).

354. Government Delegation for the National Plan on Drugs (Spain). Spain Survey on Drug Use in Secondary Schools 1994.

355. Government Delegation for the National Plan on Drugs (Spain). Spain Survey on Drug Use in Secondary Schools 1996.

356. Government Delegation for the National Plan on Drugs (Spain). Spain Survey on Drug Use in Secondary Schools 1998.

357. Government Delegation for the National Plan on Drugs (Spain). Spain Survey on Drug Use in Secondary Schools 2000.

358. Ministry of Health and Consumer Affairs (Spain). Spain Annual Report on the National Health System 2007. Madrid, Spain: Ministry of Health, Social Services and Equality (Spain), 2009.

359. Ministry of Health SSaES. Spain Annual Report on the National Health System 2013. Madrid, Spain: Ministry of Health, Social Services and Equality (Spain), 2014.

360. Pérez Madruga A, Avila Escribano JJ, Rodríguez Treceño M, Paniagua Paniagua C, Recio Pérez J. Análisis retrospectivo y de seguimiento de pacientes dependientes a opiáceos, atendidos durante los años 1985-1990 *Psiquis (Madr)* 1998; **19**: 17-22.

361. Benavides FG, Ruiz-Fores N, Delclos J, Domingo-Salvany A. [Consumption of alcohol and other drugs by the active population in Spain]. *Gac Sanit* 2013; **27**(3): 248-53.

362. de la Fuente L, Molist G, Espelt A, et al. Mortality risk factors and excess mortality in a cohort of cocaine users admitted to drug treatment in Spain. *J Subst Abuse Treat* 2014; **46**(2): 219-26.

363. Esteban J, Gimeno C, Barril J, Aragonés A, Climent JM, de la Cruz Pellín M. Survival study of opioid addicts in relation to its adherence to methadone maintenance treatment *Drug Alcohol Depend* 2003; **70**: 193-200.

364. European Monitoring Centre for Drugs and Drug Addiction (EMCDDA), Government Delegation for the National Plan on Drugs (Spain), Ministry of Health and Consumer Affairs (Spain). Spain National Report to the EMCDDA 2006, 2006.

365. European Monitoring Centre for Drugs and Drug Addiction (EMCDDA), Government Delegation for the National Plan on Drugs (Spain), Ministry of Health and Consumer Affairs (Spain). Spain National Report to the EMCDDA 2012, 2012.

366. European Monitoring Centre for Drugs and Drug Addiction (EMCDDA), Government Delegation for the National Plan on Drugs (Spain), Ministry of Health SSaES. Spain Household Survey on Alcohol and Drugs 2015-2016.

367. Ferri López A, Martínez-Martínez MI, Martínez-Raga J, López-Seguí MP, Didia Attas J. [Study on drug use among students in the province of Valencia, Spain] *Vertex* 2013; **24**: 333–41.

368. Font-Mayolas S, Gras ME, Cebrian N, Salamo A, Planes M, Sullman MJM. Types of polydrug use among Spanish adolescents. *Addictive Behaviors* 2013; **38**(3): 1605-9.

369. Government Delegation for the National Plan on Drugs (Spain), Ministry of Health SSaES. Spain Survey on Drug Use in Secondary Schools 2002.

370. Government Delegation for the National Plan on Drugs (Spain), Ministry of Health SSaES. Spain Survey on Drug Use in Secondary Schools 2004 Madrid, Spain: Ministry of Health, Social Services and Equality (Spain).

371. Government Delegation for the National Plan on Drugs (Spain), Ministry of Health SSaES. Spain Survey on Drug Use in Secondary Schools 2006-2007 Madrid, Spain: Ministry of Health, Social Services and Equality (Spain).

372. Government Delegation for the National Plan on Drugs (Spain), Ministry of Health SSaES. Spain Survey on Drug Use in Secondary Schools 2008-2009 Madrid, Spain Ministry of Health, Social Services and Equality (Spain).

373. Government Delegation for the National Plan on Drugs (Spain), Ministry of Health SSaES. Spain Survey on Drug Use in Secondary Schools 2010-2011 Madrid, Spain Ministry of Health, Social Services and Equality (Spain).

374. Government Delegation for the National Plan on Drugs (Spain), Ministry of Health SSaES. Spain Survey on Drug Use in Secondary Schools 2012-2013 Madrid, Spain Ministry of Health, Social Services and Equality (Spain).

375. Government Delegation for the National Plan on Drugs (Spain), Ministry of Health SSaES. Spain Household Survey on Alcohol and Drugs 2007-2008. Madrid, Spain Ministry of Health, Social Services and Equality (Spain); 2007-2008.

376. Orti RM, Domingo-Salvany A, Munoz A, Macfarlane D, Suelves JM, Anto JM. Mortality trends in a cohort of opiate addicts, Catalonia, Spain. *Int J Epidemiol* 1996; **25**(3): 545-53.

377. Sanchez-Carbonell X, Seus L. Ten-year survival analysis of a cohort of heroin addicts in Catalonia: the EMETYST project. *Addiction* 2000; **95**(6): 941-8.

378. Secades-Villa R, Lopez-Nunez C, Fernandez-Artamendi S, Weidberg S, Fernandez-Hermida JR. Gender differences in the prevalence of DSM-IV alcohol use disorders in adolescents. *Adicciones* 2013; **25**(3): 260-8.

379. Muga R, Langohr K, Tor J, et al. Survival of HIV-infected injection drug users (IDUs) in the highly active antiretroviral therapy era, relative to sex- and age-specific survival of HIV-uninfected IDUs. *Clin Infect Dis* 2007; **45**(3): 370-6.

380. Government Delegation for the National Plan on Drugs (Spain), Ministry of Health SSaES. Spain Household Survey on Alcohol and Drugs 2007-2008. Madrid, Spain: Ministry of Health, Social Services and Equality (Spain).

381. World Health Organization (WHO). Spain Vital Registration. Deaths ICD10 as it appears in WHO Mortality Database Geneva, Switzerland: World Health Organization (WHO).

382. Jorgensen L, Ahlbom A, Allebeck P, Dalman C. The Stockholm non-affective psychoses study (snaps): the importance of including out-patient data in incidence studies. *Acta Psychiatr Scand* 2010; **121**(5): 389-92.

383. Osby U, Correia N, Brandt L, Ekbom A, Sparen P. Mortality and causes of death in schizophrenia in Stockholm county, Sweden. *Schizophr Res* 2000; **45**(1-2): 21-8.

384. Gale CR, Batty GD, Osborn DP, Tynelius P, Whitley E, Rasmussen F. Association of mental disorders in early adulthood and later psychiatric hospital admissions and mortality in a cohort study of more than 1 million men. *Arch Gen Psychiatry* 2012; **69**(8): 823-31.

385. Lindstrom E, Widerlov B, vonKnorring L. The ICD-10 and DSM-IV diagnostic criteria and the prevalence of schizophrenia. *Eur Psychiat* 1997; **12**(5): 217-23.

386. Fors BM, Isacson D, Bingefors K, Widerlov B. Mortality among persons with schizophrenia in Sweden: an epidemiological study. *Nord J Psychiatry* 2007; **61**(4): 252-9.

387. Widerlov B, Lindstrom E, vonKnorring L. One-year prevalence of long-term functional psychosis in three different areas of Uppsala. *Acta Psychiat Scand* 1997; **96**(6): 452-8.

388. Olsson GI, von Knorring AL. Adolescent depression: prevalence in Swedish high-school students. *Acta Psychiatr Scand* 1999; **99**(5): 324-31.

389. Johansson R, Carlbring P, Heedman A, Paxling B, Andersson G. Depression, anxiety and their comorbidity in the Swedish general population: point prevalence and the effect on health-related quality of life. *PeerJ* 2013; **1**: e98.

390. World Health Organization (WHO). Sweden World Health Survey 2003. Geneva, Switzerland: World Health Organization (WHO), 2005.

391. Karolinska Institute (Sweden), National Institute for Working Life (Sweden). Sweden - Stockholm County PART Study, Phase I 1998-2000.

392. Crump C, Sundquist K, Winkleby MA, Sundquist J. Comorbidities and mortality in bipolar disorder: a Swedish national cohort study. *JAMA Psychiatry* 2013; **70**(9): 931-9.

393. Westman J, Hallgren J, Wahlbeck K, Erlinge D, Alfredsson L, Osby U. Cardiovascular mortality in bipolar disorder: a population-based cohort study in Sweden. *BMJ Open* 2013; **3**(4).

394. Wentz E, Gillberg IC, Anckarsater H, Gillberg C, Rastam M. Adolescent-onset anorexia nervosa: 18-year outcome. *Br J Psychiatry* 2009; **194**(2): 168-74.

395. Norring CE, Sohlberg SS. Outcome, recovery, relapse and mortality across six years in patients with clinical eating disorders. *Acta Psychiatr Scand* 1993; **87**(6): 437-44.

396. Papadopoulos FC, Ekbom A, Brandt L, Ekselius L. Excess mortality, causes of death and prognostic factors in anorexia nervosa. *Br J Psychiatry* 2009; **194**(1): 10-7.

397. Nilsson K, Hagglof B. Long-term follow-up of adolescent onset anorexia nervosa in northern Sweden. *Eur Eat Disord Rev* 2005; **13**(2): 89-100.

398. Cullberg J, Engstrøm-Lindberg M. Prevalence and incidence of eating disorders in a suburban area. *Acta Psychiatr Scand* 1988; **78**: 314-10.

399. Rosling AM, Sparen P, Norring C, von Knorring AL. Mortality of Eating Disorders: A Follow-Up Study of Treatment in a Specialist Unit 1974-2000. *Int J Eat Disorder* 2011; **44**(4): 304-10.

400. Hultman CM, Sandin S, Levine SZ, Lichtenstein P, Reichenberg A. Advancing paternal age and risk of autism: new evidence from a population-based study and a meta-analysis of epidemiological studies. *Mol Psychiatry* 2011; **16**(12): 1203-12.

401. Idring S, Lundberg M, Sturm H, et al. Changes in prevalence of autism spectrum disorders in 2001-2011: findings from the Stockholm youth cohort. *J Autism Dev Disord* 2015; **45**(6): 1766-73.

402. Idring S, Rai D, Dal H, et al. Autism spectrum disorders in the Stockholm Youth Cohort: design, prevalence and validity. *PLoS One* 2012; **7**(7): e41280.

403. Hirvikoski T, Mittendorfer-Rutz E, Boman M, Larsson H, Lichtenstein P, Bolte S. Premature mortality in autism spectrum disorder. *Br J Psychiatry* 2016; **208**(3): 232-8.

404. Gillberg C, Cederlund M, Lamberg K, Zeijlon L. Brief report: "the autism epidemic". The registered prevalence of autism in a Swedish urban area. *J Autism Dev Disord* 2006; **36**(3): 429-35.

405. Holmberg K, Sundelin C, Hjern A. Screening for attention-deficit/hyperactivity disorder (ADHD): can high-risk children be identified in first grade? *Child Care Health Dev* 2013; **39**(2): 268-76.

406. Rasmussen P, Gillberg C. Natural outcome of ADHD with developmental coordination disorder at age 22 years: a controlled, longitudinal, community-based study. *J Am Acad Child Adolesc Psychiatry* 2000; **39**(11): 1424-31.

407. Gustavson KH, Umb-Carlsson O, Sonnander K. A follow-up study of mortality, health conditions and associated disabilities of people with intellectual disabilities in a Swedish county. *J Intell Disabil Res* 2005; **49**: 905-14.

408. Andreasson S, Allebeck P, Romelsjo A. Alcohol and Mortality among Young Men - Longitudinal-Study of Swedish Conscripts. *Brit Med J* 1988; **296**(6628): 1021-5.

409. Berge J, Hakansson A, Berglund M. Alcohol and drug use in groups of cannabis users: Results from a survey on drug use in the Swedish general population. *Am J Addiction* 2014; **23**(3): 272-9.

410. Berglund M, Tunving K. Assaultive alcoholics 20 years later. *Acta Psychiatr Scand* 1985; **71**(2): 141-7.

411. Dahlgren L, Myrhed M. Alcoholic females. II. Causes of death with reference to sex difference. *Acta Psychiatr Scand* 1977; **56**(2): 81-91.

412. Hvitfeldt T, Romelsjö A, Damström TK, Karolinska Institute. Alkohol och droger: vanor och problem i Stockholms län 1996 [Alcohol and Drug Use: Patterns and Problems in Stockholm County in 1996]. Stockholm, Sweden:: Karolinksa Institute, 1999.

413. Kallmen H, Wennberg P, Berman AH, Bergman H. Alcohol habits in Sweden during 1997-2005 measured with the AUDIT. *Nord J Psychiat* 2007; **61**(6): 466-70.

414. Kallmen H, Wennberg P, Ramstedt M, Hallgren M. Changes in alcohol consumption between 2009 and 2014 assessed with the AUDIT. *Scand J Public Healt* 2015; **43**(4): 381-4.

415. Lindberg S, Agren G. Mortality among Male and Female Hospitalized Alcoholics in Stockholm 1962-1983. *Brit J Addict* 1988; **83**(10): 1193-200.

416. Lundin A, Hallgren M, Balliu N, Forsell Y. The use of alcohol use disorders identification test (AUDIT) in detecting alcohol use disorder and risk drinking in the general population: validation of AUDIT using schedules for clinical assessment in neuropsychiatry *Alcohol Clin Exp Res* 2015; **39**: 158-65.

417. Lundin A, Hallgren M, Forsman M, Forsell Y. Comparison of DSM-5 Classifications of Alcohol Use Disorders With Those of DSM-IV, DSM-III-R, and ICD-10 in a General Population Sample in Sweden. *J Stud Alcohol Drugs* 2015; **76**: 773-80.

418. Rehm J, Room R, van den Brink W, Jacobi F. Alcohol use disorders in EU countries and Norway: an overview of the epidemiology. *Eur Neuropsychopharmacol* 2005; **15**(4): 377-88.

419. Ahacic K, Kareholt I, Helgason AR, Allebeck P. Non-response bias and hazardous alcohol use in relation to previous alcohol-related hospitalization: comparing survey responses with population data. *Subst Abuse Treat Pr* 2013; **8**.

420. Medhus A. Mortality among female alcoholics *Scand J Soc Med* 1975; **3**(3): 111-5.

421. Spak F, Hällström T. Prevalence of female alcohol dependence and abuse in Sweden. *Addiction* 1995; **90**(8): 1077-88.

422. Durbeej N, Berman AH, Gumpert CH, Palmstierna T, Kristiansson M, Alm C. Validation of the Alcohol Use Disorders Identification Test and the Drug Use Disorders Identification Test in a Swedish sample of suspected offenders with signs of mental health problems: results from the Mental Disorder, Substance Abuse and Crime study. *J Subst Abuse Treat* 2010; **39**(4): 364-77.

423. Fridell M, Hesse M. Psychiatric severity and mortality in substance abusers: a 15-year follow-up of drug users. *Addict Behav* 2006; **31**(4): 559-65.

424. Fugelstad A, Annell A, Rajs J, Agren G. Mortality and causes and manner of death among drug addicts in Stockholm during the period 1981-1992. *Acta Psychiatr Scand* 1997; **96**(3): 169-75.

425. Lindstrom M, Rosvall M. Daily tobacco smoking, heavy alcohol use, and hashish use among adolescents in southern Sweden: A population-based multilevel study. *Addict Behav Rep* 2015; **2**: 6-12.

426. Lund University (Sweden), Statistics Sweden, Swedish National Institute of Public Health. Sweden Survey on Drug Use 2008-2009.

427. Stenbacka M, Leifman A, Romelsjo A. Mortality Among Opiate Abusers in Stockholm: A Longitudinal Study. *Heroin Addict Relat Clin Probl* 2007; **9**(3): 41-50.

428. Swedish Council for Information on Alcohol and Other Drugs (CAN). Sweden Heavy Drug Use 1998. Stockholm, Sweden: Swedish Council for Information on Alcohol and Other Drugs (CAN), 2001.

429. Wahren CA, Brandt L, Allebeck P. Has mortality in drug addicts increased? A comparison between two hospitalized cohorts in Stockholm. *Int J Epidemiol* 1997; **26**(6): 1219-26.

430. National Board of Health and Welfare (Sweden). Sweden - Stockholm County Vital Registration

431. National Board of Health and Welfare (Sweden). Sweden Cause of Death Register.

432. Michel C, Schnyder N, Schmidt SJ, Groth N, Schimmelmann BG, Schultze-Lutter F. Functioning mediates help-seeking for mental problems in the general population. *Eur Psychiatry* 2018; **54**: 1-9.

433. Richard A, Rohrmann S, Vandeleur CL, Schmid M, Barth J, Eichholzer M. Loneliness is adversely associated with physical and mental health and lifestyle factors: Results from a Swiss national survey. *PLoS One* 2017; **12**(7): e0181442.

434. Steinhausen HC, Winkler C, Meier M. Eating disorders in adolescence in a Swiss epidemiological study. *Int J Eat Disord* 1997; **22**(2): 147-51.

435. Steinhausen HC, Metzke CW, Meier M, Kannenberg R. Prevalence of child and adolescent psychiatric disorders: the Zurich Epidemiological Study. *Acta Psychiatr Scand* 1998; **98**(4): 262-71.

436. Gache P, Michaud P, Landry U, et al. The Alcohol Use Disorders Identification Test (AUDIT) as a screening tool for excessive drinking in primary care: reliability and validity of a French version. *Alcohol Clin Exp Res* 2005; **29**(11): 2001-7.

437. Mohler-Kuo M, Foster S, Gmel G, Dey M, Dermota P. DSM-IV and DSM-5 alcohol use disorder among young Swiss men *Addiction* 2015; **110**(3): 429-40.

438. Baggio S, N’Goran AA, Deline S, et al. Patterns of cannabis use and prospective associations with health issues among young males *Addiction* 2014; **109**(6): 937-45.

439. World Health Organization (WHO). Switzerland Vital Registration. Deaths ICD10 as it appears in WHO Mortality Database Geneva, Switzerland: World Health Organization (WHO).

440. McCreadie RG, Leese M, Tilak-Singh D, Loftus L, MacEwan T, Thornicroft G. Nithsdale, Nunhead and Norwood: similarities and differences in prevalence of schizophrenia and utilisation of services in rural and urban areas. *Br J Psychiatry* 1997; **170**: 31-6.

441. Bamrah JS, Freeman HL, Goldberg DP. Epidemiology of schizophrenia in Salford, 1974-84. Changes in an urban community over ten years. *Br J Psychiatry* 1991; **159**: 802-10.

442. Bhavsar V, Boydell J, Murray R, Power P. Identifying aspects of neighbourhood deprivation associated with increased incidence of schizophrenia. *Schizophr Res* 2014; **156**(1): 115-21.

443. Brewin J, Cantwell R, Dalkin T, et al. Incidence of schizophrenia in Nottingham. A comparison of two cohorts, 1978-80 and 1992-94. *Br J Psychiatry* 1997; **171**: 140-4.

444. Brown S, Kim M, Mitchell C, Inskip H. Twenty-five year mortality of a community cohort with schizophrenia. *Br J Psychiatry* 2010; **196**(2): 116-21.

445. Harvey CA, Pantelis C, Taylor J, et al. The Camden schizophrenia surveys. II. High prevalence of schizophrenia in an inner London borough and its relationship to socio-demographic factors. *Br J Psychiatry* 1996; **168**(4): 418-26.

446. Hassall C, Prior P, Cross KW. A preliminary study of excess mortality using a psychiatric case register. *J Epidemiol Community Health* 1988; **42**(3): 286-9.

447. Healy D, Le Noury J, Harris M, et al. Mortality in schizophrenia and related psychoses: data from two cohorts, 1875-1924 and 1994-2010. *BMJ Open* 2012; **2**(5).

448. Herrman HE, Baldwin JA, Christie D. A record-linkage study of mortality and general hospital discharge in patients diagnosed as schizophrenic. *Psychol Med* 1983; **13**(3): 581-93.

449. Shivashankar S, Telfer S, Arunagiriraj J, et al. Has the prevalence, clinical presentation and social functioning of schizophrenia changed over the last 25 years? Nithsdale schizophrenia survey revisited. *Schizophr Res* 2013; **146**(1-3): 349-56.

450. McNaught AS, Jeffreys SE, Harvey CA, Quayle AS, King MB, Bird AS. The Hampstead Schizophrenia Survey 1991. II: Incidence and migration in inner London. *Br J Psychiatry* 1997; **170**: 307-11.

451. Donnelly M. Depression among adolescents in Northern Ireland. *Adolescence* 1995; **30**(118): 339-50.

452. Meltzer H, Gatward R, Goodman R, Ford T. Mental health of children and adolescents in Great Britain. *Int Rev Psychiatry* 2003; **15**(1-2): 185-7.

453. Office for National Statistics. Social and Vital Statistics Division et al. Mental Health of Children and Young People in Great Britain, 2004 [computer file]. Colchester, Essex:: UK Data Archive.

454. Jenkins R, Lewis G, Bebbington P, et al. The National Psychiatric Morbidity surveys of Great Britain--initial findings from the household survey. *Psychol Med* 1997; **27**(4): 775-89.

455. McConnell P, Bebbington P, McClelland R, Gillespie K, Houghton S. Prevalence of psychiatric disorder and the need for psychiatric care in Northern Ireland. Population study in the District of Derry. *Br J Psychiatry* 2002; **181**: 214-9.

456. National Centre for Social Research, Leicester Uo. Adult Psychiatric Morbidity Survey 2007. Colcester: National Centre for Social Research (NatCen), University of Leicester, 2011.

457. Office for National Statistics. Psychiatric Morbidity among Adults Living in Private Households, 2000 [computer file]. Colchester, Essex: UK Data Archive.

458. Office of Population Censuses and Surveys., Social Survey Division. OPCS Surveys of Psychiatric Morbidity : Private Household Survey, 1993. Colchester, Essex: UK Data Archive; 1996.

459. Public Health England. United Kingdom - Great Britain Psychiatric Morbidity Survey Custom Tabulations 1993-2007.

460. West P, Sweeting H, Der G, Barton J, Lucas C. Voice-DISC identified DSM-IV disorders among 15-year-olds in the west of Scotland. *J Am Acad Child Adolesc Psychiatry* 2003; **42**(8): 941-9.

461. World Health Organization (WHO). United Kingdom World Health Survey 2004. Geneva, Switzerland: World Health Organization (WHO), 2005.

462. Ajetunmobi O, Taylor M, Stockton D, Wood R. Early death in those previously hospitalised for mental healthcare in Scotland: a nationwide cohort study, 1986-2010. *BMJ Open* 2013; **3**(7).

463. Chang CK, Hayes RD, Broadbent M, et al. All-cause mortality among people with serious mental illness (SMI), substance use disorders, and depressive disorders in southeast London: a cohort study. *BMC Psychiatry* 2010; **10**: 77.

464. Scott J, Grunze H, Meyer TD, Nendick J, Watkins H, Ferrier N. A bipolar II cohort (ABC): The association of functional disability with gender and rapid cycling. *J Affect Disord* 2015; **185**: 204-8.

465. Cruz N, Vieta E, Comes M, et al. Rapid-cycling bipolar I disorder: course and treatment outcome of a large sample across Europe. *J Psychiatr Res* 2008; **42**(13): 1068-75.

466. Ford T, Goodman R, Meltzer H. The British Child and Adolescent Mental Health Survey 1999: the prevalence of DSM-IV disorders. *J Am Acad Child Adolesc Psychiatry* 2003; **42**(10): 1203-11.

467. Crisp A. Death, survival and recovery in anorexia nervosa: A thirty five year study. *Eur Eat Disord Rev* 2006; **14**(3): 168-75.

468. Crisp AH, Callender JS, Halek C, Hsu LK. Long-term mortality in anorexia nervosa. A 20-year follow-up of the St George's and Aberdeen cohorts. *Br J Psychiatry* 1992; **161**: 104-7.

469. Hoang U, Goldacre M, James A. Mortality following hospital discharge with a diagnosis of eating disorder: national record linkage study, England, 2001-2009. *Int J Eat Disord* 2014; **47**(5): 507-15.

470. Millar HR, Wardell F, Vyvyan JP, Naji SA, Prescott GJ, Eagles JM. Anorexia nervosa mortality in Northeast Scotland, 1965-1999. *Am J Psychiatry* 2005; **162**(4): 753-7.

471. Button EJ, Chadalavada B, Palmer RL. Mortality and predictors of death in a cohort of patients presenting to an eating disorders service. *Int J Eat Disord* 2010; **43**(5): 387-92.

472. Fairburn CG, Cooper Z, Doll HA, Norman P, O'Connor M. The natural course of bulimia nervosa and binge eating disorder in young women. *Arch Gen Psychiatry* 2000; **57**(7): 659-65.

473. Fombonne E, Simmons H, Ford T, Meltzer H, Goodman R. Prevalence of pervasive developmental disorders in the British nationwide survey of child mental health. *J Am Acad Child Adolesc Psychiatry* 2001; **40**(7): 820-7.

474. Harrison MJ, O'Hare AE, Campbell H, Adamson A, McNeillage J. Prevalence of autistic spectrum disorders in Lothian, Scotland: an estimate using the "capture-recapture" technique. *Arch Dis Child* 2006; **91**(1): 16-9.

475. Latif AH, Williams WR. Diagnostic trends in autistic spectrum disorders in the South Wales valleys. *Autism* 2007; **11**(6): 479-87.

476. Scott FJ, Baron-Cohen S, Bolton P, Brayne C. Brief report: prevalence of autism spectrum conditions in children aged 5-11 years in Cambridgeshire, UK. *Autism* 2002; **6**(3): 231-7.

477. Totsika V, Hastings RP, Emerson E, Lancaster GA, Berridge DM. A population-based investigation of behavioural and emotional problems and maternal mental health: associations with autism spectrum disorder and intellectual disability. *J Child Psychol Psychiatry* 2011; **52**(1): 91-9.

478. Webb E, Morey J, Thompsen W, Butler C, Barber M, Fraser WI. Prevalence of autistic spectrum disorder in children attending mainstream schools in a Welsh education authority. *Dev Med Child Neurol* 2003; **45**(6): 377-84.

479. Williams E, Thomas K, Sidebotham H, Emond A. Prevalence and characteristics of autistic spectrum disorders in the ALSPAC cohort. *Dev Med Child Neurol* 2008; **50**(9): 672-7.

480. Brugha TS, McManus S, Bankart J, et al. Epidemiology of autism spectrum disorders in adults in the community in England. *Arch Gen Psychiatry* 2011; **68**(5): 459-65.

481. National Centre for Social Research (NatCen). England Adult Psychiatric Morbidity Survey 2014-2015: National Centre for Social Research (NatCen), University of Leicester.

482. Agnew-Blais JC, Polanczyk GV, Danese A, Wertz J, Moffitt TE, Arseneault L. Evaluation of the Persistence, Remission, and Emergence of Attention-Deficit/Hyperactivity Disorder in Young Adulthood. *JAMA Psychiatry* 2016; **73**(7): 713-20.

483. Baird G, Simonoff E, Pickles A, et al. Prevalence of disorders of the autism spectrum in a population cohort of children in South Thames: the Special Needs and Autism Project (SNAP). *Lancet* 2006; **368**(9531): 210-5.

484. Goodman R, Ford T, Richards H, Gatward R, Meltzer H. The Development and Well-Being Assessment: description and initial validation of an integrated assessment of child and adolescent psychopathology. *J Child Psychol Psychiatry* 2000; **41**(5): 645-55.

485. McElroy E, Shevlin M, Murphy J, McBride O. Co-occurring internalizing and externalizing psychopathology in childhood and adolescence: a network approach. *Eur Child Adolesc Psychiatry* 2018; **27**(11): 1449-57.

486. Marshall EJ, Edwards G, Taylor C. Mortality in men with drinking problems: a 20-year follow-up. *Addiction* 1994; **89**(10): 1293-8.

487. National Centre for Social Research (NatCen). England Adult Psychiatric Morbidity Survey 2006-2007: National Centre for Social Research (NatCen), University of Leicester.

488. Nicholls P, Edwards G, Kyle E. Alcoholics admitted to four hospitals in England. II. General and cause-specific mortality. *Q J Stud Alcohol* 1974; **35**(3): 841-55.

489. Coulton S, Drummond C, James D, et al. Opportunistic screening for alcohol use disorders in primary care: comparative study. *Bmj-Brit Med J* 2006; **332**(7540): 511-4a.

490. British Market Research Bureau (BMRB), Social Research. Scottish Schools Adolescent Lifestyle and Substance Use Survey, 2006 [computer file]. . Colchester, Essex UK Data Archive [distributor].

491. Health and Social Care Information Centre (HSCIC), NHS, National Centre for Social Research (NatCen), National Foundation for Educational Research (NFER). England Smoking, Drinking and Drug Use Among Young People Survey 2014 - HSCIC.

492. National Centre for Social Research, National Foundation for Educational Research. Smoking, Drinking and Drug Use among Young People Colchester, Essex: UK Data Archive 2000.

493. National Centre for Social Research (NatCen), National Foundation for Educational Research (NFER) (UK). United Kingdom - Smoking, Drinking and Drug Use Among Young People in England in 2006: HSCIC.

494. National Centre for Social Research (NatCen), National Foundation for Educational Research (NFER) (UK). Scotland - Smoking, Drinking and Drug Use Among Young People in England in 2000: Scottish Government.

495. NHS National Services Scotland, Information Services Division. Scottish Schools Adolescent Lifestyle and Substance Use Survey. Colchester, Essex: UK Data Archive; 2008.

496. NHS National Services Scotland, Information Services Division. Scottish Schools Adolescent Lifestyle and Substance Use Survey. Colchester, Essex: UK Data Archive; 2010.

497. NHS National Services Scotland, Information Services Division. Scottish Schools Adolescent Lifestyle and Substance Use Survey. Colchester, Essex: UK Data Archive; 2015.

498. Currie C, Corbett J. Scottish Schools Adolescent Lifestyle and Substance Use Survey, 2002. Colchester, Essex: UK Data Archive

499. Currie C, Corbett J. Scottish Schools Adolescent Lifestyle and Substance Use Survey, 2004. Colchester, Essex: UK Data Archive

500. De Angelis D, Hickman M, Yang SY. Estimating long-term trends in the incidence and prevalence of opiate use/injecting drug use and the number of former users: Back-calculation methods and opiate overdose deaths. *American Journal of Epidemiology* 2004; **160**(10): 994-1004.

501. Frischer M, Hickman M, Kraus L, Mariani F, Wiessing L. A comparison of different methods for estimating the prevalence of problematic drug misuse in Great Britain. *Addiction* 2001; **96**(10): 1465-76.

502. Hatch SL, Woodhead C, Frissa S, et al. Importance of thinking locally for mental health: data from cross-sectional surveys representing South East London and England. *PLoS One* 2012; **7**(12): e48012.

503. Singleton N, Bumpstead R, O'Brien M, Lee A, Meltzer H. Psychiatric morbidity among adults living in private households, 2000. *Int Rev Psychiatry* 2003; **15**(1): 65-73.

504. TNS-BMRB Scotland, Scottish Government. Scottish Crime and Justice Survey, 2009-2010 Colchester, Essex: UK Data Archive

505. United States Department of Health and Human Services, Substance Abuse and Mental Health Services Administration, Office of Applied Studies. National Survey on Drug Use and Health, 2006. ICPSR21240-v5. Ann Arbor, MI: Inter-university Consortium for Political and Social Research.

506. United States Department of Health and Human Services, Substance Abuse and Mental Health Services Administration, Office of Applied Studies. National Survey on Drug Use and Health, 2007. ICPSR23782-v3. Ann Arbor, MI: Inter-university Consortium for Political and Social Research.

507. United States Department of Health and Human Services, Substance Abuse and Mental Health Services Administration, Office of Applied Studies. National Survey on Drug Use and Health, 2009. ICPSR29621-v3. Ann Arbor, MI: Inter-university Consortium for Political and Social Research.

508. Seaman SR, Brettle RP, Gore SM. Mortality from overdose among injecting drug users recently released from prison: database linkage study. *BMJ* 1998; **316**(7129): 426-8.

509. British Market Research Bureau (BMRB), Scottish Government. United Kingdom - Scottish Crime and Victimisation Survey 2006: Scottish Government.

510. Department of Justice (Northern Ireland), Northern Ireland Statistics and Research Agency (NISRA). United Kingdom - Northern Ireland Crime Survey 2006-2007.

511. Ghodse H, Oyefeso A, Kilpatrick B. Mortality of drug addicts in the United Kingdom 1967-1993. *Int J Epidemiol* 1998; **27**(3): 473-8.

512. Home Office, Crime and Criminal Justice Unit, Office of Population Censuses and Surveys, Social Survey Division. British Crime Survey, 1994. [data collection]: UK Data Service, 2000.

513. Home Office (United Kingdom). United Kingdom - British Crime Survey 2006-2007.

514. Johnson A, London School of Hygiene and Tropical Medicine, Centre for Sexual and Reproductive Health Research, NatCen Social Research. National Survey of Sexual Attitudes and Lifestyles, 2010-2012 [computer file]. Colchester, Essex: UK Data Archive.

515. Northern Ireland Office, Northern Ireland Statistics and Research Agency (NISRA). Northern Ireland Crime Survey 2001. United Kingdom

516. Northern Ireland Office, Northern Ireland Statistics and Research Agency (NISRA). Northern Ireland Crime Survey 2003-2004. United Kingdom

517. Northern Ireland Office, Northern Ireland Statistics and Research Agency (NISRA). Northern Ireland Crime Survey 2005. United Kingdom

518. Oppenheimer E, Tobutt C, Taylor C, Andrew T. Death and survival in a cohort of heroin addicts from London clinics: a 22-year follow-up study. *Addiction* 1994; **89**(10): 1299-308.

519. Rathod NH, Addenbrooke WM, Rosenbach AF. Heroin dependence in an English town: 33-year follow-up. *Br J Psychiatry* 2005; **187**: 421-5.

520. Scottish Government. United Kingdom - Scottish Crime and Victimisation Survey 2004. Scottish Government.

521. TNS-BMRB Scotland, Scottish Government. Scottish Crime and Justice Survey, 2008-2009 Colchester, Essex: UK Data Service 2010.

522. Williams L, H P. Alcohol, cannabis, ecstasy and cocaine: Drugs of reasoned choice amongst young adult recreational drug users in England. *Int J Drug Policy* 1999; **12**(5-6): 397-413.

523. European School Survey Project on Alcohol and Other Drugs (ESPAD), Pompidou Group CoE, Swedish Council for Information on Alcohol and Other Drugs (CAN). ESPAD Report 1995: Alcohol and Other Drug Use Among Students in 26 European Countries. Stockholm, Sweden: Swedish Council for Information on Alcohol and Other Drugs (CAN), 1997.

524. National Centre for Social Research, National Foundation for Educational Research. Smoking, Drinking and Drug Use among Young People Colchester, Essex: UK Data Archive 2012.

525. Office for National Statistics (ONS) (United Kingdom). United Kingdom - England and Wales Mortality Data.

526. Office for National Statistics (ONS) (United Kingdom). United Kingdom - England Mortality Statistics.

527. World Health Organization (WHO). Northern Ireland Vital Registration. Deaths ICD10 as it appears in WHO Mortality Database Geneva, Switzerland: World Health Organization (WHO).

528. World Health Organization (WHO). Scotland Vital Registration. Deaths ICD10 as it appears in WHO Mortality Database Geneva, Switzerland: World Health Organization (WHO).

## Authors’ Contributions

Contributions to be included in appendix at the above location:

**Providing data or critical feedback on data sources**

Emilie E Agardh, Peter Allebeck, Benedetta Armocida, Maciej Banach, Till Winfried Bärnighausen, Akshaya Srikanth Bhagavathula, Giulio Castelpietra, Vijay Kumar Chattu, Diana Dias da Silva, Adeniyi Francis Fagbamigbe, Alize J Ferrari, Peter Andras Gaal, Juanita A Haagsma, Josep Maria Haro, Kim Moesgaard Iburg, Jost B Jonas, Tamas Joo, Jacek Jerzy Jozwiak, Srinivasa Vittal Katikireddi, Moien AB Khan, Adnan Kisa, Sezer Kisa, Ann Kristin Skrindo Knudsen, Giancarlo Logroscino, Bartosz Miazgowski, Babak Moazen, Shafiu Mohammed, Gabriele Nagel, Mohsen Naghavi, Ionut Negoi, Ruxandra Irina Negoi, Songhomitra Panda-Jonas, Shahina Pardhan, Jay Patel, Maarten J Postma, Luca Ronfani, Farhad Shokraneh, João Pedro Silva, Fabrizio Starace, Miklós Szócska, Rafael Tabarés-Seisdedos, Marcos Roberto Tovani-Palone, and Tommi Juhani Vasankari.

**Development of methods or computational machinery**

Akshaya Srikanth Bhagavathula, Giulio Castelpietra, Alize J Ferrari, Adnan Kisa, Mohsen Naghavi, and Shahina Pardhan.

**Providing critical feedback on methods or results**

Emilie E Agardh, Peter Allebeck, Catalina Liliana Andrei, Tudorel Andrei, Benedetta Armocida, Jose L Ayuso-Mateos, Maciej Banach, Till Winfried Bärnighausen, Akshaya Srikanth Bhagavathula, Giulio Castelpietra, Joht Singh Chandan, Vijay Kumar Chattu, Natália Cruz-Martins, Paul I Dargan, Keshab Deuba, Diana Dias da Silva, Adeniyi Francis Fagbamigbe, Alize J Ferrari, Florian Fischer, Peter Andras Gaal, Juanita A Haagsma, Josep Maria Haro, M Tasdik Hasan, Syed Shahzad Hasan, Simon I Hay, Kim Moesgaard Iburg, Elham Jamshidi, Jost B Jonas, Tamas Joo, Jacek Jerzy Jozwiak, Srinivasa Vittal Katikireddi, Joonas H Kauppila, Moien AB Khan, Adnan Kisa, Sezer Kisa, Mika Kivimäki, Ann Kristin Skrindo Knudsen, Kamrun Nahar Koly, Ai Koyanagi, Manasi Kumar, Tea Lallukka, Caterina Ledda, Ilaria Lega, Giancarlo Logroscino, Joana A Loureiro, Jose Martinez-Raga, Manu Raj Mathur, Alexios-Fotios A Mentis, Tomislav Mestrovic, Andreea Mirica, Babak Moazen, Shafiu Mohammed, Lorenzo Monasta, Francesk Mulita, Gabriele Nagel, Mohsen Naghavi, Ionut Negoi, Ruxandra Irina Negoi, Vincent Ebuka Nwatah, Alicia Padron-Monedero, Songhomitra Panda-Jonas, Shahina Pardhan, Maja Pasovic, Jay Patel, Ionela-Roxana Petcu, Richard Charles G Pollok, Maarten J Postma, David Laith Rawaf, Salman Rawaf, Esperanza Romero-Rodríguez, Francesco Sanmarchi, Michael P Schaub, Nigussie Tadesse Sharew, Rahman Shiri, Farhad Shokraneh, Inga Dora Sigfusdottir, Renata Silva, Bogdan Socea, Fabrizio Starace, Nicholas Steel, Miklós Szócska, Rafael Tabarés-Seisdedos, Marco Torrado, Marcos Roberto Tovani-Palone, Massimiliano Veroux, Russell M Viner, and Andrea Sylvia Winkler.

**Drafting the manuscript or revising it critically for important intellectual content**

Giovanni Addolorato, Emilie E Agardh, Peter Allebeck, Benedetta Armocida, Maciej Banach, Till Winfried Bärnighausen, Francesco Barone-Adesi, Massimiliano Beghi, Akshaya Srikanth Bhagavathula, Felix Carvalho, Márcia Carvalho, Giulio Castelpietra, Joht Singh Chandan, Vijay Kumar Chattu, Rosa A S Couto, Natália Cruz-Martins, Paul I Dargan, Diana Dias da Silva, Adeniyi Francis Fagbamigbe, Eduarda Fernandes, Pietro Ferrara, Alize J Ferrari, Florian Fischer, Peter Andras Gaal, Alessandro Gialluisi, Juanita A Haagsma, Josep Maria Haro, M Tasdik Hasan, Simon I Hay, Sorin Hostiuc, Licia Iacoviello, Ivo Iavicoli, Kim Moesgaard Iburg, Jost B Jonas, Tamas Joo, Jacek Jerzy Jozwiak, Srinivasa Vittal Katikireddi, Joonas H Kauppila, Moien AB Khan, Adnan Kisa, Sezer Kisa, Mika Kivimäki, Ann Kristin Skrindo Knudsen, Ai Koyanagi, Tea Lallukka, Berthold Langguth, Paul H Lee, Ilaria Lega, Christine Linehan, Giancarlo Logroscino, Joana A Loureiro, Rui Ma, Áurea M Madureira-Carvalho, Jose Martinez-Raga, John J McGrath, Enkeleint A Mechili, Alexios-Fotios A Mentis, Tomislav Mestrovic, Antonio Mirijello, Babak Moazen, Shafiu Mohammed, Lorenzo Monasta, Francesk Mulita, Mohsen Naghavi, Ionut Negoi, Ruxandra Irina Negoi, Vincent Ebuka Nwatah, Alicia Padron-Monedero, Songhomitra Panda-Jonas, Shahina Pardhan, Maja Pasovic, Jay Patel, Ionela-Roxana Petcu, Marina Pinheiro, Richard Charles G Pollok, Maarten J Postma, David Laith Rawaf, Salman Rawaf, Esperanza Romero-Rodríguez, Luca Ronfani, Dominic Sagoe, Francesco Sanmarchi, João Pedro Silva, Bogdan Socea, Fabrizio Starace, Nicholas Steel, Miklós Szócska, Marcos Roberto Tovani-Palone, Tommi Juhani Vasankari, Massimiliano Veroux, Russell M Viner, Andrea Werdecker, and Andrea Sylvia Winkler.

**Management of the overall research enterprise**

Giulio Castelpietra, Alize J Ferrari, Simon I Hay, Mohsen Naghavi, and Maja Pasovic.
